# Supplementary material for: The repertoire of G protein-coupled receptors in the sea squirt Ciona intestinalis
Source: BMC Evol Biol. 2008 May 1;8:129. doi: 10.1186/1471-2148-8-129 (PMC2396169; doi:10.1186/1471-2148-8-129)
Supplement: Additional file 1 — Protein sequences of all Ciona GPCRs identified in this study in FASTA format [file 1471-2148-8-129-S1.pdf]

>ci0100130008
MKEFLVWSKMNRTTKLKLNGACTVPLSMKGINLKRVKIKRLKCGVWGCTISSNISFVSKHTEIKVLKTTA
KYCDRVNVTNDMGNFTWHEAVTGVTVRYNCTEPQQEVSMTENLDPGLDLYSLSVLPQKLHVHNCIKYRT
GYTCDTTQMMLERNISRENAWSVCSNAVGNLSGVIVRDSIGFQLLGEVIGNCSEHLQENMKSSNIALAGY
NFNVTDQKYIYCSISEAIEWTELTKYPELELVCQDLLLFNFSILLFFHSFLFFILHTYITITVTLGVFVA
TTFKTIHLLHPGFKSKTAFSLLLFVHTNMRHLPILHRNGNLFQSSTTTTMSLMEYKAWKNHLQSSQQED
KGKIVAKQVDFLADMTKFKQELKKNIKPDPERHVLVSSIYSVHVNNRNVVNLTKPAVYIFHTQESVSQLN
LDYEHYIAMWNGSGWNRAHHHCIFNHTTRDQQQHNITVIQCDVLATFAIMKVTLTHPTVYAGSAVLTLT
LLLMLITYAVFRNLLLSRDARHMIINTTLHLLVAVLTFTVGVWSISSKVMCYVTGILLHYSSLSVLLWIT
LSSGNICKEMLAAQQPPLLEPKPSKPMLRFYLIGCGIPIIICGITASAKIENYNGDGGQYCWLSWETSLY
AFYAPAACIAIFCILVLLRILATLNCAPSGEMKRKSRKRRKREYSKEFIGEDTPLQYMESSFTQHSNPVN
NSFSKDVENEQSSKTRLQGVALILVLFITTWVTAAMTVAAPRIQEQKISSHVRQTLFNQNYFKFFKQEEP
TPSIDLHLIFSCIFAMMCIAMSSFLLIQHLTSRSDVRRSWRNLCNRRKKKVLEAESNIQINNDIATNIPD
RKRENTVTTAATDPTTTETALHLTTGGESSRGNLTSRHSPFGRNSSAPLPGGTYDTSRASSAHKCAQFHR
EKALSNTLTESGLLVPHSNSSLLLPSDPNNFYTLTEHQHGDSFHSPSQEWGYHGYGQHYYKSSSKPVKMT
NLQQHQLDSSMTEHSFDDSHNNMHTIPVVLQHTQPKIDNKVLYHRYQKMRKALDAKRNRQKKLTVLREYA
QDPLTSNDESPTKPQKSIDKSSEQIPLLSNVSKCHNANEDEMGLDNLVNTDNIITLPMPKPADEGEQNYR
LLLISSPNKKKGETCMQGKYGQRKRVTGDPNLVSSYRRKSLQQNTVPLEPGGSSRSAKRRRPPASRRRAR
QRATRHETQIKAAASHTEATASQVEATAPPVTDNVSVKSRMSANQAAAEARSSGWAMHQDKAHNYLTAQD
LFSGAMHLPSRKSSSCQAPSNTEPQNPGFQIIDMDDNNVGHENATTSPTEDTDLAYAALRNETSV
>ci0100130017
MKIVLLVVLLCLTEAFAAGIVERRTGSPCCQGEQSELSCSKIYTHPESWRCTCSSTRRNLTEITIAQAVN
LDLANVLVNDVLACVTHVATLGIQNGGQGSELRMLRITGNVGITHPLILRSSVEFPALNSLETLEMDQNN
ITRLREDTFQSNLTNLQRLSLDGNQISEISKCTFVGMPELKELILSRNRINLVHSETFSALSRLRTLDLT
GNQIQKVDSFTFAGLYSLNKLQLDYNNIIDVDFSKILTCNQTRITENCTQEVLCKVDSWPLKKLKKLMLS
NNAINSTSSLKLSAAKAVTELDIRSNKIGNFSFDQFTTLCKLKRLNVSYNSITVIEARLNASAEKCRKLE
VSFAGNRIHNLSLSDIRMFEGLSRLSRLDLSDNRISSFDSFKLAGLETLNLESNLLETFSPMWLFSLLG
NNRGKIRLGDNPLKCDKNLVELFEQASCNEVSDREMYSASSVAQLNTLSQVFDDNETLTLPLPGVCTVIG
HGKCKPRITTSMNKCLLRAYLDLASESSAAQNVNIFDKLLGEIEQLYIYARAYVRKQGLSELKNARIQLV
QAVESISERVRMNIGGRLTGNVNKDVVVGLMEISRRRTRLPPTELGFHIDNSGTGNFLSDHGASPIDQRT
TRTGVTTEYCKVRDNIPNIRERNNQFLFLESVNSSMRNINYRCSFWNFTTLSWRSEGCEVANISSSVDE
RGYSTITCHCNHLTNFAVLLNHDTRENPTVEDNEITELISYAGIIASIIGLSITIIFRLSRREFRRGVPQ
IILLHLCFCLLIVYIMMLIYDKSFVVQHMGSCFLVGFLMHYFLLSSWGWCLTEGVSMYLLLVQVYNVNLR
RFLTKATICCYGAASLLVCGSLILSGSSADVAEHPGYIVLDAYRYGDACVVTYPNSLYFVIIPIAVACSL
NLVCFVFVIKGLRQTAAX
>ci0100130791
MKASAVFKTLVCCLCFKVCQLAAPEQGIYKRTLTKTDNSLYIVKTLYRDSIVKVAIVGKDKKCASTEAIE
VFWQVRSSSCFGAFSAPSSEWQSTFINHITYTPQYQSENVRGEYVLNYVINGNQHTTDDKIYCDKNNILH
CHNDWKNITVEPTTKPPTTEAKPDEKKNTQKREVGDPPAVNEPSESSDTEDVVAVVPERSYYLFYLFTTN
IHSDDAYEVTIELKSPVGYLSAHEYPLLTFYMCMCIAYIIYALLWLVMCACNYRDLLRVQFWIGGVILLG
MIEKAVFYSEFLSVDQTGISVIGAAKFAEIVSALKRTLARMLVIIVSLGFGIVKPRLGPMLHRIIVVGGL
YFVLAVVEAMIRNDSSLHDPNNRAVILAFIPLAILDAVICWWVFMSLLQTMKTLRLRRNTTKLSLYRHFS
NAIIFCVLASIIMIIWSWRVHSVRCMVDWPQLWMETAFWHLLFSVILFVIMILWRPTANNQRYAYSPMVD
GNDSDEDEEPMLSGGARETVKMRGGKKFERSGGDKTEEDLKWIEENIPQTIADVALPMLESDDEDEITTK
YEMSKME
>ci0100130090
SQFCRPAIVFTMVVDSVLAAIVVVTNFLVLAVSVRTTIMQNIPGYFKISLAIADIMVGLFVLPGSVYHHY
VLSMSPLPFRSEGQSPHATDYFDQRYLNFMGVFTVMSFSVSIYTMGAASVDRYLAITKPFRYRQGKYLTK
KRSIIVFMAIWAFGFICAIYPVFTKRPYDISGLSLILSTGLIAAVIYAVTMGLPLLAVWVINGMMLRQVC
TDGKNRRTLSAKRRRNQQQARPDTAEGERPENQSGKANVTHSNGAQFVASRLLLSNSFWNCIIYSVRNH
VEVHPWEDILRPRTDTAKLTDRNLCQKWQVQTVHTPLRKRRNGVEVLTYGTLVQARLKTVGSQITAVK
>ci0100130111
GNAISNRQDELHCCNETFNSFSKNWHNEADYLTETLQSLKDWSCPQYNAECSKPTFNFNDFTALVYHRSC
NRTLFESRQDELRDPCIQVALYDAAVQERGKYGYYHEMKRVFIPFCKFIWCGLDAENIHDHSISAWLCIP
TFCRTNIIISIVVCSILSIWNVVANSIVLVVFFKQKRYTSSAGIYKLSIALADIMVGXMYLTITIFTLMV
ACIDRLVYVRNSRTYSNQRSTFVAKIAIATSWVVAIIVAIVPFFVDRYNLIASVMVLSSGATSLYLYIIG
YVIPLVVLWVGGIVLLILTIIRSRKIATEAQTEQEAQTRTMQIMMGVFSLNLLIGLLSAGGSFFLDNIDH

ASPTTLDQNAASTFSSFEFVAILFIVSNSLWNFFVHLGQDARFKSALTSCACSAAATPGVLSPDSPSSFG
MKGTNLGYTNSSMVGSDVMSSDSAVDK
>ci0100130259
MDVNSTNSSLTAIADFRYIGIVIGIVDIILGTLGNILTIVVFSRISNLQSLFNIFIINLAIIDFLTAAFM
MPLNVAGYVQMNWPIGGPDSVSASVQAYVYFCCGYTSITCFMLITVNRFISVMFPKHYKSLFSRCGILVG
LVSSWLIAPAFLLPVLLGSADGLPIIGWNQKQFLCTFIRVTGKWRTYMQVMITSNITWLNSCVNPFIYV
ILNPDFRMEYRIILKYLWLKITCKL
>ci0100130320
MLRFTKKSIGKQQPQGFQSNKQGFIFTHTIILICLSFAKSAEARNTTTATNRQLYLTNFTTQGLPNVSEL
FPNIENETNGSNIAYKVGLSILLGSICTVTVLGNLIVIFTAILFRQLRTIPNMFIISLASADLLMGTIVL
PMWSHYVIGDFKWSLGFFWCDVWTSIDVLSVTASIGTLCAISLDRFVAITMPFKYATKMTRPRARFIIGF
IWMVSGTIAFVPINLGWWKTNKPEDLECYDSPTCCEFRPNVIYAVVSSCISFYIPLIIMICAYSVVFKSK
AMFAFWRPDYLSVKIFAIQKRGVLPWEKREHRAVITMGIIMGTFVVCWLPFFIVNIAQVFCQCVASTPFL
MVNCLGYTNSLFNPIIYCRSEEFRKAFKRVLMCGLCREGKTTCIHVMIPLHIVGETSTSVNGFVTDELY
PQENTLLHMNGRNRSRPSSGYMELTPDV

>ci0100130340
MLPQTVILPFILALRFCKGSWTEPVSYKHPGNITIAGLFPLHYISDRPDNYSLEEAPEALVCRRYNFLGF
RWMQAMIFAIQEINNRSDILPNISLGWQLFDTCNTHARAVTQALHVQNKLYPENLPLPGLHTGLSALIGP
TASDKSIVVSNILSVFDVPQISYGSTSRLLSNKNIYNSFLRTIPNDDEQATAMVELLLHHNWTWVGAVAG
DDDYGRPGISRFKEEAEKHGICVEYTAYVSESMSEEHLKQTAREIKNYNATVFVTFISATHLVPLARYLS
EYSVTDKVWIASEAWATSDTVRAIDVFQGTLGIATRGGEMPGFRAFLEGVSPKTQPESPYITEFWEKSFQ
CNCPDTVCAKTENDGGMSSHNSYAENCTGNESLTGDGAITSAYLGWTDLEKSYTTYLAVYVVASALHNIS
TCVEGKGLLKNGACPYLNNLTTWQLLSYIKKVDFFPPGHDYKTHNDTVSSVMNGDPNASYKLVSWIPKMN
CRGNITVDFVHVGNYSSMVTPKWSINESAIYWHKHNDIPKSVCNADCQPGYYKIQLQSACCFKCAECPSG
EYSVDINSPVCTPCNLTHRADDNRTSCIPKNEEYIRWSDAFGVTFCVLSGIGILLTLFTAGSFMKMRRTP
LVKATNRELSFLLFLSIFCCFLTPLLYLGKPQDWNCKGRTVTFSLSFTFCIAAILVKTLRVLTAFEARIP
VTYKKRTKWLGEHVQVFVVMVVSLVQVILSVTWLVVDPPKVMLSQSKVWAHTSDISLIVECNVGNLVIMG
FVYGYLCLLALTAFVFAFRARKLPENFNEAKFITFSMLIFFIVWLSFIPAYLSTTGINATAVSCIAILSS
TFALLACIFFPKLYVIYWTPQRNTVEEVRQNTATHAIRSSRSVAYSVRIKPPDMSSDSNKSMVQETSPPR
SNFYLLFYMYGRNANDRNPFKDFDNSNFNNSNNINLSSVSKASNRRLKIQNRNRSHSLQPERLRMNLNN
QDIYEDVLTTTFSKDINRFSGQSEKDSVQSESSSTSKLNMCNTVTCADDTESTTFNVDIAGKISTDTAI
TDRAENYEKILFDDGKRINLKVAVKVNIQQTACDKNAPSVQLNNNNNHTPLETILPTSEQLANRPLSECN
NTGAELSVQSSRDDITTTNNMFDSSSA
>ci0100130574
MTDNWDDDDFGTFESADLNKGSAPSSATVNTSTNLPAWLLEAQAKPPISPTHQRDEPNHTAKASNYEQLS
LPTSVASIFDSTNTSVTSEEATGDTGHDTVSATIASPAVTEYAFNAVFSDATSAHSTLSTEEKGSVFVKK
EKEKKAEEQKKESDLVVQLRGQLDSAMALKEQSEMSLNELREELNHKVEELNKKIKRKEEEHSIMMMQLE
EKKSKELEQLQQSSENTMREVTEQYTTLCNQVAADQKLQFEKHLLEITEKCCTMLNQQNDLMTQQMTKQQ
SIFDEKLSSAMNQCKDDLDLLVEEKIKMVEDRCSSALEAAKIISQVQIEEDFERERSFNAKSKRDLVAEL
TLKHKEELRASVEEERVRGNVIKRDAIEETKKSILRISREEQQEIELTRERHLNAIGILLTSSQEHLRHL
RDDVTSNPNATKHIGNMSAKQTRLFPDVLSGNSINVSTLPFAENESCVPLKEPFIHAVSAVPALVVIEN
LVILLALVLYRSRLKHSNVYRYVASTLVANLVTSALGFYHFINYYYGIESAHPNHWWAFRKGMTLALSLV
LCGNIGLMIYGIEDSTYIVGRVSMQTSEQSTRLDPKFRRRKANALICVVWALPTIYGLLAMTDWNCTNLC
SCTLSYKSGKYLCPVGRCSHIYTPMSKSYLLVVVILWALECIGLLFLLCRSFCNIRGRGDGSESGGRKIS
IRKALGKYHASHHTVTILFILFVVCTAPIMGLFFADFIQQDAHFSHFLVNCITPLPLVYCVVSPILITQK
LSGVRSALLMVMTFSWLKPQSNTGPKNGEKKTPQTPDSTNPTTH
>ci0100130612
YSECDGFNDCGDWSDEKDCEWICGANDYVCDCYSNGNCDLTGIYYFWPCYDRSYKCDTDNDCGDWSDEKD
CNYQCGSNELACDYLESISTCYSKEQQCKNMGKCSDFSDNKNCTCEANQLKCGCILKPDNCTSALGCIDI
HEVLNGLTDCADYSDEAYGSDNCFVDGKLKCFRCLDGRLMISAKQVCDGVIDCYDLSDECLCQDQSVCDN
VLGKSRQSCLPNEMICNGVCMEIDNVICNKMVDCNDTFNNMYCMLMMHRDEEIYEYKFCPQDAEESAFVT
ASVCDGLPECFDRSDECNVGCPSETHFCLSSLDCHTKLVNAESSLRIESYCDGRPFISPGCEPGFDEKNC
TGRFYCKNTSSLVWSVEQVKVCDGVLDCVDGSDEVVELCKNTRFYCLNKQPLSVDKSRVENGFKDCSDGS
DEEMIGTVFIRILMWIMGILSLIGNGLVFLFTVIEMVRKKNEPLKTSFLWFVINLSLSDFLMGVYLVAIS
SKGAEYSGRYCYHDAEWRSSNLCSGLGSLTIISSEVSALTMAVMATFRLISVYKPFKMANVKRVAYMAPT
FTIWAVGILLGTIPLIQTSSAEFGYYGETSVCMPKVFVTVGEAVWEYSAFLISLNFCLFLYVVAVYIGVF

KRSTKMKNNAQTSNKLQKTISLLILTNFCCWIPICIMAFLSLSGVKLDRIVYVISAGVLLPINSVINPII
YSDIGRIAVKKFIKNFRKPVVVSTPRMENVIPPSPNTNRITTITSL
>ci0100130776
MVQGENITDESIVNGVNQLEVIASGPDLISGDTKEKFVEFFKTISDLAAEGKAKIFDHEGSSSVSYSPVV
KGVNGNNSQPAKISIPNEALVQTLSKGLARKKRSTENATFRLSLAMYNDASLFPTNDNVSNVCIVSSFSC
KKQLLYSSWCKYWDFTTSKWADDGCCLNETANPTQCLCNHLTNFALLTSVQGPTDVVLSVISDVGCILS
IIGLFVTILVHALHRYLKTLWNILLNICSNLLIAYFIFVVGVSQTSNRTGCKVVAVLLHYFFLTTWCWMS
VYSYDMYMSLVKVFRGSESKLLQRMAAFAYITPAAIVIITAGVAMDPTLSSSYIADHMCWLTGGSLYFGF
LLPVAAMLMYNFFILALKVKDTGWFRRLHRNQSVPGAMLGSVTMTSLMGVTWVFGYLLVISTDVIYVTVF
SWLFALSNTLQ

>ci0100130804
SAAEPLKTTDIHNIIVPTVRSFSKGLDKYSFISFAEISSTGSDYVTSLLRVFEEETAPEIRDNVAHLVFP
HSENGLSSSSDNAVYETWGNKIRPQQLKQLQRVSRNLNSILKLDKLSTEFYLDYESSRHHVYQHVMVVMV
PVADPPQQLNVFVNVLLDTYLRHHQHLDVNQVKIFVVCDEETLLYPFENSTNVTFPATQAGFTAISNNKC
PNIWNDVRDSPINTPEQRQQVANDLEVITSVSADLTTTDVTQTSEALTEIVNTTTLTPEVRENMNVTCF
ILAWQENDSCYNMSVLFHTPRANLLVNVFFCISDNLENPLVTTRLKKLLLSVLPKDTYTHNGIIHTYLHL
LWFRILATLDSVGEKVEVDEGEEYTEVSGSVAIAVVQPTEQDRRISFVAFPDDKLFRSNSSSTFTMFNNV
TVNSDAVLSATVVNANGPLNNLDPPVELGFGTTTVTTTNIVSSAFKLMPQNSQELCFFCQVVKKILLIR
SGRYCIILFVSLQGLIAGRSVCVYWDEVTLTWSDVGSVITNSSNTTLTCTFNHLTNFATLFVNDTSISNS
HVLDILTVVGCSISGASCLLLILIFIFVPKTRLKGRKKGAILLANLATAILLLDIFLIVSEQEIVTSSMT
SCLAVSVLTYFSMMSVFTWMMVEGFSIASIIIYLFGNWFMIASCLWGWLLPALVIMFTTIFNIDMYKRTD
GDLTNFSTRCYIQPGLVLYAVLIPAAITLGVNLMLYVFLTYKVTCAKRPNFMAGKSKDLQKNLLFSLTLF
VTLGLTWIFGFVIIPGNGDASFAFSVLFTVFNSLQGFFLFLLYVVRQKFTRSAISEQARRISTYAIPTTR
TTQ
>ci0100130945
GAWSTKGCEVMSSNHTHTVCSCTHLTSFAVLLQVENELSATHQRALSIISKVGCVLSFIGVTLMCAAFIK
LKFHTENMKIHFNLGLAVGFADLVFLFEEVAESSRTGCVIVTVLLYYFNMAVLAWMLIEGVHLYSQVVVV
FVSARRWVKRYMVVGWGVPLFILTVSMGILNVKLGTNGICWLSPSDNSIWAFAAPALVVILINTVILITV
IKVIINIQPEKSNHAKVSKLRNAVKGSIFLFPLLGTTWIFGLLSLSQNTLLFQYVFATTNSLQGFFLFVF
HCLCNSEVRFVASTLYGMQVREDARRHRWFVTSSSTQGLGTRGQRNQPGVLSLNVANGLRRFRNSISFE
TPTPSTLSNQESETEDSKKAGKLGAILSFFPQMFSSKFPPTIMERREYEETKLTPTGTPASGGSPRSDTR
DAEKNVPLPGMPVKNDKFITSLSQLSLPKEAQVKLNKLLADHISRTDADSPPDVGELGKDEEQKMKMSYD

SGLNSIENESELEDEDGWMEGSKNVKED
>ci0100130986
MVQISEAWQIVIPIVIGVVLFIGLVGNSLVIYVTVTTQRAIKHPVHSMLVLNLAAADLIYLIASAPYQAM
SNSPISLGVDSFLCPSLHFISVLTMSVGIFTMCALAWMRFMALIVPFRFQGSMMSTRTFGCIVLVVVWVI
SCVVSTPNLVYYRPKPDESGPNQTDRLAGICEWTDGKAADDYAIALFVITYLVPFLLVANFYFFIGREVC
YLVNRPRRVTKTTIMVLCLNIAFLLCWFPHHLYRMMKVLLIEQVGACMSYMHSCINPVIYTFAAPGFRR
SLLESITGRRC

>ci0100131120
VILLILSLLVIIGNISVMLVIYHSKSRTSQDVYKASIAMADLIVGVFVIPSFAYTYEMFNKSYLNAIGFF
TTTSFIISIYSLMVASFDRFSIVRKPLAYSKDGAKLLAIRATVILWVLSIILGILPLFIGPSLRYGLVAS
TLISTSGTASLYFYVVALAFPLIVMWVLSISTFVMTVRHNNRTRKLVASKNKTLSSRTEIRMARTLGIMV
GVFTLSLLPVICVILASLFMYNKLLTNVHLLFYAAAVVRISTASTVLTGNATSTFQATITECLNVRGQVI
TNRKFELQCCQLFSNRYKLYWFREGNYLTEALETLKTWRCPEFEEDCTQRIYGFTEFTQRVYDRACNRTL
FESACLPTVFRVLSDAGVNVTDQNWRHALRNVQLEMHALQYPCIQVALYDAVSAARQSSYGFYHETVHLH
TPFCHFVWVGFDAETTVQHSIFPWLFLRTE
>ci0100131137
FFIIFSYDEKIMVCSWDDTYSRSFTIFITVGAIVLPVIVIALCYWKLWVRSLSMGNSAFQSNEQHRRSVR
KERNLLQTLATTVAFFCVCWLPYGLCVLIDPVGVPPLAKKIQKEVYTNHPYTEFWQQISNTVEQPRCYYS
YSKAKLGCMSFNLTTEFSPTQLSNVTEGTKLNAEIGAVQVVELIYLTILCVLGSVGNLVVIFSITLENR
VHSHGNIFVINLAIADLLVIQVTGFVVPTVMINVIQSANALPPIACTIVGYTVTVTCTCSLSNLTVIAIN
RFWAVVRSKTYTQNFTRKRVYLMMIATWVWSNLLSMPSLIGWGRIG
>ci0100131140

NFVGVRRLSRGSGGGEVEYHGGGSGNGQSDYAKKHKFSFPLREIAGKRSQKDSNGYSETEPAKTENESRT
NQTECTRVLLESRKETHECMRIALHSCVQNDQPCFWLNVWRACSKLDNVVDVCNNYLQFALSALSRIMK
TVFIFTLLLLLLLLAPNHAQETGDSNSLDDVTSNDVAGNGLDFGIGDDVASDVGDGLKTNDDDFYDLDLG
EESDKMGSLIGNGDPNIFADIXITLAIIYAIITFVGLIANGVVFFVIFAGREIGKTVTAMYVVQLAIADS
ALLLTLPIFGLQKVAMNWSFGAFTCTLCHSIKFLSYYAGIFFLTAMSVDRYVAVAYSTKSHQLRTRRRTM
IVCLVVWLAAGCMTIPILIFLFGNNQSHEYTCSHKHQKKVYKTWLVVDFVVGFVLPFIVITISYVMIVKR
LQTSEDKIANMTGKRTISMRKRVTRMVAVLVVCFVVCWLPNHLFTLAKIRGLDLSVTPCYGIEHFLVAFS
FSNAAINPILYSFLSHNFTTRFRDSVAMARRRMGRKGSMATGVHQQGGTHSGDHQVRSLFKTEHLTIIKI
TH
>ci0100131143
TDKRQQTHAAHQCNNLYWIHDNISSKCYCELATFRNKVLRAFDVRVHMAASNMAWFNSSINPIIYVLLNR
RFRKEYKRLVLMFFGRVRGSFMHDSLSTTTPQQKHKVGINVSMQPSAMPTLQVARAVFQFLPLVIMVVL
YSIIFTRLDRQRERIHATMRKGRSASNATGINSVSVSEASPTKSKRSVSIVKDKNNLRKSIMLRKTSSAD
MSNAKMDAGLGTTMAATVVSTVAQLNVTQLNATQPDIDYLRASSFRYFGICVAIWAIFVGSIGNLLTIL
AFFSNSKLRTTFNVFIVNLSIVDFLTASLMLPFNLAGYAIWTLRFYSEISSLQLFLLRLYVRCISSYYYR
QSIHWRCKAGKV
>ci0100131249
MSVNARNYVKGDVHTVSVPVRFSLLRGPTRKNAKLYYRPVCAFLVPNSQEEEWDTYGCTAIQDKDYVTTC
ECNHTTHFAILMKAFGDELPEPFNKSLEVLSMILSAISIALLSVTILIFITYRRVLLKDRMLIHFHLIIA
LLGGYVSLLVGSAFATHNEQPTIPCIISAMVSHYFFLVVFMWSLVEGVYLFYKIILVFHKKSIECLQRFA
PLVGWLTPALVVIVAFIVSRTTCDDYSVEKNSTYIDQYANATTCFLTSHNGMIWSFLGPVLFIIALNMVV
LVRVSMVIYTSAYTSTRLSAIGNSCRISNSLKRSSRGALILMPILGIPWIVGLLANLYANNSNVGMVVGT
FGSYLQVLLVGGQGIAVFVFYCFYNSEVRGAYMLAAKKRKSEDSLDAARRKKNRASSDVQRAGIYMSMER
LSVSTLNLLNIRRSTNGSIRDKQSYKIGAEKATTNFMLWPSWRNRIKPVNFGEVDAVINAFTNLTSIKI
SDVHKFLDNANVLLDSTKKIASYQFYEGMGNDSGGKLIEKTAKGISNLLIQWKSIQKIKQKDEVSESLFE
RTSMVEKPTTNESHVRTLMFDTMESITQSIAKRLLPPDNGSELLIKASESQVDVQVTLSTNPTSLVNQTH
SELSRTNNVTVIMYPATNSSTNDIRSFAVISVFYDFPWSSDTDRYMVVFFHRTPFICFS
>ci0100131416
IPGSPTLAMTSQRNSLSAAPSIHVLIDCKKCSSETDPSRHNTEEPTNIEEPTPKSNEIANLIAKERSGII
SEPVTPRTKGKDNLGVKVEIKYLQVGKKKRINKKEKEVAPSPTLHSNENSHCQPTKSNLRLKRQYTAMQL
EERKRKSLDRKLLRMSVAICIVFMILFLPSVAINLAQVKDPRVHMFASVITWLNSCVNPVVYCFINKRMR
LQYKNLLLKFWGRVCVCIGKESTHSAHRRTRYSRSSQHSESQKSSTRNRIMGEFENTTDYIVTSAINFS
VATTSEVDVSAMRLLGIVIGIIDIFAGTSGNLLTLLAIYNNKRLQTTFHIFIANLCVIDLLTASTMMPFN
VTSYIKQEWPFENDFTCSFMAFSYYCCGYTSIVCLITITVNRYVGVCHPKHSNRLYTRNRTVVVIIISWL
FAPVVLSPFFLPGEGGQRGFGWVQRLTLCVFKITAKWQHYYMEVNVHFCSLVNSVKWLYSFY
>ci0100131489
MSDLHSHNRGSYEKATSHSTLPTTDHMSGSFGGIDDFERPRSFRTQEQNVVVMSPLSDTKPVPTKRNGMN
YRVDRVRPPTGGRTNPSLVLNEEDVVRGRYMDNTTVRGCGNIKEDFTNLCDLDALWGIIVTGLAALGLV
LTFVFTVVFSVNLKRYVRHQRHRYSTLLYFVLLGVFLLFAFSIAFVVKPSVIVCYLRRIGFGMAGVVVVA
PLFVGITRTWRMSYLDELRCGLLAVSAACLMLVEAIILAEWNILKQPVLANGRCEVQDRDLVYSASYNGF
ILILFFLSTLVACCKRDHDRLSGFVISCLVTSIVLILLSAAWAAMLCYGNEKLGHATDWSDPTTAMFMVK
HGKYDFTKKNKQKTYKNAYIYYNVSQCKNTK
>ci0100131543
MASSGSQFWILVCLVVRSNAVMVTSLKNNKKAVVLCITVLLLPYLLSVGLYFVSHSAVSYLFIVFWVVPT
VCFIFMSFRRKTHKPNKFVNQSPASRNSILRRVRMSSQFFFTPRYRFRTWTSEAFYVVSIAVDCVIIGNE
KLPFWSCFVVIMLSVMSLVGNSFVVGIIYRSRRMFRLVNFCLCFLAINNLVGSATSLVSSII

>ci0100131580 (JGI model)
RLYQLECAKYNQNTEDWDKKDCEFEPGVGTTPSRCQCDSKYNPEVYFLVNTFQKEVYFPSAKVLSIVSKI
GLWISLFGVFLTFVIYISIKQLRNRQPTIFILNICVCLFIVYITFLFGIQATETKTTCDASAMILHYALL
AFWFWCGANTLNLYKLIVKVFGSPDTLTPCVLYSTCYGLPLLIVGINAGATIFTTDLSLPEEDSNYRHSA
KCWLRHYSLYFGFLLPMGLIILFNVVVFVLVISKLTCKRDKIRSSKSQKNSAKEGLSLAVTLCFMMGFAW
IFGYFLLIDGDLKLIEVMSWLFTLFNAGQVTLFIMELPPDELENEIILDTMSVKVRCSYVFILWKRPHDT
VYKELTEDKQNTCQMWRMYVTQFVISGNNNSYGGKVQLINNDIKVTPMQPVPSKPQVSFNNPLLNLKSC
NTDIDDCAPSPCSYNSTCVDLINGYYCICPPGLNGTNCSINIDDCSPGICNNGDCIDQVNNYTCSCFDGY
TGDKCLIDIDECATDPCVNGTCVNLVNAYMCNCTAGFDGTNCSNDINDCAPNPCVHGSCTDRVENYTCAC
DTGFTGRNCSIDINDCDPNPCDSCGNCTDLLNDYNCTCPAGFSGKNCSIDINECTPGVCGNGTCVDMLQG

YYCNCDIGFNGTNCTNNIDDCAINHCANGGTCVDGVANYTCQCVPGYTGYNCSTDINDCDPNPCDSCGNC
TDLLNDYNCTCPAGFSGKNCSIDINECTPGVCGNGTCVDKLNGYYCDCDLGFNGTNCLNNIDDCAINHCA
NGGTCVDGVANYTCQCAPGHTGYNCSIDINACKPSPCVNGSCLINKNNYTCECEAGFTGMNCSIGNNILI
VYFCYSFFLMPHVFLMCSWCSLLTFPNVFLQSTDIDDCKPNPCVNGSCTDQVNNYTCSCDPTFKGRNCSQ
DINSCTPNPCMNGKCSVDKNNYTCECEAGFTGRNCSIDIDDCSPNPCVNSMDCIDRVNDYQCDCTPGFVG
KNCEKSYDDCHNVSCNSFECVDGFLSYTCNCKNGYSGKNCE

>ci0100131580 (proposed model with corrected alignment)
CNTDIDDCAPSPCSYNSTCVDLINGYYCICPPGLNGTNCSInIDDCSPGICNNGDCIDQ
VNNYTCSCFDGYTGDKCLIDIDECATDPCVNGTCVNLVNAYMCNCTAGFDGTNCSNDIN
DCAPNPCVHGSCTDRVENYTCACDTGFTGRNCSIDINDCDPNPCDSCGNCTDLLNDYNCT
CPAGFSGKNCSIDINECTPGVCGNGTCVDMLQGYYCNCDIGFNGTNCTNnIDDCAINHCA
NGGTCVDGVANYTCQCVPGYTGYNCSTDINDCDPNPCDSCGNCTDLLNDYNCTCPAGFSG
KNCSIDINECTPGVCGNGTCVDKLNGYYCDCDLGFNGTNCLNnIDDCAINHCANGGTCVD
GVANYTCQCAPGHTGYNCSIDINACKPSPCVNGSCLINKNNYTCECEAGFTGMNCSIGNN
ILIVYFCYSFFLMPHVFLMCSWCSLLTFPNVfLQSTDIDDCKPNPCVNGSCTDQVNNYTC
SCDPTFKGRNCSQDINSCTPNPCMNGKCSVDKNNYTCECEAGFTGRNCSIDIDDCSPNPC
VNSMDCIDRVNDYQCDCTPGFVGKNCEKSYDDCHNVSCNSFECVDGFLSYTCNCKNGYSG
KNCEIMELPPDELENEIILDTMSVKVRCSYVFILWKRPHDTVYKELTEDKQNTCQMWRMY
VTQFVISGNNNSYgGKVQLINNDIKVTPMQPVPSKPQVSFNNPLLNLKSRLYQLECAKYN
QNTEDWDKKDCEFEPGVGTTPSRCQCDSKYNPEVYFLVNTFQKEVYFPSAKVLSIVSKIG
LWISLFGVFLTFVIYISIKQLRNRQPTIFILNICVCLFIVYITFLFGIQATETKTTCDAS
AMILHYALLAFWFWCGANTLNLYKLIVKVFGSPDTLTPCVLYSTCYGLPLLIVGINAGAT
IFTTDLSLPEEDSNYRHSAKCWLRHYSLYFGFLLPMGLIIILFNVVVFVLVISKLTCKRDK
IRSSKSQKNSAKEGLSLAVTLCFMMGFAWIFGYFLLIDGDLKLIEVMSWLFTLFNAGQVT
LF

>ci0100131605
RGCHVVNVNTSNKTVTCACNHTTNFAILLQVVPPKLTATDTATLQLISYIGESLSVFCLIITLTTFTAFR
HSLKSERMVAHFHLVISLILFHVVQLFSAKAEQYKDTTAVPCIIVAFATHFSLLVTFMWMLCEGITLHLN
VVNVFHAMTKFKLARYFIGWDYPKMVVFATYRGYHFIEWCWLSTDNYLVWALVIPALLVLTVTVKMLSDN
INNLIKIYFLHFPFRKSLKGAFVLMPILGIPWVFGFIPQSLTHVALLYIFTVLNSTQGIFILLFYCILNT
EVRTVVVRCLRKQNLLHRKQKSWASQSNYRHSVDLRQINRNDNSDFDQQMGVERSVSVHTSSTYCGGVAP
SSGDSLTGSFQEQTSFLPQTTVKHLFVDSYARKWPRSTVARCPEDACTLIRSSSLRCVETDNVNNGEPTQ
CRKTWHSSNFQNFSVLRYCDEKDKIKKIICLLNPNTYIEAHYKIPFISTFFTEPDPVVSNNVSAEDILG
ITDGEGVNYIDADDASAKLGEIADTLRDTKGNLNLGVLINSVEALKPVNYSAVSSTGIKRLITNFVTIMD
DMVDEGRADEWKEIKEEENGPESSQPEFATTTIPPQQKQSDPHSLLFITENVLQRFVMDSTATLWSKFDQ
GLTVTTNNVISVIEKPISNASNTSTQDTPTLTKVNLNVDPDVASDESTVVITSLFPRIINLISEFAKLTN
CIYLQFVPSAPVLSATVLQKGKITNSAVHFIVPYNASDIPDAKKSDEKYYFNCMYLTLNNDWDTR
>ci0100131681
QTERRTFLMLLAVVMLFVVSWMPLHTCFHCNQRFISDTSTDTVRLIVNWLALSNSMMNPILYAFLNRSFR
TGFRHVIIVMLNLMLVGIIGNIFVIASVKREKQLNIPGNFFIVNLAFADLIITGISMPIILHNVLLPASM
IGYEQNIFCQASAYLKIIALHVSWYSFAFIAINRYYSVCRPIQYPYHFKRDRIMKTIWLIWLWVFIINFP
VMVGWAVPIVYDNSTNDCVLGIQNNGGYLFANGIFVVALPLLTIAFCYSLVFMAIIKNRRR

>ci0100131721
MGLTLLLMPIIPCVLYAKIYCFVRRSSEKLKPVYTPPLVANNGGTEDNSRILPIHKTETCNHLLVRPKIS
QSIIQYEANGNVSCRKTDDSWQGDGVRPPSNNRIGLKGWNRIREGSWRRKAMVTRIFTKNSNDIKEEVPQ
EKRKISSASKAKSAARRRRYRDMRLLRTLVIILVLFLVSTVPLGLLFVVSYTEQDKRYVGTAKVLLTTSL
LNSLVNPWIYFWRFPEMRTAMKKVFCRACAFKGSPSHKNDKRYTGDGAMGVHRRVAGGVSFDTNSRAGRN
GSKPENSMGKENTSSISSNALMSNALGGRIFTEGSDAIGKNRFCLKSFTANFSRYGSSQGTNNSVIQDS
ILSNYIITPCQQDKAGAGSFEVVATVAGAGIGITNIVVIAAILWGGRKLHKATFFCICNLAVADMLAGFL
LLWIFGLQKILLPFRTPQSELVQKSIWTMTVWSSMLSQLVIAIDRYCYVTKGSPGDGGISRSTRKSDIWG
NRKRKR
>ci0100131758 (JGI model)
DLIRNPILQIIVWLVAVISLGGNSIVIIGTSRELWRDRQVEKLGRLAKCNRTFVLNLAVADFMMGLYLLM
LGIATAVKSDRYCSSDRTWRTSHLCSIMGIMAQFSSQTSVLLLVLMTSYRLYGTLRPFQVEESKLLGGAA

FLTGCSWMLSFMLSFLPQVRLFSVFHACYYNKYPLCQPFFYPNLGETGWEYMTSIIVLNFVAFVYIAVAY
VVIYRASSRPNVRKSVRRRSRQWRRDNRKMQKKVMLLVATDMACWLPICIIFLLRMSGVQVHDVVHGITG
TVLLPINSAMNPIIYSVCDGIIDCLDLSDECLCLSNHENRPQTRNPICEQLTIRHPSDIPVVSEVFIFKG
SNATATSCDGEATCEDFSDECDAGCWDPPEYCGVIKNGRFQCTEDTNSRFQNSSIGHALDPSLVCNNVID
CPLSNADETNCSTSSHYYCWSK

>ci0100131758 (proposed model with corrected alignment)
vcdgiidcldlsde clclsnhenr pqtrnpiceq ltirhpsdip vvsevfifkg snatatscdg
eatcedfsde cdagcwdppe ycgvikngrf qctedtnsrf qnssighald pslvcnnvidcpls
nadetn cstsshyycw skDLIRNPILQIIVWLVAVISLGGNSIVIIGTSRELWRDRQVEKLGRLAKCN
RTFVLNLAVADFMMGLYLLMLGIATAVKSDRYCSSDRTWRTSHLCSIMGIMAQFSSQTSVLLLVLMTSYR
LYGTLRPFQVEESKLLGGAAFLTGCSWMLSFMLSFLPQVRLFSVFHACYYNKYPLCQPFFYPNLGETGWE
YMTSIIVLNFVAFVYIAVAYVVIYRASSRPNVRKSVRRRSRQWRRDNRKMQKKVMLLVATDMACWLPICI
IFLLRMSGVQVHDVVHGITGTVLLPINSAMNPIIYS

>ci0100131828
MGLLVLPSVAHNILNGIKYQQSDYKQLMLYSEHTTVEGAIFGIVGIISSTTTVFGLFLLTIDSYLSVRWP
VKYRVGDIMTTPRALFLVAFIWIIGLAVSALPILAKDFLEYRLVYFTFMYMPYLKPIPVSMPPSAIPPG
YLQNINRVGDQSTEVTTTQPVNPNRNQIIVETPYDPTSGAGDVTSTSDPKTITIPYILNPNSQCGVFPAC
SIGLRINEKSELTAVTCHKPVVGLLVAIVIILSIVIVLSNVLIIVVTMRTRSLRKPHGYFKVSLAVSGKH
SICLKNIMTEIFYNIVGLAAALLFEDFYS
>ci0100132083
GNIFIINLAVADIFVTGFFLPTVLANVIYTGNSLNKPACDVAGYVITLTCVCSVSNLTVIAINRYWAVVK
NKTYAKMFSKRRVYIMVLLVWVWSNLLVIPTLFGXILCSIRYDEKMMECAWDDMKNRSYNIFLVVCAILL
PVGVIVFCYYNLYNRGAWARSATSVGSRSNDRQRRSLQRETNLLKTLAMTVVLFVVCWTPYGFVVVFDPF
GVAPTTKKIVAWMGLSNSVVNFIIYGTMNPVFRKGYKHLII

>ci0100132112
SRGGRRRRGTSDYVYFQVPNRSVRISENLPAGSPVTTMRAYARNEVATQNVVYRLEPRQNRHSLELFRID
PHTGVITTNSSLDREDMSIHFFNVFAVRPDINQARGTLKVYVIDVNDNSPIFERPSGYNVAIRENQAIRT
QIQTVKADDADSGKNGELTYSIVRSVPHTNAFAITQEGNLIVNGLIDREAHSQYLLTIAARDQGSPSRNS
TVPVTVTIEDVNDNHPQFTRAQYNVRVYENARVNSIIANISATDADSGMNGLVSYVLYSGRRTFRINPQT
GEISLIARLDYEVTKRYQLRVRATDNGRPALSNTSGLVNVQVLDINDNTPHFISSPYNKVVLESAPVGSK
ILQVEAEDGDALRSSSRIAYTILNRGSPVPFRIDLQNGTIQLSQKLDHERQGSYTFTVQASDHGSPRRTA
TTQVTINVRDVNDNAPYFTSNSYQKRVAEDMALKSPIINIEARDPDTDATISISYRIIAGNQDRKFSIIS
RNGIGKISLAKALDYNEQRHYRLTVEASDGSLKNTTFVYINVTDTNSHTPIFEPNYYPVTLPENVPIGHL
VVQVHAVDGDIGDNARITYCFSDAVSNFQINSNNGEIRTRQKLDFDNLSKIMLGVIARDNGIPPEEDRAV
VEISLTDINDNAPVFQQTKYQGEVSEDSRVSQRVLTIHATDRDSSNNGEIRYTFEGGNNGNGYFSVDPHS
GDIRTALPLDREHIDKYDLVAFAVDQGTPKLSSSVAITINILDVDDNPPTFKNDTLYFKVSVKEDVDIGH
VVARIKATDPDQTTSDIIYDIGHDPEVLRYWKLNPDTGELATVDVLDYERKRNYTFIVSALSNELVSQAT
VHILVQDCNDNNPVLSDFEIVFNNYRTRLSDSFPVGVIGEVPAYDPDVSDTLTYRFLSGNDGKLLLLQHN
TGEIGLNKTALDSNRQFSTRMRIEVSDGLHRTPAWCTLRVTGITDEMLSHSITVRVLHMNAYSFLSPLMY
SFIDGLANILNTTSKNVFVFNVQNDTDVKHEILNVSFSAKKERGEFFTSKYLQDQVYLNRQRLSELTTRN
VLPFDDNICLREPCQNYKRCSSVLTFDSTAPFVSSRLMIFRPIYPVYKLKCECPIGFTNDIKCADEINMC
YSSPCVNNGKCLSREGGFTCICREGFAGERCEINMKQGRCIDYIGVCRNGGQCVDGPGSDEFHCVCAHPN
HSTATCELRSREFNHGSFLMMQGITNQWHFTLSISFSTTQENGLLFYNGRLNHEHDFIVLELVNGQVQLN
FSTGQNWDVVQPFVDGGLNDGTWHTAKVEYYNERKPGPLPTGIRSRYGPSHNKVAILTIDPDICDPMVAQ
QWNETYGTYSCSKQVKQMGQKSSLDLTSPLFIGGVPDLPEDFQVRSKSFTGCIRDLYLDGKILDLDKFIT
KNGSLIGCPSTETKCRPNSCYNGGTCTSTWEDTHCDCKDEFTGKKCQHQSELFMQLLGVGCVEFTQKNRL
LPFKLPWRVQVDFRTRDLNAIILHVATAGIQMDLKIENGYLWYVVQSHVPIRLGISNAPINDGEWHHVTA
DWIDSGSQRVFVKLNLDYNAHQVFLCLFASLRQMSISRVMIGGKYLSGRTISPYKGCVKGLKLGPPNTMY
PISDSSLRPHGGATHRQGCRVTDACRTHSCPRNSVCVSTWGDYECRCNTGYIGQHCTSVCSLNLCQNGGI
CRNNISATYGYSCVCPPGFRGPSCTDRYTTECPKGWYGTEGTCAPCKCSIQDNFDEACDKISGECICMSN
HYFVSATGKCEPCGCYRPGSVGLSCTEDTGKCHCVSGVSGRQCNLCISHLAEITNDGCEVLYAGKCPRSF
IGDVLWPRTDFGKQSPVKCPPGSKGIAYRMCDTVQGWLKPDFSGCVSDAYYSFGVIVEDLENNVTEMSSP
KAIELASELSVATKSTNKLYKKDAINSFQIVQELLTHQSKQKDFGLAVAADSAFTDHVAEASSALLSNNS
LHFWSSNSDEKHDSANPLSAPVFIKAWEGYTSTLLQNLHKIFYDSTVVTTDNINMSLYSVDRDISLFGEY

IQLKFLIVFVIFGYSYPLIEGRAENEVSVKLPKYLFSTVNPFNITSQKAVGVVTYNNLNKILPHTFEDDR
TFRIPKKPSINSMIVSIQVYSSKLDHKPDDGMVTKLLEPVKIGFKLINAEAIEPQCVFWNFTTKSSPSST
GGWSQRGCTRSFSNKTHVTCDCYHLTTFAVVTDEDVSSVTNQLEVRIPMIIGVVVSMVFLLISFLCSLLL
PGVKSIRALINRNITGALFLTNLIYLVGVQQTSNLYWCMAVAILLQFLLISAFSWIFVDGVHLYRVLTER
RDINHGQARIYYLIGWVLPAIITALSIGLDAKSYGNVNFCWISFDDVLIWSLAGPVLLCCVIFLILLCLS
VNAFLTSKRTDMKREEILHDLRASFFVTPVIMGTWCLAIFAVNLNLTLHFYLFSLAVALEGGMIFFFHCF
TNKEVRLAMTKYRENCSRK

>ci0100132129
MSENETQCTLFNKDIYKCNVIIALKRTTGSLSLVGCCFMIGTIWLFRKYTILSQRLILYLSIAALFDAIG
YIIGDMTPDGTTCDFEAWWMTYFDWTVLMWVSCITFNLYMNVVKQRRTEKYEKFYHLLSWVCPPLLFSLL
PLIGDNYGPAGAWCWIKHTSTVWRFLIWYVPLFLLILAMFGGYSYIIYFLSSWQGTYDPDEERTVQLIKE
DIKVLKAYPFIFLILSIFPFILRIHNAFTAEGTDIFGLWVMTALTAPLQGAVNAVVFGLDPETRTKLTWA
QIQLAWASRFSHSAVHEYPTVFNNPLDSPTSGESVINPVNTEAVRRSSSFDQKQRYGSLNLEEN
>ci0100132133
YSEEQVVIFSALTSILCIIGLFGNLTVIVVIRRDRVIYKHRQNLYLLSLAVADVSLVVLVVPFSITNELL
GYWPFGTVYCRIYLSVDILLCTASIWNICMIGLDRYISVKYPMTYRKFRTMPKIRLFIVSIWLFAAVVSL
LPFVSEIETINSERGCFINASSWYIVVSCSLSFFIPSCIIWPVYIRIYMIGRDLEISKRARHSSKYLYII
CIINASLQYKLRLFKPFASFKPDGAVTKESTVYVRNPPDPVFCCCLCSQTEDKSNLIFFRPLKKESINGD
VTKSNFVKRLQSLSSVTTLSSSQSQQSSCTTAMSRRERRFVCIISIITGCFMACWMPFFLTYMIYAVCRA
CCINNTLFKVFFWLGYLNSALNPVLYTAFNKDFRSAFQRLFHLSGRRTI

>ci0100132380
FASREEMIKSPVLRYLVWIIGGNVTVLFETFYGLLWSDERFRNKRPFAKVNHVLVINLALADLLMGVYLL
LLGTEAAWRSGSYCKDDKSWRSSDKCGYLGALTVVSCLASVSILLLLTSYRLYGVFWPFRSQRVNYKTTM
LFAISAWLIALLLGKHCGDLCFFRGEFGYYSVHSVCMPKLYVTTKDVAWRHSMIIIFFNFFSILYILAAY
LLIALKSKKRVTKYGNSMSSRHSSMFRRITYLVISDIACWLPVCTMAFMNISGYKISENAYAVSAVVLLP
INSALNPLLYS

>ci0100132478
MSVSLQCRHCFTVDFFPFCEAITPKTHLLLAKQCVPCSSAFKAGVAATVNGGHTEYYDFYKITDRSLDPG
SYWQSWEALFTPPHAKFSFAIMFISQEASLCSILLLNIDRHIAIANGIRYANIITNKRCFIIVGAMWVGV
IAVGCLSTFLMSDYLLFRIKPSALFLPMPNPIKAKHYIHMMLLAVIPMSVVFLLNVGVTGYTYYKLNARS
ETVPYTNSGRHGKRKSTVASLSESTMLSAAESTVFMVLTDPDGNTIGVNENISSPTDGDNVFSSKRQRSG
CWGSDVSEIQVSSSKGDIQSVGQNNSFSETKLTLKPDNLSQTSPGVISNAHAPNRANDHRQDGSSLVTHR
LNSHTSSILPNYNSSPRQEQLYGSTSKLKRKRRKPQDLEHKQANRTLILILLIYTMCVLPLVIVLIINLF
DDDLLQGKVSDGISKLESRSIALTVTVGLFMTSSLWNCIIYNLRNRDYKRSAMSLLNGLRFRFVNICITT
PTSLH
>ci0100132620
MTTAETTTECYEKNPYIRNEMGWVPKHILIAERHIYTILAVYMTFIFLLAVSLNGFVIIATMKNKKLRQP
LNYIIINLSIADFLSGLVGGFIGMISNSAGYFYFGKTVCILEGYIVSVAGVCGLMSISVMAFERYFVVCK
PYGPFTLTNTHAALGIGFTWTWSVLWSTPGLIWLDGYVPEGLGTSCAPNWFSKNKSERIFIFVYFVFCFF
IPLLVIIICYGKIVLFLKQATRQSSASSNRQADNKVTKMVLVMISAFLICWTPYGVLSLYNLDYGLGAVP
VFFAKTANIYNPLIYIGLNKQFRDGVIKMVFRGRNPWAEEMSTQQRQRSTEAGQPIVSNEV
>ci0100132869
MLYSWASAAFLVCATLVANARAGNYLLQLNCSTTDSICDSLVSRIALQLQSSLVNITNTINSTEVPRDVL
INDFNECSQNKAVCSVNARCINLFRTYTCQCLPGFVDHNPAEPGTNCTDVNECADVTLNKCSSNGTCTNI
AGGYTCQCGNGFVDGNLQNPGTVCQDVDECQKRISRLCLMFSNSVCQNTIGGFECVCKPGFVDNNPSPIV
AQCVAVSTAQSTSSFATTDENTTSLVTTPSPSTSAPTTTTGSNASTNQVAITSPLSEASTTKVVTSAVSS
STSITSDGFTQSTAASTIAHSSPSSSAVPTTSNVSSAASVLRSTSETTQESSTTTLATATTTLTSTAPAT
SAESTSVQSEILSTADMSSFTPTSSVSLTTTSEQTSTDATTMVEVTTSISPTTSPAPSNPATTTSSTQLP
SSTISSVTSTTTEISTPDVSSAPETTSQPAIPGINVPLTQDNIADVANIFTQQVLTIRPDADMISPEEGT
ALVQSLITLVNPGENITVDQQVAEAVLAVVDLIGSSTTQFTDATSNNITLLVDDLLNKMVLNNGSFQSNL
GSLSVGARDEDGTVRLDLPPFSISGGFNTAATNVAQIKLPTNILSGSTQQERLSFTGYMDARLFRTAGTD
SVIRGSFTRSVKSTVINSAVLGSSVAGRTIQNLPNPVEVSLPLTLLSVPTEPQTNSTTVMTSFYNPRCVF
FDYQLNAGKGGWSQEGCVTSQSSSGAVTCQCNHLTSFAVIMSVETTVDDHILNIMTYVGCALSILGLTLT
IITYTIFKHLRKYKSKRILTHFCLALCGVYISFLAGIAAKSPTNEVQCITSGFFMHFFTAATFAWMMVEV

VDMYLMFVKVWSSVRHYVRKASVFGWGFPLVLSIATLGAHFGLVETYPNSEWHRMYPMYRETVVCWLSPL
AVYYAFLAPLGLILLVNTFLFVIVLYHVTCKRTGRKLTRVRSNAKRVKGRQHVLNAMAILLLTGPTWLIG
FLINVDPIEDGALRRILAYVFVILNAFQGLWIFIVFTVRPRVVRSSWVGLLFKHAGLDQSRSQTHSSGRR
GTRNIGSVVTTRTNTRRKSSLDVRRDSTAPFPPVSPESNRSTDTKSPLSTKSSSSSNPSISSGQKSPVQY
RYTKDIDVKLLKVTSLETNDISINNHNIAIDTTISTPTNNGSMYSSCESNGHSANSITSDVRTFM
>ci0100133026
RIIKMSSPNLNNVIATGCVIAYISLILMAIESKQVSPYIFDVLCKVRQFLPPCAFTLAFGTMFLKTWRVH
AIFTNVTVKKQVIKDYKLFGILSIFLFVDVIILITWFAVDPMRRVVKNSTNEIWKEGGQYVIIPQNEFCT
SDYTFIWMAIVCSYKGILLVFGLFLAWNTRHVALPTLNDSKYIGVSVYITVITCVIGPTTSFALKDYHDI
SYAITSVSILLCTTATLCLVFVPKVTALRRLGDRPDNSMQIRLHGVSI
>ci0100133065
LTFNTIQLTRVIITWILFLVSTCGNSFVLYCLCMRKQRLHVHVITMHLTLADLAFTFFSMPMDATWNTTM
AWLGSEFLCRLCQFLKQFGMYISSLMVVVIALDRVFSILSPMSANQQRKRTKILLISAWTLSLLCAIPAL
FLFSLIKKQFCPDQPIFYQCVDFSPNINKQDLKPYYFFTMCVSFLIPLFFTVISYSLILCEINAMQRRDE
RITGRRDNNIERARMKTLVLTSLVTLSFIVLWGPYYAMGIYHWFNPRERATFPKEISVGLFVLMYFHPAV
HPILYGFFMKDIRKHFLVTLMRCFK

>ci0100133186
IQIKGLFGNAMLLGIMKSVKSLSVTDHYILNLAISDVMLLTTLPLATADMITGNLWPLGWFACKYMVCVV
YFNISAGVWTVTTMTIDRFQCIVQANKMRGKRTKERAKKILAVVWCISLVYTLPVVYFADKRYEVDRYSI
GWSKIGHFLIIFFLLTMHMTFRFTVAFVVPLVILIICNTGVVCFLKSKDRLFSGKVRKLLPAIVTMVTIA
FLICWLPNFVTTLIFVFSPAVLGVIGPLNHRYYLLFHWMTVCLLYFNSCLNPFMYALLGGNYRNKY

>ci0100133368
MDIKLGIIILCCVISEVSTSNVHPQCRPVTLPMCLRNDNERNKGIKWIYNETTFPNFNNDASESEVEISL
SLLHPLLSTSCSRYLELFLCSVFSPACMGGGVVPPCRSLCEVVFDDCAEVVEAFGIIWPQRLHCDNFPPQ
NGGKMVDTGSDTWGKMVVSRSSWLVWGEALLGDGEVCIPLSGDDVITPALSQNNQEIDNVTKYPGEYGLR
CPAQLTAPPNEPGYTFLGMQGCGAPCPNMYMGDDELMLVRYIICVLASLCALVTSFVLFTFAIDTNRFRY
PERPIVFYAAAYFVISVIFLVGFALGSDVACKHEIMDENNIVIQGAVVVEGPRDRACTIVFMILYFFTVN
GTIWWLILTVTWLLAAGFKWGSEAIEKHHLYYHGLAWGIPAVQTMVVVTTGKIEGDNIAGVCFVGLYDSD
GLRFLLLLPMSFYLLVGMFVLLTGFICLNRVRKSLHDDETNKKKLAKFMLRIGVFSILYILPQLALIAIY
AIEDSNRKSWESAWFVRNCGRYGVPCPNLPHPVAQLAQGNPLPGVFIFCMKYIMTLIVALPPLFWVASKK
TMASWKEAVTKQRCMDDGNPLSVTQTQLLNDSNNGPHARDAVRKQQSKHSDVIFGGLGGFQGSSASIAAA
EAMTSSRARARQELWEDMTSTDSTLSSRSESTLAKVLEYESHKNKGRRVVGRRRHNQGHPLPLTHNPDEG
ENNREVDDVINKPVPIKMERNNRIDDVISSQSRSLSDVTRPQSSVTSQPGRPDVVKKQLSISRSDATRSV
PDIIKTTELSPPLLRNKPEIPRKPKLSLAKPNTSGRVLEV

>ci0100133550
MTTSFLTHTTLFTPTSPLAADASGNATLNTSINYTGNYSGETEYGAGQRNQAAALAAIIVPIFFGLIFCV
GVVGNSLVITVMMKMYRYQAQQGLNNTNRFILNLAVSDLFFLVFCVPFQATIYSLPSWPFGSFMCSFCEF
CQNVSMIASIFTLVALSFDRYFAVVYATSAKQLRTRTNANRGLIFIWVAALGIGTPSALTRVQVEYEGLT
LCVPNGHMKMWYMGYQLVSFLLGYLLPLLIILSCYMGVLHAMCKNAKSSALVRSKSARGSTKRVTRVVIM
VVIVFTIDWFPHHLVALWTNFGDFPYTSETFVLLILGHCLSYANSCLNPLVYAFVSNRFRADFKKAFTCQ
YFSEAVVHIRAYSRRFVQSFSVQLRHRSSSSSESGQRDKPLNPRPPVISETLYLKRSGRDEQETCTVMLT
PEHGRKEKKSPQTDVLLNGPTHQTTTFCYYDGQENKFQTQTEVVGLLNQNQQPTFIDNDDESDGMVL
>ci0100133606
VVPSIYIVICIVGLLGNGVVLGLLLRPDHSHIHGNKPSVTNILVINLALADFIFVGALPFWITERFLRGS
WIFGNFGCKFLSFISLLNLYGSVFFLVAMGIDRYLAVVFAVRARNYRTPRNAIRVCIGIWLFASIIAAQA
LICVNGDVSRCLWHLPGGDAYQIAYFVTRTALGFFLPVFVIIYCYSFIIISLRRRSRNMSASGVRITNQQ
QKVTRLILAVVSLFVVCWLPNHIVTVINALTLHDIELQYKLHLPVSSCTASLISTCLAYANSSINPVLYA
LMKEDVRL

>ci0100133766
MRLVILLYHLNCNTLVIFCLTEQARDVVPADFILSIVSDIGCILSAIGLALTIIIQLTNRERRKLRCTQI
FVNLCFNLMMAYILFLAGIEQAHNDRSCTVIAAFLHFFFLATWCWMAVYSYDLYKGLIEVFRDDKENFMK
RAYAFAYGFPIAVVAINVGITVGYVDALADTVTCTGVNDLAQWSYRADYACWITGYSLYMGFLLPVGIML
VFNVGVFVIVLRKVIWREHEISSSAAKVSTKQNLSITLALVATMGPTWIFGFVMLLSTDSVFYYVMSWFF

AIFVATQVCNILILFKFFINVTSFY
>ci0100133821
IAKTSVMRLPTIGLEKMQELNAFDTPYMIRFPTSDKLPSLHKAVLTYPFHCCALTNYGFGEIFQNDETNQ
DEELVDCAADDERTSPEIPQQIQPPVSSEGFVDSPTLVEPTVGDVEANYYHVEEYDYAYYNVFSTFLCQP
MARSMRFCLKSQPPAPIHCVPVPDLFNPCEDLLGSTGIVAVSWSVACLSVIGNFFVIFMLLAVACDRTRA
QQRHLSVPKFLILNLAIGDFFMGIYLFTLTAMDSRSSGNYYKFGVEWQTGGGCDVAGFIAIFASQLSVFT
LSVISLERWYAIRYAIQLDKRMTLRCGRIVMCVGWIVSIILAALPLMKVNSYRKTAICLPMDVQELSGKV
YVAVLLSLDICAFMVVVGSYLWMYAIVRSANGTGRSLRDLRKTDARVAKRMAVLVFTDFACFFPICFFSL
FALAGLPLLSMSTAKVLLITCFPFNACCNPFLYAILTK

>ci0100134145
LLLIPLILMVATGNVLVIISVWLDRRLRSSTNYFLTSLAVADLLVAVVVMPPSLAMIVNNYVWPFPPQLC
GVWTMLDVFFSTASILHLCLISLDRYVALSRPFSHRRSESSLVGIRIFIVWATAFVIAVPLPILGASDRD
NLFIGDMCAINVPEFAVFGSLVAFLLPLVIMFVMYTLTILALRRQAKLITNAMTQSSDETMNPNHGKYSS
VREAINQIQTLLGFGVVIQPDGVKPMTSHASTKRIYRSKNSTRRLSSSFKHRIMANINNEQRASKVIMTI
RGYDVTSTYLQVLGLIFVLFCLFWSPFFITNVVSHLCQTCNQQLMGQCMNWFVWVGYVSSGVNPCVYTLF
SRRFRQTFLNILRGRCLR

>ci0100134273
MPTTPSTLGYNATNILLGVTAAESQRQETAQMVLLICILAGACSLLTIVGNVVVIVSFFINKSLRNFSNY
LILSLAISDLTIGAFSMNVYTSYNVKNEWILGEVMCKAWLAVDYTASNASVMNLLVICVDRYLAITKPVK
YRKWCTPQRAAIAIAGVWILSFLMWCPAIVLWDLFSTDTKMPTNDCYIPFMKDNAWVTVATIVGAFYIPA
TAMCALYYQVISKLRKRRLGLRSLAIKDTLTMAVPLPVNTNNISRSDEKKSFSSNSPSAVDGQDKCDQHN
KQVVSPRDSRDNPSAAATLANTTTSLLMATTVENDLKHSDTAETNSTLPHEATMPLVTRRSASIAKANSF
SSIIRKNSGSKCFYREPQRVVRERRVTVLLKFILTCFIVLWLPYSLIILVTSFWPSCMVPIYIWNLSYWL
CYLNSTVNPFCYGFCNENFRQTFKVIITTKWWRKEVRKSLRLSRRTSNAPYRFSTMLHKKREDLPRAKMR
ERFKST
>ci0100134424
MFNGRFDCSDGSDECPSTLDTNAFSSRDEMIGSAALKGILWVIAPLALIGNLVVIWTTTKKILKKAKNQI
SEIHYFMVLNLAIADGLMGVYLIIIAIMSVQFSGKYCYMDKIWRSGQLCRWIGALLVVSTEASVMILLVM
GGFRFYTIFRPYGSQFSEKMGIYNKLLILLAWLLSAVLAIVPLILSDFDNLLWVSGVFFVDDTVSKTFTV
TVITFNFAAFIMMVLFYGLIWKLSSEERDKLLAANVPTGGKLNYKKCKLQARVTRLLFTDFTCWIPICIM
SYIHLSGIDISGNAYAICGIVLLPINSALNPLLYAGLLDKAWKKWKQCCKKDNTSQSGEGKNKKGFLERF
QLKNSRSNDYEINSEDRPSAEFAVTTLTTISGSPIVTKR
>ci0100134571
RIWVTWVIFFISLAGNLTVLVSVTVMRKTQSYSHCQLIMTHLSLANLAFTLFILPMDAIWNYTLEWLAGD
VMCRVMNSLKQFAMYISSAMIMVMGVDRVTGLLRPVSANQQRRRIVKFLTVAWVFSFINSIPPEVFLFQS
VMFSAGPYWPMRCECPNHYVVQCVDFHLIKKGREIFYIYSMFISFFIPLYCIIICYLIIAFSIAKMAKRA
KATELQSSGFRPPSSRKSLARRSLQRAKKISQLVTGLITITFVICWGPYYVVGLMHWFN

>ci0100134612
ALSVFADVGCAISIVGFALTILLHLINKKARQRTGIQIQLHVCFNLMVAYLLFMIGVPSVGNKTTCTIVA
VLLHYTFLTSWFWMGIYSNRLYNSLVKIFSKPANHYMVKSSCLAYGLPLIIVGINLAVTVGYLDKKISRP
LCPNADNMCWIHQETLYFGFLLIVGLILLMNCIIFTILVYKLIIKRNEVQSSAKKRSKQQNLVMAITMCL
SMGLTWVFGFLMLLSYDPGYQSVMSWLFTI

>ci0100135442
HAQQRVKSFKYRCVYRDTKRGVWSGEGCRTVSPESKHSVTCKWSHATMFSVILSVEFLTVSYNLQKFSIV
CESLSVASLMATVIILLLVRHNLHSDRTMVQINLSLSVLGLHLFALASTAATTSPAACEVVTVFIHFCLT
SSVCWMFVEAFLLYLKTAPGNLRFAAYSESRPGALHRVLYMFGLCFPLVLVIVAASVGFTYDDKYQRCWL
SVGSKMVLWAAVVPIALILTINLCILIRTTSLIWKLRQQALSYKPSVKYPSEQRRLSILNMAATARALFL
LLPILGIPWAAAFLVNAGQDTEVFMYINAGLNGLQGVFMLVVYCLAKQDVTNALKRRFQR

>ci0100135674
NRSCEFYDYNSTRWSNFGCKVVADGTNFTKCYCNHTTNFAVLLISGDVGKIRSKNCAYHSVLSIAGYICN
AVSVVFLLLVLVVYVRIRVPLFSNVKFVIHLNLCMALLGMNCVLLLGDLAARTGYELCVVVAFLTHYFEL
ASFFWMLVEGVYLHLCIVRGNIKNRTFGRMKFLFGWGGPLLISGIAMAFGMARRKYIYRSNYNIGLNRIC

WLHHEQNLVLSNTVPVMIILCLNTFFLVRVLYTIAKLSKNQSQTKDSISISLSNAFLKLFPLLGVTWLVG
WIGSLTYQRGRPLCDDSLTFGFILAHTVLNGVQGVGLFVVCVLNSTDVREIVTR

>ci0100136041
LVIVMTVGSIGNMLVVAAIVIRKKVHVYGNIFIINLAVADLMVVMVLVPSVLSNVIANENTLPDIPCRII
GFMMSVTCSCSIHNLTAVSVNRYWAVVRSSSYSRVFSNRNTVIFVIAIWLWSIILFVPSIPLTGKPYDPK
IMECLWDDQFSRLYTVVLVVVVIMFPLITICVCYWKLNVRKGGVWFKKQQESTRVRQPIKKNDNHVYRRE
IRLLKTLAVAILAYVICWLPYGMCIIIDPINIPALPKKLNFVLQIFGWMAFTNSSVNFVIYGVMNPSYRR
GYVEVI

>ci0100136241
MLKRLYNSLNFILFIFSKRVIGWDADMMNLTFNVDKCSSTKHWEFLVLLPFGFLSIAFNLTLVVAATFNR
RRLQRQSYVYACVTSTLLGNLLYVILHTWIILDNYLTKIEESGGDYTVTWTILQVSVASMLFVMCGNIGS
LLFVVLDSTYFVGRSVNRKASEFAKGFISDIESVFILGYLIVCGIRSFQQVMAPPTRTSSVMPVVKEENS
VAESDIAAQEEYTLSDVHNAMTSLGDSDEEIKEEEKKNDEKEEKNDVKKSMKRDRHENGGKEVEMAVNDV
DPRIVRSRPSFRSHSRLTFMIVISLTFVVCSFPTFVVFLVDTFANISNRKVARLTTMVMMYIYTFICPCL
LLKYLPNLKSALILLFTNCSVTRIPKRRSKRETTAR
>ci0100136887
NIIHITMNFNETNANLEFCNNTTPRSLSIAEAVACVISVFTVVENMIILLAIIKGPVSLRKPPYWFIASL
ASADLLSGIEVILAIFLPVGSSPLSRVALKGMAVVTFIASINSLLLVSFDRYLRIAEHAKYNRILSRKVI
ILLIAISWAVSFIMFLIVPLVGWSCLCICKEAESICGQCSQSFEPFTKSYIVTGVVYFLLAVVIMTGFYS
ALFRIVKKKTFERHKHSNHVSADPKFKKRELDLAKTLAITLGIFAFCWLPVVALFIADIVITKPVKLLQK
AFDYALVPTVINSLLNPIIYSIRLPKMRETAKRVVMCGLFSGSMLVQCSYIYYVLCIFIYTVHIVKRWDI
L
>ci0100137028
MACEGYPIVLRCPGTDVIQVVAAEYGRQTADTCATTPEKMRNTACYLPEAFDIMSSKCNNQTQCKLDVSD
EHFPDPCPGTFKYLHVTFSCVKYMFGCPGTLTGIASNVTMDTTMRAQKGAWTKDPIYNPHHVFYASPSQP
RLQKFENMEHFVNGNRAISEYDIHYQMSGTSFVVYDGNLFFVKASSANIVXRLPVANIVKYNLHTRIRHR
ETALPGHTDRSTSHRNGANSIELAVDESGLWAIYTNTHNADRIVVSKMNKDTLEIEKTWRTTYPKRTALH
TFMICGVLYVVGSRTGEVEYMYNTNAVRLLAKSRSFLNYNFTSLKYNPRDRLIYAWEATTSKAVYYKLHF
GLPDPVEGTLPNYHSSAPGFIIDYIWISIRDNWVGGVSHINYRLGDKPTTTTSTTTTTTTTTVRTTSTSE
QSPFGPSVAPSKLPHNSATLAPPVVPVASCPSVSRDGITWPVTSLGQTRTMTCPDVLNEQATWTCTTEEF
TMSAVWDVNGPDMNRCVTWTSSIEAQLLAGTIDPSSAAENLSRGLKMSSRNGEALRTDEVTNSARLMKTA
ATRAGSGTTPLTRTQVRQITEGVTNAADTLLDSSNSITWKEMESSKRYKAADNVIKSVESAGFVLADSMT
NNYGGFQGDTADLLLQATVVVASTSEDIVFPDPSRLQRSTVWYDTTDSVTMPAGDIVQQASNGVTKLAFA
LYNNLGSYMDVKLFEDGKEDTQDWNVDSRVVSASLGSPNREVKLSKPVKIVLETKQNIDAPRKCVYWKYS
ESLDKGVWSSKGCSVVESNTTHTTCSCNHLTSFAVLVNVHGTKVTSWGHEMALYVVSCVGISVSLVCLLI
CIYTFTFFRNLQSDRNTIHKHLCISLFVAELVFLVGINQTAHKVPCAVVAGLLHYFFLAAFMWMALEGFQ
LYVMLVEVFEGRSRWKWYYLTGYGIPAVIVAVSAAVDPSGYGTEHACWLRVDSGFIWSFVGPVCAVILTN
LIFLAITVYKMYRHTLTYTDSSKVNAVKSSVRGASVLLCLLGITWAFGILWVWEQAPVMAYLFCVFNAFQ
GLFIFIFHCLLQKKVRTEYSRCFGHGSCCSKGTSEAYVSNSMSFSKQYTPGRYSAAATTHNTTLTTQTAA
VSRIGXXQLRRMWNETIVNGPRTSDTVVTHSQDINTVPHRVHRNREAVPENSDEEAYQYDRLQRHCHPNC
THEHHTLHHHGSDVECAHHHHTGHHARHQEIARITKNGATKPPASAPQCPHPTHSITAHTATTVALSTYQ
SQCMVKLGADL
>ci0100137355
FLYSYWMCDDGECVHEHVVCRGGNDCGDGSDEKYCSGNCFPPFRKLPNFFTISQEQVCDTLIDCADGSDE
VNCSGSCWPESACNSDLTCKSGYFGCDNGKCIDDSNVCDMRRNCDDGTDEIRDWHGFKCVLGKHFETCVL
PQEQAFDTVVRCRDHADKCHTNGTCTCFTCLDQKLIISSKQQCDGIIDCYDIIQNSDSCACETGMFDCGD
GNCLTTNLVCDGTPQCPNQIDEIDCATHYMSTMKMTACPGDVNNNSLIQAVMCDGRPECFDLSDECDGSC
NVTAPFCNIRTMFNSRNGPILYFDVKAICDGKWSDCVPRIDSEELGCPARHYCQSGDSISIDKNLLCDGV
SQCDDKSDETNCTDRFFCENGTPFSVPMSKKMDGIADCTDMSDECPKELDENVFSSRKDLLGSAIFRATT
WIFGIAAIVGNSATFIYAAKILRNFKSLNPITKINYILLINLSVADGLMGIYLIILGAKGLQFSGNYCRF
DKSWRTSSQCSSLGSLALVSSEATIFILAFLATLRLCSILNPLKTKRTSTKFPTICIGVAWVLSVFLGTI
PRYPALSDYFSSAGYFGYFSDNSVCLPKFFVSPGEPGWGYSVIIILFNFFMFVYICVAYLVISKATSSPT
IRRHRSNQLQKMQMRVTRLVLTDFACWIPICIMSFLQLGGVEIPNQAYAFSAVILLPINSALNPLLYSDI
TYKKFKNFFASPKRRGKVVSVVQCRKSEQDRSSNEEDGAKASEQTEEQQF
>ci0100137512

QLKCVFWDFNTSSWSDKGCCLNLSAGLSHCQCNHLTNFGLLVKSTPVAGDVAVSIISNVGCGLSVIGLII
TVVVHSIGRELKKRRPARILCHICSNLAISLILVAVGIPQTQVVILCSIIAVILHYFMLVTWCWMTVYTY
DMYMSLVQVFSQRGKSFMIRAACYAYIIPAVIVAVSAGLNDPIYESSYRAKNMCWLHGPVLNYAFLLPLG
LMLLFNLIVFFVVLRELTWKQMKVVSSQKRTVKQMLTIAVTMSTILGISWVFGYFMLLSNDPVYVSVFTW
LFAVTTTLQ

>ci0100137752
MRGCVVWVQRKFNSKWDLSMVMHAYRVIICILFAIFITMGNILILAVIWRTPALHHSQSVYKFSIALSDF
LIGAIVFPASANSILMLVKTPYMLVSYLSFLGFFTTVGIAVSVYTLTAAGVDRLIAVYRPLRYRKDRAKM
FAKRICVVIWIVTIICGSLPLYVSSLRYSLIASFLVSSAGTGALILYIIAFALPVVAMWIISVATFHMTR
KHAMASSHLTSDSKKKSRSIETRLARTLSIMVGVFTLNLVPAAIVTIATFLTVSVYYNLPEYLDFFGTLV
LVCNSLWNFFIYSYRDNRFKKALKVLF

>ci0100137774
MYLSPCLVGSSAFIRDIPTTEDVITWKTCCFAGICCFDVGFTLMFGSLAAKTWRIDKIFQQNSRKQVVTD
EKLLCAVFLLVCLEIVILIMWHIHDQSVCYFVQVEYVSGVYRLTKLLENHLSLLNEDFVVSCATAYTIIW
VFCICAIKVSILGVTIWMAWCTKQLSKASFNDSNTHVVSSILALFVFCFSLPIVYLTPPNIQFIVFFTVV
NATTVFIVSIQFIPKVSLFYT
>ci0100137803
MSTFMHRSLSRQISNKQGSQCSCSRQAGERIEGHGKKFPFNITFISILICLLSATSAEAGNTTATIMQIN
FTTEELPNVTELFPNHENEVSGSNIVYKVGLSILLGSICVVTVLGNLMVIFTATIFRKLRTIPNMFIISL
ASADLVMGIIVLPMWSQYVIGDFKWGLGFFWCDVWTSIDVLSVTASIGTLCAISLDRFVAITMPFEYATK
MTRLRARFIIGFIWIVSATVAFVPINLGWWKTNKPEDWECYESATCCEFRPNMIYAVVSSCISFYIPLTI
MICAYSVVFKIAIQKRGEIRNRRGVFRRASMPGSKSLLWGKREHRAVFTMGIIMGTFVVCWLPFFIVNIA
QVFCQCIGATPFLMVNCLGYINSFFNPIIYCHSMEFRRAFKRVLMCGLCRDG
>ci0100137823
MDEFSTSGMSEINDNNSLLLNLNSSHENLTAMEPKLALFVCLITTSISLVTTAGNILVIIAFYMNKRLQT
ITNYFILSLAYADMVIGLVSMNLFTIYIVQGEWNLGPLVCDIWLCIDYVACNASVMNLLIISVDRYLALT
KPMTYRVKRTKRRACLMIVGAWTVSFILWVPSILLWPLVEGGRNVQNKKCYIRFILASPAVAIGTAMAAF
YVPVLVMSILSWYINRVNVEHRKYANTFRIRKSTNEKNIGQYSVNKTLNGTRSSAMSDRIKERNTQGVKV
WKMRRNQNNTLEEDEHVSVHLFTFKNIPFLLLYFGLHTRLERVFERMWIKFELQNKENGSKIQPPLAVIK
VETSSFDHLGQEPHKDEITIEKSKSSFRLSETTASLLNATRQNVRNMSARENKAARMLLCIMVVFIITWA
PYNILVVISTVCGEDGCVNDILWKLSYWLCYLNSTINPACYAMCSPEFRRTFHRLLRCQRSN

>ci0100137935
IALAILLAIIIIGSMFGNILVVIAVRSERNLQTTANFLICSLAITDFLVACLVMPFSALYEISGTWVFGD
ILCQTWTAIDIACCTASILHLCAIAFERHRSITSAVRYFSQGRRHTVAPKIVLVWVLAICISIPPILGWK
PSSQQQTQVNNVTTAASTVQRCEVGHYREYTLYATLGSFYIPLALLLTAYVRIYVRIHQHIRRSESRTRL
ESLSSEDALSLRSKRCPPGRIICESSRNKNLGNKKEMRVNGKNKVVRFVDQRIKPHQMTKGTSVAQENKG
TNVVKRTQDTLHYKRMPSFETETENLRGIFKCCKKYRHSESQRRLLNQRRSSIMLAHSLSLRAKVKLLKA
REVRAIKTLGTIVGAFVICWLPFFAVTLAAAFCNCKMPHTLTSIVLWLGYCNSLVNPILYGAFNRDFYAA
FKKLYTTHIYKIRCR
>ci0100138509
MCCYNLSNFQVFDDSEVLAYIAAEIIIAVFALFSNLFVFIAIIRFKNLQTPTFCLLASLSVADISVALLA
IPSAIVLRIGLGTVCFPLCLAMVAFQLFATQVSIWGLLLIAIDRFVAIHFHLRYRAYITLYRVKVGIVVS
WLGGSVIALGWESAVGASAENLHAVKRCAFEQVVSMEYMVYFNFVGCVLIPFIAMFALYLMIFRVNADML
NVTKACYRDSVAGWARRLRRKSKLLWKEFTAARSLFTVIGLFALCWLPVHIINCITLYCPDCRRPDWLMD
LAVLLSHANSMVNPVVYAFRLREMKGALRKLLVKYMKTLTCK
>ci0100138913
QVIAISIMAVVLMVVTVTGNFLVIASYRANRQLRTINNMFLVSLACSDLVIGVVSMTLYPIYMITRTWML
GPILCDIWLCIDYTLSMASVANLMLICLDRYFSVTRPFTYREGRTRGKTRIFITIAWVFSMLLWSPAIVI
WPLVRGRTVEDDQCQIQFFEDATITLVTAILAFYLPVLVMTVLYGLIYRETRKCSQYLEYLSSYGKSRST
SSASPFLNLRRRSYRGQTPNGSPKLGSFPLFSKAPGDGGNEPRSRLGSFGSVSLFQRRSSKGRSNNDKLE
SRIRSNSTAPRISLSPLAQEAKPDFAAKTEDSDFCEVFVPPPKPRSKSFVEKVRSRKHSTTESKSSQSSN
ATKTESVLERLKQQSSASEQDSLGARDRSSKVRLVAPKVQVSNHDDIEITCPRQRARSGGSVRMPSSERK
AARTLSAILLAFVVTWLPYNVCAVYKSFCAEGHECIPIAAWDAAYYLCYINSTVNPFCFALCNKTFRETF
IQLL

>ci0100138955
MATTPILPSDFIFFDQVNSTTVLPPGNYAANVSTDIDLGSSFNQTQGNICEPINNPPLIITAGIISMAVI
IGNGLVILAIIGGNSRFFRPMYWFIVHLSFSDLLVGLMLLWNYCLAGIFNINNTLTSLSFLFGIWVTSAC
CSVFGILLLAIDRYMQVVHKALHKLYFNQFTVGLSIFLSWLIPLLGFLVIPISLNLSCKDTCRCIEGKYM
ECKPVTICSQIIPPYTKNYVLAVAMYLFLIMPWPILIYSLIFIKARRSTQVLRQKLRTRDIRLIHTLVVI
MVVLIVTITPSGVILIIVVDYTFYIVVIATLNSLANPVLYIWRITAIRVSLQRRFWCFFSPPKHPGIESR
SSRIGKKTTDRFNSDVVSVDVSEANHRPTERFLFCGRTMFTSNVSDISQESTPDSQRGSAHKSPWSRNKH
GLSVELQPVLNPKPSTHSPLCQNVVVEEVWRENNSHLSSRSTFRTKRVSSGSNIDEELPLRPVQN
>ci0100139176
MTSSIPLLAMHRVRNGTALQRQIRITELYIIATVFILGLIGNSCVLVALWQRYSKHTRMHILIFHLAVAD
LIVVLFEMLPEWIIRFGFGFFASDAMCKFVKYMQILGMYGSTYVLLCAAFDRYRAIRYPMQSFQLTAKRV
HTSVLIAWGVSAFLSTPQLFLFEKPETRNMCRMKRFPSAAFDQAYTTWYFFVIMFIPTCFLIYLYGMIAV
LIMRNLKQKNKAGSSTSPVRQGGIFSSTTGNFAPRASSTAGISRAKIKTVQMTLVIVITYVVLWTPFFTV
QMLAAWGAINEDHIAASCIKLLASLNSCTNPWIYMAFSGNLLGDIKRFILCQKKKKSSFTNKSRQGDTGK
KTRNYSAFDPTTKETDMTATTNMELSVAKPKARNANNKKKYQTSELSDIASSGLMENESEN
>ci0100139179
PGLIIALYSVVFILAAVGNILVLVTLISNRRMRTVTNCFLISLSLSDLLQALVCMPISLIGQILKRFVFG
SVMCKVLPYFMGISVSVSTFTLLALAIERYNAICNPLKSRGWQTKSHAYKVIAIIWTISFLIMSPFLVFS
KLRYMPLVKQGKCTLACRLYFGGEQARQAWYVLQIVFLFCIPGLVMTVAYTCVCLKISEGFQFETKEKKP
STRVKGESNGNGNNQSDDGTNRLQVPDKKSNGRQLSVRKSNRKRCDSRSLQTEGQLLAKKRIVKMLIVIV
VLFFVCWTPLFIVNVWKAFDPKGARKIFSGPHEFTQLMSYISTCVNPVTYCFMNKKFREGFLKAFICCLP
NRCAAAVENQVK
>ci0100139268
CVYWDFDTEGWETAGCWLNYTSDKPVCLCSHLTNFALLVVVSYCIQTTIQVPSGTALSVVSDIGCVISIV
GLFLTIIAHLLHRDTRNRRPAKILLNICSNLLIAYFIFVVGVSQTSNRTGCKVVAVLLHYFFLTTWCWMS
VYSYDMYMSLVKVFHKSQHQFLQRTSLFAYVVPFVIVVISASVTMGYLDLQNEDSFKSSSYISDHMCWLR
GNSLYFSFLLPVGMMLIFNIFVFVSVSRELALKNKSATSPNLKRSTKQSLTIAITMTSLMGLTWVLGYFI
LISTDVVYVTVFSWLFALFNTLQGFFIFYLTSVRRSDMRSVWLR

>ci0100139465
MAKEFQSEDFVSVQNITKSLEELESAAAEFAFSITNESASAIYGEVEIGVTVSNSSSKSCFRNSTSHEDC
LLGFSKVAIPNIVVENGQLGKNSTSILVATQFRLPNVILEKLEPKYEEKGTELFQTQKFVDHTGSQLSAS
VYDKATRKKLRSSLTYLMKTKGFTSGHEIGKRRVSSITYSCEYYDVIRQQFSSTGCRLMTSDDEDVTCFC
NHTTIFAVLLSVTTFQPPLAVKVISYVTESLSVVCLIFTLVVLVQVRKQLRSERTVVQINLTASLLLLHL
LNIIHELAYLHTVSCTALTFALHFFLLSSGAWMFLEAFTLAMMTSHKALYFDSVSRKKLYIFRVFLGWGA
PFMVAMVTFAIGMSTGNYFDRCWLTNKGGVLLGAAVIPLALICVVNFAVAVKVMPVNLNISKLTRIDMTH
VKAALKGFIFLFPVLGLPWILGFLTGIDSYEASVAFMYLNVVLNGLQGVFLMLIYCVIGSDVRKAWLRKI
SCVLRTEVGSSTETSGGVVVTAQTSLPRT
>ci0100139945
CKGEWDKITCWPNSAPGRTVRLPCPEYIIDFDHTGHALRHCSRDGRWAMVRDTNRTFSDYGSCNIPREED
VVEMIRRGGDLYTVGYSFSLVALVFAMIILAYFKRLHCTRNYIHMHLFASFILRAVVIFVKDRVLYYGAG
ILDINTPDGEMTLEALKNRVDEIDADRSSYLVGCKLVMTLFHYFVATNYYWILVEALYLHSLIFVAFFSD
KKYLWRFSVTGWGVPILFVVPWAIVRAKLDDTACWDIAVTEYKWIYNGPIVVANVINFLLFLNIIRVLWY
KMRERGPIGKTDNRRQYKKLAKSTLVLIPMFGVHAIVFIGMPDDISSGTWWDIRMSFDLFFNSFQGFFVA
IIYCFCNGEVQAEFRKAWERFNLSVEIKRGRRERSRSSVTMLTSFNSSASQQVRIMTS
>ci0100140006
YNYTTFPNILGHDNADDASDVLYEYKLLLDTHCSTHLRFFLCSLHFPMCTPKVEVAIPGCRSMCEKVRAD
CEPVMRVGGVQWPDTIDCSKLPMRNEKKRLCMVPPGESPNGEAPKVDTNPQPPSVTLDESGAGPHIELEI
DDEDENDKAAKYWAQQLELNPGYEEQQWREPIIPRIGKMKQCVPRCEKTTDILFSSNHKQIAYWLLFSFS
LLCFISTVMTVCTFMIDSKRFRYPERPIIFLSICYCVYSVAYLVRSVNGPLNSMACMEDITHPAGASYFV
KGGIQGTGCTAMFFVLYYFGMAASAWWVVLTLSWLLSAGFKWGHEAIEAYGFYFHLAAWLLPAGQTIVVL
ILGKVDGDELTGVCYVGNLEPESLMYFVIIPLTFYLITGTVALVFGFGYLFKIRRVIRHQGGKSAGKLEK
LMWRIGVFSVLYTVPAACVVAVNFYQYQNLGVWMNQIPRVNLDCTLPDSIPSLPIFGIKIFMSLIAGITS
GMWIWSGKTVESWRKFYK

>ci0100140016

NTSTSEDRYLVNSYVISASVQPAPVELLKTQKVKFLLKHKNTSVHCTMRCVFWKPKLAVVGNSSAGHWST
EGCKRIRSNNTHTECECDHLTNFAILMDVRGVQLDKINNEVLTYITWIGCSMSIVCLVMCVFCFNTLRGL
RSIRTSIHKNLCFTLGLAQTAFLLGADKTSYPLLCPIIAGVLHYLFLTVFAWMCIEGMHLYVSLVKVFEI
DKSSRLACYYAFAYGSPILVVAITAAIRYDGYGTTQSCWLHAKDDLMIWSFVGPALCVICVNIYFFFIAM
RVMRSHRITTPAHRSRLAKTKTWIKGSSVLLCLLGITWIFGVFFVDSKSVVMAYLFTTCNALQGVFIFIF
HCLLNERVRTELAKYARRRNLCPSWVRGRYHVTNSVTRSNYNTTTSMGYRSRKSSSGNTENSSQTNGSWS
WFKSKETQNDSVIKRSVVRTITPKWKSDSKRVAGELQFTTELKKSSSSENKITSTTTERTESVVEVQKHE
PIQHNENVVALKNKLTPMLNRKVVVTVSDESTSCSDGADVDFGCLNAAFEGEATVTPLPERKERIERFYR
DYAEHFSGQGNKAESGIFRNNSMPVKVRRDTPQPRAPATENGVSAKSLYFSVGLSLKVKSTTEKVADL
>ci0100140366
MDPDDQSLETSSSFSPTEVTFTTVSPVHYEAPPRLNIVEQIVVPVLYCIISFIGLIGNGIVLYIMRHMVK
NKSVTDVYVTNLAVADILLLGTLPFWAAEHVMGRWVFGTIMCKIASCMTYMNMYASIFFLAIMSLDRWLA
IVKAIKCRTLRTVQKAWIISSLIWGLSLFFMILPSLFRTTKQYRHGDRCVWEFPKGMTYHLNVLYNVLRS
IVGFFLPLIVIVFCYSDLVRFMRKRSKIRKKSSDSAGSLLTQMVLVVVSCFIISWLPNQVTNFVHLLKLR
VCTCTTGYQFRTFA
>ci0100140881
MSNLSFCPEDGNTTSAEGDAMLTTALIAALTSLLSLLIVVGNGLIIVSVALVKKLRQPANYLIVSLALSD
FLVGLVVLPLTIVYDIMGEWVFGPNVCDVHVSFDVICCTASIMNLCMISIDRYLMITQPMTYPKRRTGKL
MLLLIATAWVLSCLVIIPALFGFTKNVKDEADWLPLNNDSLVGNETFALAGARNPYLAIDGNGTTQWVPG
NNELSEWHFTVDLGVIHQLDIVSIENAKGSKQHDLQYSLLTSPCRESTAWTSKATFAVESGPSRQETRLP
TEVEARYLRFAINQTAGYENPMLGEFQVFGIKKYGKACLISQERWFTIYSTLGAFYLPLAVMLCMYWKIY
LEASRFNARHRLRSYSTTGSQEWSGGSPPSTPDVTNNRRHEQFYERKRTYSSNNNYTNDKFHSTKSEGYV
TDSSEVLLANTAVTPIVVTDLDLDKAKDSVFEDDTVSSSGDEFLPSDRLEKVEPKTNGNGVVLNGHVIAK
IAIENDRKTNSLPCRGLVHRNGSAVSLPCKDSTNGNAVNLIRSSSGVVCGLGMKNGFPRNGTTRHNGMEW
RFPTISEVDILTSLNEEGEQTNKETETETINGKINEEYRARTLYTRASANSLLATEKTPKLVGKPSPRSR
LFMLYGKPRLLRATSTPCPTTTNKPNTQHTRKSSFRTLRQNSEITFPCNRRPTVFNQIRRRVSLATSRDR
MRNVKATRTLGIVVGAFTFCWLPFFIVTFLRPFACPIPESQDCIPLWLVRFVLWLGYLNSALNPLIYIGF
SPDLRETFRFLICCKCTNVDRRLAQIELRQAIAVERKASMASRSYAPESIV
>ci0100141310
EILTRYVTVGHALSLATLLVALALLACLKNIHCTRIYIHMNLMAAFMFRGLIWILHSAAFRGEQDLMVEI
LNNLVTLQNWCRLYNVFQIYFVTANYFWLLVEGLYLRLLLRAVLFSHTKYMKAFLVLGWGLPWLPVVAYF
VPKSLDPEENRDCWYEDRNSAYWWIVKVPILISLLINFIIFVNVVCVVGAKLRVNQRQQADDRREDYKWR
LTKSTLSLIPLLGTQYLITAFVHFDSEENPTAEFVKKAFEVSFVSIQGLLVAFIYCFFNGEVQEECGKTW
RRWKTRWELERRERKHHRMQRRRSMNHSSITNLFTSVVTRKSSLYTGES
>ci0100141384
MSDVGVTLSAEKTTSPTTVDFVGVVVDDNTFVVLRTIVLLFLLVLSLIGNSLVVLSVAQSRNNRHNPFNA
FILSLASYGLLECALTISLATGFSIEETWNFGDFLTGFNASFVQLQNTGIFLTISAMAIDRFLAVTRLTK
YHTHSSVHHANYAVLYTWIQSFIFALPLLFQNRMLGVTARPFVERCLCGLVEGTSVAFVVLLLILCFIIP
LLITIVFLHLVNKRAKYERKRIRTSSTPHYTVYCLQESMLLKEARSAKFVSLLLFLFMAFKAPYVILDTL
TQLNVGGAFYPANNSTIKVDGSKFYFQTVLSWMMFCFSSLYPVVTFIYFKEYWKRIKNWILCSNATSIIN
NGHLRSNPRHRRRQRRRLNENKRKKINPKSSIKHATAPSSVSSNIPSEKVTINGTSSGDNVLFRVPVLYA

ATDGLHLVASGKNNNNNNSPTQLPTGASEPHQVVDEVEDVRPSSVVDKRVDVRCSRDCLRDDVIDDDVIY
ITSDFDEDETESELNNSTNSRTLIWPHRHSSDTMWNSFVSRFSEGDPGIPPDEGISHPHTPTPDDVTNQL
TVTNDCDVRGSHGNKIHPNISRPFVNDSLEDAHKDIVATTKHKSSRSKISPFGQQHAT
>ci0100141547
LQQACRAEDGFFDCGNGECISRRLVKDTAQNCRNNADEIEAPLSNCTNSELRCQSERNSTKCIPRDWVMD
GVPDCGRGTDELTALVCDQEKEFQCVWNGRCIPRYRVNDDYKDCEDGSDEAINITCLNTDYQCENSLRCI
PRSYLCDGLDHCGDCSDEIEECEVPVISPCSKCNEEEVLCDGICLPYSKVCNGIVDCHISSIDERCCDGI
TECRNMEDECSERANCENKPKVCTDIPIIWKCDAVYPPDCLDSSDEINCSDSTHFYCESGTPFFVRRDQV
VDGKRDCADASDECPPDMFQTNALSSREELIKVPFLRAMVWFMAFVAIIGNCVVFILSSKSLIKNSIKAQ
NARAPGVVVINKLLILNLSIADLLMGIALLIIGIKSAQFSGQYCWRDLLWRSSTTCDVIGILSVLSCEAS
VLSLVCLTSYRVYMIYFPFKSTTVSTKVASIWIMCIWVVCFILALLPLVDQLSLGYFGYYSASAVCLPRY
FKLPTDQFEINAISPVIMSFNFIALLYICGAYAAIYKRTYSGNMAVNTAQRQHTEQRQKIAKKMQRKISI
LILTDLCCWLPVCLMTFISLRKAGLHDSVYSFSAIILLPINSSLNPIIYTDAVPKLYTKLKQIGLQVRTL
VNNTQTQITSTTGIPNVTTTSV
>ci0100141557

GSFCGAIWDGYACWPPTQSSKEAVQNCPSYLPRFNTEEFAVRPCQANGEWFRYPTTQYQWTNYSRCQLQK
GQEAVWAKYRLAISGYSVSMITLLVALFIFFHFRSLQCQRVTMHKHLFVSYILNALASVLCLFKFIHTLH
KCSVNTLLKDFFFQFDLFIYNIFSSPFIXQVYCKFLHAIHQYTETSNYFWMLCEGIYLHTLVVVSVFREK
QNLLIYTGLGWGFPALSLLLYVVTRFVLDDSSCWATPTTDAAYIIHGPIAIALVINFIILMNLLRVLLSK
LRANRNNIQRYIGAIKATLVLIPLLGSQHILLTIALYIPNASVLRVLSYITNVLSSFQGFAVAIIFCFCH
EEV

>ci0100141702
VAFSGVICAVGLVGNFLVMFVVLVLREFRKSVTHWYVLQLAIADSLFLISLPFKMIEDINGYWMYPEWMC
KGKETLLFLNYYTSVSFLMIMSIDRYIAVCHPFSDTLQRLRKPKSAIIITVTTWVAGLLICIPVMLYSFK
VGIQPNCRCTDTFTDGTPESCHYHTRAYGFKVFNIFNFVVMFILPLAVMGACYGLIIFRLTSGPLKDKSE
SRSGTNKSSKSEKDRRRVTIMCLCLVGCFFVCWLPFHAVHIAKIVGISGQTDALCRILPVVASLLAYSNS
ALNPYLYSFLGGNF

>ci0100141745
LFDCGNGQCIPRRLLRNLGRDCDNNADEEADASLPACANTEIRCGFDGNSTKCIPREWVMDGVDDCYQGT
DEQSALVCDQRKEFRCPGNGRCVARYRVNDGYEDCDDGSDEETNMICLDTEFDCLGINQPPLSPERRCIP
KTWQLNDRTDCTDGSDESTVPLNGSCPDGSFQCKVSLRCIPVSYLCDGLNHCGDCTDEIEECEEPVMFRC
PNDTLHRTCLHWSYACDPYADCPNVEDDIFTVGPGLCMDGTTVIAPRQICDGIFDCSDLSDECLCVTTSC
SQCRTGEFLCDDVCLPSSKVCDGVVDCHLSLMDERWCTKESVNCSESTHFYCESGTPFFVRRDQVTDGNR
DCADASDECPPNVFQISSREELIKSPFLRAMIWFMACVAINGNALVFFFSLKSLWENSKKAKVLQAPRVA
VINKLLILNLSIADFLMGVALLIIAIKSAQFSGQYCWRDLLWRSSTTCDVIGVLSVLSCEASVLSLVCLT
SYRLYTIYFPLKSRTVSTKVATIWIIGIWVLCFVLALLPLSDHLSRYMVTTIRIKTTPYLKTEMIRAVCL
PRYFKLTTDQLGLNEISAVIMSFNFITLLYISAAYAAIYKRTSSGAFSANTANRQQTEQRQKNAKNLQRK
ISILILTDFSCWLPVCLMTFISYRESGLPSFVYSVSAIILLPINSSLNPIIYTDAVPKLYRMLKQIRVQV
RAWLN
>ci0100141751
SISEADEILNDALGPCAPLAFDNLPNSYVTILIVLYSIIMTVGILGNLCVIVVISSSRKLWKVVNFLLLS
LACSDLLLALTLPWTIIHNTSYDYTMGSGLCKAIPPLQGTAVMSSINCLVAIALERHQAIVYPTRQNIFR
SHLRLMIAVALIWICSFCFQIPQIVVLDDVVWKQKTPVKPATVWYEITVHTCQETWAPQQSQAYSIAIIV
IVYGVPLALLVLLYGRIGYFVFSRRGIENSLSAERSERSRSRRIRLNVMLVLLVTSFALLWGPYFVFQCL
TVCLTSYCSPELVGYLQLVGHVNVALDPLLYAALHERVREEF

>ci0100141764
MSFEIKIVVLLLSACCTLINLFALVCIVSNFKRLRQRMYIFIINLTIVDVIVGVAYMIDVVTKILRSEAS
VTSQSEGFGATAATEGLKIASILLSALNTSVVTYALHKRTTSMGRHRAESLNNDTTGNPLHIPAFDTMLR
VLSLGTQTPSANRKGSRLNQSSPRKQPSVNGNSPKHINGDCLECEEEETSLAKKNESDRRIGQQRLSPGD
KQESSSKSRRKKSMSRLTAMLLPRTSLVSIRPSNFPRSMTSSTYSQSYVGRHGNSVVSVRTTVAILLVIW
IVFLITWLLPVATRAVMKASGSSNQVAEIFSCSLECETEYFPNIEQTNNDEKNQSPFSDSNDYDTGVMAS
NLSTGYVKSEENSTGLHPNPIRRIRHTKSRIRRYKDALFNESGFVAHSLLTVTSESNYTDVNSSGDFPIP
WEQRPMCPESCSNLFYPISKLHVIFHALVITVTWLFMLIISLRLLWLRKHSKNVFVRCSALWRFILYTAV
FYLVAYATYVAVCVLDAMHTNTPNDANRATSIMMLNVTMNYDRTNRSMTSQPANLVPPHGFEPSLLLLEV
ASCLVLFHSTIAPSLYMVRLLGFRRLLAGIRFR
>ci0100142029
DPISSRDEMIKLPWLRWLLWMVAIVSILGNTVVMVTTFWRELSHRGLPQIAPLSKCNDVLVTNLAAADVI
TGIYLLIIGFKSSSFSGSYCQHKVSWQSSSTCRRLGVLIMTSCEASTGIMVIMTTYRLYAVIKPWKVGHI
RPLVTALWMGMVWVLSFVIAVIPNIELVGNIGYFGKHGVCLPTFFPTVKDGMTGRAKIYTGFVMGVNFVA
FIYIACVYLMVWAKSSKLGKRRGAGGNEKLKSPRTVAEKRLLSMHKRIARLVLSDFCCWIPACIVSFCGI
SGVDLPQWVYPFTAVVLLPINSALNPLLYS

>ci0100142069
MIGNSIFRVGFWVIGVLSLLGNFFVIVSSIFRLRKAEALKTSFLWFVINLSLSDCLMGVYLIAISSKGAE
YSGKYCYHDAEWRSSNLCSGFGSLTIISSEVSALTMALMATFRMMSVYKPFEMANIRKRTFIIPTIFAWL
LGVAIGALPLIKTDSGYFVSSICICMPTLFVTVGETSWEYSISLISFNFVLFVYLVVVYIAIFKRSTKMK
SSAQRSNKLQKTISLLILTNFCCWIPICIMAFVSLSGVQLDRIVYVISAGVLLPINSVINPIIYSDVGRY
VVEKLRKKQTPPNQKKGQSSSAFTSTTIRSVTPINRTTSCI
>ci0100142144

MYKGFNYLHITGLNIFTVVVYHFVKTLNWTAAELVQYQASVVNTAPAVIGAVVFVFTVTSSLYHLLLLSC
ERLYAIQWPISYKLQSRSSLHRVLIGVWIVSAVTASVPGWFPKRFVFNYFPSLFVYYPYINLVDARKNID
SVIVLMGIFIVLPFVLTVKLFPIVALSIAYFTLRKSCAEIVAPTAACFYLSMGNSLVNVVVYSLFDKDFR
KCVKSNLRLRCRLC
>ci0100142353
MFFAQHWCDGKKHCEDGSDEVYHLPGFECKPRRGIQPNIASCVLPQRNLYTSLPFCEYNQNLCALEGGRC
FQCFDGMYINSASQVCDGEIDCYDLTDECLCENKTICNDVLGDSRERCGVNKLLCYGECVDMDKAVICNT
SFACEGDRNSKYCAAYLPSNETDFKCPKGVKVKACDGISECLNREDECDRTCANQTHFCSVDIACHKKLF
FLVDNRYCDGKPSNIKGCPFGFDETNCTNRFYCTNDSLENISKKRVCNGWIDCSNGEDEAMKRCNSTRFY
CLNKGVPLSVGIPRVENGIKECSDGSDECPPNSNRTSLFSNAFEMIGNPFLLAMFWIMGIFAIFGNIVMF
IGSVKKFKASTKPLTSCSLWFVINLSIAGFLMGIYLIAIAIKAAEFSKLYCYFDTDWRTSDTCDFLGALV
LISCEATAFMMTITTTFRLITILKPFKARSVKPAWSWVATIAAWCFAIILGITPTFNTSSGSFDYLKIKG
EFGYFGETSVCLPRLYEKVGHPAWEYTTFLITLNTCLFIYIVACYVILYKQRTKRMHVVRKDGGKGKKSL
QQRIFVLVLAHFCCWIPICILAYISQAGANIRNDAYILTAGFLLPINSALNPLLYSNILTNFVKTIYRKL
KGSDWCSFAVRNNDEEAR
>ci0100142526
VGIIANTFVIVVISIWERCHRNRTVANSFVVNLAIADLLFLVVLPLYMPAILTDGWIYGVGMCKVIEAIK
YINFRASILFLTMMSVDRYLGIVFSMRSREYRTRKNAIMTCIVLWLLSIAAAVPILVFANVDTVKKECVL
QFPGYESDFVSDKDLYDIYGNFALFFVIPILCIIFCYSAIIKAVMTRHIQSSTKAQARMVALLVGVFILS
WGPFQTWKLLTL

>ci0100142971
TRPSCQYIDETTNQWSPYGCKVVGKGQGSVHCFCNHTTKFSVLFVVRPLPFEMPPHHASNILVLEFVMEG
LSFTCLTITMLSLFLVLRRRSKSSTSTAARERTIVHINFIAALAALHLVRLISEASLGNNGMCNMTVVLS
HYFVLASGIWMLIEGLVLYLNLVVKSMKFNRLGWYKFAAGWGIPFVIVFISAGEGMYNNFTNTYCWLGTK
HNMIWSATGPLIAIFIINFFILSHTVALILKLSLKSKAMKPSSSKSNHGQHLHTTKGILVLFPILGVPWL
IGFLVNIREYHVNIVFQYIHVSLNGLQGVFIFIFYCAVNKE

>ci0100143048
SMEGDIMLAGLFPIHQNEGCLDLEDMGSIQRMEAMAYAVQKVNSWPNFMKNFTLGFRIFDTCNSQSIALS
RTLGVLPHCNNCTHYPVCFPECPSLVAGIVGPESSSNALLSAHACNLALTPIISYFATSLELTDKTIFPT
LFTTVPSDDFQVSAMLELLEYFGWTYFSFIHTNDSYYRYGKDALDYVQTNMKEMCLASVQQVSGDGSNSS
YDEVVTTLLRYKDQSQTSVVIVFTDGVSANKLFCAVDRANATGEFVWIGSEGWGVSLNDISDNLHVAQGS
LTFTPHTEIDPEFDEYFQNLNVTHNNPWWKEIWKDHFNWYLDIMCYRCPILPHPTIQMISCVIFLENSCW
NYKHIDETNGYLPDSTVSLVNDAVYAFAYAISDYVNHTDDIISENGDVIVPPWKLINYIRKISFPGSAQR
REITLGKSIAYYDLNNLQINENGTFDYVRVGSWDSVDGLILDASAIQWPDHRKTPISYCSEFCHPGEVQV
LIKIACCWDCERCAQGSIVVNNTRCQECQDFYWPNSNYSRCVPLELEYLHWYEAPAVLLSVFACVVLLCA
VFTSIYYIRHRHDRLIMASGRELMSVIMFGVIIQCVMVFFIIAKPTEHTCFIYRFIFGLDLTISYAAIVV
KSNRIFRIFTHGLKSVAPPRWTKTHHQLLITGCIVLIQIIVVSSLCLIFPPKVVKYQAKPGLAELACSLD
NNVTILLPLSYNFLLIVPCLYYGWKTRNLPANFNESRFIAFTVSTTLVVWIAFIPTYLTVDTSSKIHVEI
LATVEIMSAANTLACLFLPKIYALKF

>ci0100143057
NRYILQLAIADTLFLLMLPFTASSELSGEWSYGIVMCKIKEAILFINYYASIYFLVIMSFDRYLAVTKAF
SSSDLVVALRGPRAAGIITAIGWVISVGISSPLFIYSTVGKCNVCAYKFPLTKVENSTMVSYVANLFFCF
PLLAMCCFYGLIIRAIVTTQAGAGRNEKYRNRVTVIVLALICLFLVSWLPWYTVQLALMNGISLSNSECK
RLTYAVRLIAYLNSTLNPYFYG

>ci0100143146
MNTTAILPGAANSTQTVFNRLEACLLTVSCEHDDLSISAVIVAFAVLALLENCALAAAILTWKLGHACKM
FWFLWHLSVADGMLAVTFVFFHTMVILSCPNHFSCQSNSSVQSTVVFVMFFANILSSHLFVLAATLDRFI
AIKHPLRYSELMTSRRVRIVSLIIWVVSFLIPIGGGLGLNFGNSSIAYSVTTLTAAVISLTAAVVVIALN
IWMVKFAISIIMSMQTPPPSTQHQPSRPSIVPNIGIINESLKPATTLALFVVSFIVSCAPWSVALIVCEE
VKKCTIKQHQDYTLVLVLFHAIVIPFIYGFRVEEIRHRVIIFWKRVFDRAPHPAGVYR
>ci0100143219
MRTGYLVKGTFEEWQKCNNYNDCGDWSDEKNCTETCMDRNMTTCDCHLTGDCPVQGLLYHDPCYSKIQTC
NGWNDCGDWSDEKNCNYTCNSNQIACECNSNGSGCLPAKEGYISHCFTENEICNSKPVCTDWTDEKNCSC

RNSEFNCGCILGNCTSRNGCVSKENILNGKFECADKSDEPYRCFVDGKLNCFQCHDDQKMLISTKQVCDG
VIDCYDLSDECYCSNRTVCYAVLGNSRSECPIGRLNIDDDQVCDGKLDCEDGSDETESLCSETRFYCLNK
RPLSVPMSDVENGIKDYLVGGDFLRAWLWILGIFSLVANFIVFGTTVYQLKSWHLEPLKKAFTFFIFNIA
VSDLFMSVYLLGISIKGVQYSGRYCYHDVEWRCSVACSILGAFSIISSTVSALLLALMATFRLVSVYNPI
KMGSVKTTTFVMPVVFIWILGLVLAFLPLVPLNSEYGYFGESSVCLPDLFLKVGSNAWEYSAFLLTLNFT
LFLYMFCSYMAIIKRSRRMKSENKKNKKMQTTVALLVFTNFCCVVPISIISYITLAGVSYNPVLYKVSAG
FLLPINSSVDPIIYSNEVVNAAKKIYNHLRKTVRDIRSD
>ci0100143261
YVLQLAIADSLFLAMLPFKASESLSGSWNLPHFLCHLQQAVFMLNYYAGIFFLTVMSFDRYAAIVHPVSA
PWRRLRTHGNAVLITLAVWALAIAVSIPLFVWSDVKHCKCTYMFPRSAEEFQQHHGIWAGMHFVFAFLLP
FIVMTVCYSLITWRVFHPAVSSANRYNNEKDVLILLCFTQVSLVLMFLLCWLPYNIVLLAKFAELPYSGE
TCTLIENVTIVLVYLNSMINPVLYTFL

>ci0100143300
HWFIVQLAVADFFVGLIVLWIGTFSSLFLDTVSLMSGIATYGVLAAATSTSTLGVLFIAVDRHFYILRHR
RYKQIMTRLRVGTAIVVACVVPATFFVVVPAFGWNCIQSCDCRLYNIDQNRRYCFGSHCSQMMTPFRGET
VLGGGICLLLLLVVSVSVYVKIFVQVRFLTKTTGRQRNRRNSEMQMIKTMLIVLSGFVFTTGPLAILCVV
SYFHNVRELHNTMRILVVISTINSILNPIFYFWRIPDLNSNLRIMIRDSLIFCCPSCFADDN

>ci0100143330
MRDLALAVIYFTVSFFGIIGNVIVLYVLLRQRRGWSVTTTYLFNLALSDLLFLCIMPFWGHQYLNELNWA
FGLGWCKVVGSVTSINMYASVFFLTAMSVDRYMAVVHATSVNVVRNSCIARWVCISVWSAALLLSLPRVL
YQTLQPIYLSGPSTNTTLAGSPGTKWIKMGTIQFIGAMIGFIVPMIVISICYARIVVTVKKKVISKKVRK
DRVAKLAALVVLAFFFCWLPMQIMQLFSALGGWWKIKAFNFDKNLFHAVYPFMIALAYSNSCINPIVYAF
TTTNFQENIKDICGSDKASRPYKMTLSPQPGKNGDGKTGYATKTEAINMYSPCAPRSNHQYTPAVQVHAE
QNSPGDLRNQNHLENEKQNGAETFESQSFSYYSCPAVMQGLGKINCEQSGADSVAIHSTPLDNDAYDCIA
TDAV
>ci0100143362
RIMIIVVYMFVFVFGTIGNACVIVVMMIVPKFKTPTDIFILNLSFADLLFSSSLIFWAVEQIYEMVWFLG
VFMCKALSAVTLINLHASVLFLAAMAVNRYEFNMLILCKTVRRKRQSSSYGDIIKAKVSCVIIWLVAAII
SSLAWFRKTEIVPSPAGNVTRCLWRMPDTGDRYVWERIYFGSRVLMGFTLPLIVIIISYVLVVMRLRHTR
QQVTVKSSMQMRVTITEQEQLNKTLFFPVFAVIAAFVICWLPNHVTSIIHALTLGENEEKRKDLHTYELN
LISNCLVAFNSCINPILYAWLNKRFR

>ci0100143568
MNSRALTSTVLTAGRKFSEANYSVGINSTAVSINQLDFDIWEYYLKPTNAEWFVMSLYVLVFLISIIGNC
LTIAFILRRKHLRTTINYFMLNLALADIMVTIICLPPTLMVDFMESWLVGQFLCKFTPYLQMAVTSVSSL
SLGAIAVNRWFVVCHPLKVARTRRSAKHALLTMTSIWLFSLITLCPIIFVTELTEDFPGYKELNLLKSCG
EHWTTFLHQAVFHIYYVTVCYALPLMVMAIAYTNVFRKLSYTKIPGHVSRETNPIPKRRGQCHSCSSNSE
HTHRGSTIGSEPNSPSKSNPSSPTAKKQDGASVGQEHVRNGIDLPMPREQESLFYTGAWKVKRDSEDYKK
LYLQRNRNSSTFSKLITSRKTQRTYCRKCKIKRNLIQSRKRSGRIQVALVVVYFLCYSPAMVLDLIRRTS
DLFTSVHRESTYFLFAIAHLLVYLNSALNPIIYNCFSVQFRKEFRLTFNCCFSSSSQRRNSVRSTAALRS

LEDTKDRTSCDMML
>ci0100143701
FSSKYELIANPFLRSFVWIMGLFALGGNLASQYLQTFRRYSNKPGVSSSLRANHILVLNLAVSDLLIGVY
LILLGGTGAFYSGTYCANKLTWLSSSLCDVMGVLVVTSSETSVITMVLLTSLRLYAVLNPFTSSTRPRTS
IVLSLIAATWLISLLLAMLPHSKWLQGVFVQLYYSANSVCIPNLFVKQTTPSWTFTFLLLSLNLAAFIFV
AASYIVVYIKSSRSSIVKRNSKGFVPLERKIVRLIASDFLCWVPVSVMGFLSIGGISIPDIAYVISATIL
LPINSALNPLLYS

>ci0100143923
SITDVYVINLAIADFIFLAMLPFWIIDLAAHDRWWFGRTICKLSSASTHIHMYASVFFLAAMAVDRFIAV
VCYLNSKRYRYANIIYKRPLNLFKQQLNKCSNLCRTKHGAIGGCLVLWIMAIIMSILPYQFRDLATVNAF
ALPFVVIVVCYIWIAVFLKSRKTSGRLTSQRQDKATVMVLVIIAVFLLCWLPNQLSNFVYVGQGLGFIPA
GWSHGTAHYYIHMFSNCLAWGHSCMNPILYLFM

>ci0100143990
MNQWLETIMNERKFEIVNSSLQGLLKPDSLGMDHDVTPTVDLTDGVPQCKDLNPYVLKGDGWVPQHISRA
NRSTYSFLCVYMTFVFLLSCSLNILVIVATLKNKVLRQPLNYIIVNLAVVDLLSGFVGGFISIAANGAGY
FFWGKTMCQIEGYFVSNFGVTGLLSIAVMAFERYFVICKPFGPVRFEEKHSIFGIVITWVWSMFWNTPPL
IFWDGYDTEGLGTSCAPNWFVKEKRERLFIILYFVFCFVIPLAVIMICYGKLILTLRQVTKIDLYSLSGG
TSPEGEVTKMVVVMVTAFVFCWLPYAAFAMYNVVNPEAQIDYALGAAPAFFAKTATIYNPLIYIGLNRQF
RDCVVRMIFNGRNPWVDELVGSQVSSTGSQLTAVSSNKVAPA
>ci0100144199
VFGVIILVGLVGNAVIITVIKREHKLHTRANYLIGSLAMADLLVSLLVSPFSAVMTLHEFTPNLYFVGHH
VMCQLFTFLDVTCCTASILHLCAIAHDRYTAVTKLQYRHRTHFKKVLPCIVLIWLAAVLLSVTPYFVFPS
PTTGAKEPNTIYRIVATTIAFYAPLVIIIAIYWRVARIAWTRIHHSGKKQIMAKNNNCSPPTLQRTAMYN
LLRRNSLRHTPRHEQHLQEAFCVLSLGFRPNVTNKRRFSHDASDVDKNIYRNDSVSKVPNQFTEKTLKRS
HSHSDVPAILITADDASGGYPAKRNESPQHRVSSSKSEHSIIRTMGMVIGAFVACWLPFFIKELIVPFCG
EQCHLDPSLEVFINWLGYANSALNPFIYAFSNEEFNKAIRKLFRWNRRR

>ci0100144368
EMIGNPFLRALFWIMGLLALFGNLAVFLVTIKQMVKPVNLPVKHACLWFVANLSLSDFLMSVYLLAVSIK
GLQFSSRYCYHDQEWRSSQLCSILGALVVISTQASAFILTAMTTFRLISTFNPFLTRAVSRAWYIMPVLV
FWCIAVILGMFPLLELNSVYFFNYYGETSVCMPKLFVKVGEDTWEYSTFLITLNFALFLYMVFSYIAIYR
RSISMQSKRSGSGQNKKMQKKITRLIVTDFCSWIPICTMAYISLGGVEMNNIVYVISAAVLLPINSALNP
ILYSNVVENVIKKLCKKRNK

>ci0100144441
MKCVYWDFNNSEWLTEGCCLDISADPPECLCNHLTNFALINTDVVTADVALDIISKVGLALSIFGLSITI
LVHLLSRDVSKRRPAKVLMQICCNLLVADLIFAAGVSRTENREACFVIATLLHYFLLSTWCWMTVYSYDM
YLSLVKVFVGKATRVLQKFTLFAYLTPAVIVGTNLAIAVGYYDKQNQKNPLAASTYKADNMCWLTGYSLY
LGFLLPVGLMLMYNFTVFVMVFRELNQSNGKAQTNQIKRDYKQTLTIAVTMSMVMGLTWSVGYLLLLSDN
KTYLLAMSWIFAILNSVQVNTSIPKLEVINLKQ
>ci0100144493
MLYIDDSLFPSSSNVTTVVEIDLGVEFVTGLQDPVKIEHYDKGGTLYGQTEXCEYWDFNKNDWSSEGCCF
NYTSNPPECLCTHLTNFALIMVRICKNNNAPADLALSVISDIGCILSIIGLFATIVIHAEDRQVDLRRRR
PTKILLNICGNLMIAYLIFVVGIDHPKQKNACIAVTYLLHYFFLTSWCWMSVYSYDMYMSLVKVFRGNEH
NFLQWCSLFAYGTPLVIASITVGVTVGYLDNLPQAVKNNLNICWLNGPALYIGFLIPITICFVGNFAIFV
CVLKEIIEKNSKVEVSSMKRTVKQNMIFAVTMTSIMGLTWVFGYLLLLSDNEAYLTAMSWIFALFNTLQG
VGIFVLTAMRRPRFRKLWKNSTSAVTSSHALSVFRGTDTLRSEDASTATTDMNHRE
>ci0100144713
MNAFTFIYILTFLTTFCGAFKCSTSNEAFNDSYVCDGKVDCYDDCSDELTCGINTKFYPCPNGCVALLPL
STCLKGVKCPRFCDGVSQCSDGSDELKLGFGFKCVVQTFIEQSANRCILPQFYIKRWESVNQPRETICAA
GEDKACYDVTNDDVYFDYQQCWQCEDSTVIQRQQICDGVFDCQDLTDECLCEADDVTLNGVCDVILSNKH
KCGLEEVPCMDLSHCINKTSICNGIIDCVDGWDEEDCERVIQTTERISNYECQGGYALDETVVACDGRPD
CLHLDDECSRTCEEYCNYFLPLVLRLKFSNSCIIHIDELCNGMDECYNGEDERHCPFLTPCSPNNITMGQ
YDHIFQKCDLILHCENGADELSCSNSTHFYCDDNTTIISVRRVYDGVFDCSDFTDECSFSDTSISSVTMM
IKEPALRACVWIILAFALAGNVAVIIKTAREVWVILRGKSHSVNKLKLIHKTMVLNLSVGDVLMAFYLCI
IASKDIEFSNEYCKHELKWLSSATCSVAGILSMISSQSTVLLMAMMTTYRLHGVLRPFRTEGVSIKFLLL
VLLFIWTVSAMVAILPITPSLQRIFVSKYVLNQPFIKNSSITLTQMQDLVGRLNNLSNTKYLLDQYNPVG
SFQSIFYKENHNHLSQTSTGVLGFYSSNSVCLPKVLWMRITSLIFTDFLCWVPICIMSLLSYRGVMLPGI
VYRFAAIVLFPINSAFNPWLYSTVPVFPKLNFRSWFNKKRKVFRYSVKTDGQNIPAIQMTAIHQLQVPTS
LHHQRNLKTQEFRVPQNNLTTIWSCFSVRPFTQGSTNDLVGSSICLSSYNRSADSATVATEI
>ci0100144874
PWTTNAARVLSRKKPPQIPRPTVASICLYMIVFCMAMVGNILVVVTLALNPRMRTVTNCFLLSLAVSDLL
LAVCCMPVSLVGQILKRFIFGEILCKAIPYFMAASVSVSTFTLLALSLERYSSICHPLKSRVWQTRKHAI
KVICGIWISSFILVSPTLVFSNLSNIPIVKIGRCSPACRMTFPNRITNQAWYLFLLASMFCIPGVVMIMT
YTKICWDILNRFKLDSSFRNTAGKKMNVGTTPIFPRVTGQRQEFECNTSPFLLRSKREQNERANRALARW
KAKKRVIQMFIVIVVLYFVCWTPLYVINAWRAFEPKRAMFALEGSIHIVHLLSYISTCVNPIVYCFMNKR
FRDSFLYVFLCC

>ci0100144925

MDMLCLISGNVSYVNRSVKHDVIPIEFIAKPSIDCDVIQSEFQSLNVAVTRPSFVCTKWFKQNETLRAFA
FYGDQCQPPDDCQEHTLESTDDVSFHSVGYQPLTFILIWILAITAVVGNMMVIYNCFRKFAKKFRSFTEN
EKIHVWLVTNLAFADLLMGFYLTLFAIAHHYYQYQYNLDERYKWLSNRWCFVMGVVSFVSCQVSVTLLAI
ITTYRLRSILCPFQSRNPQFKCAIITTVITWTSWTFLSVLPLLNIEPIHTVFNNYVQPYFVQCPEKSDIH
YTSLFQTLKQATEFAQANCGDVVRNVISLPPSPTWTEMIYVGQRLGIISTDSSFSYFGFYNEQSVCMSRY
FVPFSSRSMPVTMAIITYNLILFFYIAIAYIVIWKHATTSFSCRQAICLFGNNKNCDESTRRNKREQDNF
SLQRRIMVIIVTDFLSWVPLCCLSVYYVIETSLIEQSGCDYQEYTKGWTKIFSAYTFVAIPINSSINPFV
YSTSYWRDVRSRVRRIYSTKGSPASEALDGNQL
>ci0100145160
MGSWKVLTNELAVTDIPIEQASKPCVQVAFYERDAGGYGHFHEVIQPFLPFCPFIWCGFDERFVLNGKAS
VWNCMPHSCRVNIAIVMAVSIALAIIVILVNGIVLAVLCTQSKMRTSQGIYKLSLALADILVGVIVFPTF
VSSLYKYQIVEHNIGELANVTGYVIKERATEPGQIATMSMRSTTGHFRAQFSKSYLNFVGFFTILSLTVS
ILTLVAAGIDRFIAVFRPTKYKQNIATPIAVKVTVALWALSFLFSVLPLFVPALSYTLVASILVSSAGPQ
VLVLYAFAFIVPLLLMWLVTILTFTATRSYKNSWKRLSTSDDRKAHGINEARLARTLGVMVGVFTLSILP
SIVVLICGMFMSNIYPNLPKLLSPTATTGFTSAEVVVIFILMCNSIWNCFIYSIREQEFRRAAAQKFFCR
KKSGQPNLDETDLRGLDGPIASEVFQQNEEYLDRTVFTEINLNQDKNCDDSVTTAV
>ci0100145235
MENITFIGSEPRSTSPVEIATSIACILAIFVNFCVLLVIYIGPRAFRTPTFFFISSLATADLLTGITILL
AIFLPVSDHSWERIVLKGLAVVCFSASVNNLMVIAGDSKSYLIVGVSYFVVSLIAMVGLYWAIFASVRRR
AGSKRHRNGSVVGPVASRGPFVMIHRRREIRLAKTLVIVMGVFFICWLPVVTLFVYDVIVDDKRLIVWFD
YCLASAVIHPLANPIIYSLRLPRMWCKAVGRPRYPRSTVYDTVQKKLEERRICQLQLRYQTQAQKEHRQT
EVMCSYTVASHACARPCIAARQLKWSRNETT
>ci0100145252
SSINISKEEERNRLCHQSYFNLLLPETKTFCPGIFDGYICWPHTPAGQLLTEPCPSYIEWKNVIHLQESL
VAYRFCEFDGSWMVRNDTGQSVHLDHLRGDHRTKMYVGNYLLLSIIDIVTKIYTAGYIFSLVCMVIALFI
LMFFKKLHCTRNYIHMNLMLSFIVRYVAVMVKDKVIEDHYAVGRPNLTQMEMSQYCDDVAGTDGLMVSCR
LVITLMHYATIANYFWLLAEGVYLQLLLVFVMTEYKYFPIFMAFGWGATWIPIGIWIAFRITFENVGCWE
VNNMIPIWWILRAPILISIAINFIIFINIIRMIVSKLSANNMTRSDYKYRLARSTLALIPLLGIHYIVFM
GVSDSVTDNSAFINTKFAFEIILTSLQGSIIAILYCFLNGEVQTEIRKCWRNWRWSHRLPAASGKFSTYR
SDVQMTSITYAHSMDKGDAHETSLLSPNNKHDHVDNKRSSTASCVSDQQTKIGDSSRLMCNGNGSPSKQL
VV
>ci0100145264
MLESRLQDTATLCTCKHKEAWCERLATLGTRFVSPQFRSVGPSPFTRLNTAVDNIFMQIIKLFRSYLWTN
KCRGSGVALTFLAMLLASDMLVPTSAVRYADNGPSLSGRRKPPISGLSGRQGTGMCEPIQVPMCIDIGYN

FTDMSVSPSYIYDQKEAAQSVIQFGPLTKTKCAEEMKLLVCSVYTPICIPGYPGFLPPCRFICEAAKAGC
EPILKKYDRTWPNLFDCKQFPDSQGKNPPCLHFNRSATEEPAVPRTITKKGESKTTPGGPVASVTHHPYK
PINGCPCACARRMVKITNKKDPLYGKVTTGGVPNCAMPCKSPYFSEEKRKFAESWITVWSVVCFAITMLT
VGTYLIDTQRFAYPERPIIFLSACYMFVSIGFIIRVVAGHEAVACAWHSDRVLYQTTGPFLCSIVFLLLY
FFGMAGALWWVILSLTWFLAAGLKWGSEAIASYSQYFHAAAWLIPALKSITVLAMSSVDGDVLSGICFVG
NQSTKTLRGFVLAPLVVYLALGGLFLFLGFVNLFRIRTSIKKVGKKTDTLEKLMGRIGLFSLLYMVPSAA
LVACYFYEQQNRELWAKAYNCRSFHGRDRCSHGNANGPEFAVFIVKYSMTLLIGITNGVWILSGKTITSW
RKFYRRCYGCLCGKYGLKAKQEGEPLAADDMSKASDNSSGKRTKSDKASDKTSPEEIPLTNSVATSSFTG
NPHSVGGSDSKESRSSSARKKSKTQSNGVQHNRNDMYYECNEVDRNDVNQIRHAACDCDIAPCPLPSEVR
GNPAKQLSFPEGFETLPDPLNVSTTELEAANRVQHIHHHHHHHVTQPPCSCGCHASRVTTRRPTPRRHSH
HDPGACYEANHRKLVLTPPAYRTSPTLTLPLAKAVMRFFCFSVNTQHCTPFVYAR
>ci0100145281
SSSINISKEEERNRLCHQFYFNVPLPETKTFCPGFFDRQICWPHTPAGQLSTEPCPSYIEWRNESLVAYR
FCEFDGSWMVRNDTGQSVQWVNRIQCDVATNGLQYELGPLEGGPKHNDIYTAGYIFSLVCMVIALFILMF
FKKLHCTRNYIHMNLMLSFIVRYVAVMVKDKVLEDHYAVGQTNLTQMEMSQYCDDVSCRLVITLMHYAII
ANYFWLLVEGVYLQLLLVFVMTEYKYFPIFMAFGWGAPWIPIGIWVAFRITFENVGCWEIYNALRTWWIL
SAPILISIAINFIIFINIIRMIVSKLRANNMTRSDYKYRLARSTLALIPLLGIHYIIFMGVSDSVTNSTI
TKTKFAFELILTSLQGSIIAILYCFLNGEVQTEIRKCWRNWRWSHRLPAASGKFSTYRSDVQMTSITYAH
SIDKGDAHETSLLSPNNKHDHVDNKRSSTASCVSDQQTKIGDSSRLMCNGNGSPSKQLVV
>ci0100145358
NTNNTSCGNGSFSCANGNCIHARFVCDHHNDCGDKSDERGCRIPLSANVSFIQCRNGKCIAFGLACNRKD
NCGDGSDEIRARPGLKCSFGKRDELCVLPQMYVCDGVFDCFDLSDECLCEMPQIPDVCDNICTRRKGRCK

RGTFQCGDALNVTVNFKKVCDRRIDCPNGRDEQYCRMGTHQTRVVACPADPKNMTIKTANLCDGKPECYD
LSDECGGVCSIEQAFCKFQNIFLNHYDKALVCDGYRDCADGSDEWYCPQRFNCTSSIKPSSVPRTFTFDD
FNDCVDGSDELSSRNEMISVIFFRVWVWIMAVMALLGNGYVIAKTCQRAKKLLKASAVAKANHFLVFNLA
VSDFLMGVYLICIAIYSAVFSGRYCYEDEKWRTGSSCQFLGGLSVVASEASVLILAVMTCVRLYSVFMPF
RSRNLKFWTVSICVMIVWGLSVCLAVLPIIMGLYGYYSSSSVCISRLFVTPLQQGWRISITIVSINFFVF
VFIALAYLAIYKRAKQQPRKNDTDRSVKMQSKIFRLVTTDFFCWVPICIMAFIHFARVANLDGTAYVIVA
VVLMPINSVLNPLLYSNALDNLLKKTLLAWRASRQKAALLQGEMFQRFKLQESAGTSDKAESDNQPESIH
ATPVRTADSKL
>ci0100145437
MESYPSSQIVVPIVYCIMCLIGLIGNGLVMFVIFGSKEMTKTVANIYVWNLTVADTLLLVSLPFNSTQRL
LLNWPFGSGMCKIVETVKYLNYFASVFFLTAMSIDRYIAVAYVVTSGRWRTSRNTFFVCAAIWMVSLVIV
IPLLIHTKIHGDDEKLNCKHPDSKTFHAVIIFTFVIGFVIPFIIISACYIMIIVKLQQPSGAKTRSRQAE
RTRRKITRMVVALVIAFFICRLPFYVWHLVLIPGVDVSPGVCHDVRDFTFCLGFINSCLNPILYTFLGHN
FQERLRKSISISLHSFSFTNFTKFTTNTVSRAETAAQSKKMDTSHIEVEPIVKTTQPLYHPVREEDLILA
GEKPATNGANATLLEEREK
>ci0100145494
GNRRVSNLSEPVKIVLTHNSSLQARPGFPKCVFWVPSVSYGYWSSNGCNVNHTSSTQTVCHCSHLTNFAV
LLDVYNQNNNLDPTHELILRILSYIGCGLSVTGLLITLLSYIMFGKIKRDAPAKILVCLCVSLIALNLFY
LSLTPAYYYNSKSCVAVSVLLHYFLLSALTWMGLEALNMYIALIRVFNTYYRKYILKMSLVGWGVPLIIV
VIALCLHFLTEPVYIPMDDYRICWIRKEVFYGCLVAPFALIFAFNCIIFCLVLAQLLGLRSRKLKHRDSR
KNHKKSSKHENGLMALLGITWGLAFLSIGKASLPLSYLFVIFNSTQGFWVFIFHCVLKKDIANIWQKILT
CQGYDKSNKSGSKRWRFV
>ci0100145584
FCPSMIDGLGTCFHQSKSGQTASVSCLEELNGIPYNTSDTVTRKCLEGGRWENRSEYNCRPILDEKQPCE
IHFANVVKISMAGRGLSLFTLIVAFIIFCSISYRSGCLYIIHWNFVMSLMLRNVLWICLYLFMGFSNNEN
KTIICPIMVTVFNYGQTTSYCWMFLEGIYLHRYVAIQLGNDDKLSWRFYVTVGWGFPVLIMSAWAATKSV
LETGTCWLPGQSLSNADYIFKVPVLIALLINFVIMINVIRILVVKLCNPPARRPADGSSIESTHYFKTAK
AALVLFPLLGLTYVLFIISPGYGTTGETVFLYFNTVLDSFQGFFVCLVYYYAHHDVQVEVSKKLRR

>ci0100145837
SQSFTWIYTAGGICQATVDSMGVCFTQTKPGRVAYSGCPTILNGINYIGNSSKECYMNGTWAIKSNYDSC
IHRLSQKEICKYHFVSWTVLTIVGRSISLFALCIAFASFCILRRRNLKMIIHWNFVMSLIIRNVTWFLLF
GVGFSGNVNMVGCRVFAILFNYALIVTFSWMMVEGIMIHRKLESPFMSMADSFWGRCVLLGWGVPIPIMM
FWAILKAKFENKDCWLNHSPKQVDYIYLVPIGIVLLINGFIFCNFVCILAKCRGRKAAYKHRNEASTIRS
VSKAFVFYFSLLGLTYLIFMVNPSNSSTGEIIFIYTNVILESFQGFFICLFHCFCNTHMREAVLRRVQTL
RAHGNFPCFGIGIGKSYYYYCI
>ci0100146266
MLLDCPPDASVFIPILYLAIAVIGLMANALVIYIILILKEYKKTVTNVYVVQLAIADFMFLFILPFEAAT
KLNGEWIYGTAWCKITESIRMLNYYTSILFLTMMSVDRYMAINHAMSSKAAKFRNTMAVGLISATIWFIG
FLSVIFILIQAKVQDCKCNIEFSRKFLIVWFVCNFFLAFVLPLIIIILCYVQIIRRISQPMAIGKKRVTR
MVTALVSVFIICWLPYHAFNMARANDSQLTKNVCMAVHHFTLVLAYSGRYDISNAFFLQKACMLNPFLYT
FLGTNFRKRWHSAITRTRSFRPSLGSRSGAEYTHGSGRKSRLVL
>ci0100146328
VAVSLLIVTIVGGNATMVASIVKFKKLRTTQNIFLASLAMSDLLLGILVLPFSLMRELVQHWPIGRFFCK
VYLAIDIFLCTASIWNLCIISINRYWATTHGIKYRTSKRTQRAIVLVIFAWVCYGICVFLYTRHTRCIHG
SHAIYLINRYATQAEDECHHSTETWYILFSVSGSFYVPLFIMLITYGKITYVLYKQARSGNVSYSVLTCL
YNELPAVVLYGFISIAMRRQKRLNLMIGIILGAFVVCWLPFFQTYVTQTLCPTCCCLIILYLHNRFAYCN
SAFNPFIYNLNNTKLRKAFKKIL

>ci0100147063
MVDLVAERKWNYMSVVYEDDLYGIRGNREVDIAAKKRGICIAVSRALPRNPTTGDYKKIVNDLINVNKAK
AVILFTKLNDAEGIIKAADENPQSRGRFLFIGSDGWAGQVPSQRFKFTYIASLNQSLTFQPKRNIITNLR
PYMTNLTLANHLNANKTPSTRNPWFAEYLASKLKCQLPGSIVNSTVRMCTVNDKLDPATYTTLAKMQSVA
DAVLALAHSLDNYLRDACKGLPGLCASVRKNGTKLPQNVYPYIKNVTLNGINNNTFRFDANQDGAAIYDI
ASFLGEQKWKNVGIYQNKRIRVLLRFNKVIHTRCFSIGISWYGRYNDTIDVTSVCSQPCKIGEARKIESS
VCCWQCQACFPGQYVSSNGTECMSCPIGQKPSANKDACVPLAIRVLQYDTPYAIASLIVAGLGILMTIMV
ATVYYTRRDTPIVKASGKELCSFILLGSLMSFANAFVLCYPPSDTTCIIARLFLCFGPTIIYSGLLTKTI

RVVIIFQSKKVLPPEVKMFLRPTYQIITMFAFTLLQVLAVGVWFGVRQTPAEVIFPSSSVAFRSCKDLED
VSVLAGLVVPALLMLACTVLATVNRNVPTGFNETQYIGFTMYASCVIWLAFLPIYITNTYNFGIKIASLS
ICLSLSAITVLVCLFGFRCYVILFQPELNTPKTVMSSTARPSSIQQLPSNTDANKTPNNLTTANHQPQSQ
NSVYNRAPPSTSSFQMTPIENAKL
>ci0100147271
MNNRLDCSDGSDECPDDLTERYMGMVSSNYDIIASPVLRVFLWIFGIIATVGNLLRSIPASLRKGYSASL
RTNHILVLNLAISDLIMGVYLIVLGSMGIKYGGKFCAHAKHWMGSGTCQALGVMVFVSTETSVMIMVTLT
AFRLYAVYKPLKSKFRPPVGVALSIVSFIWIISIVAALIPAMPPLQRFFTDTVLMESPFFRHGDVSFAIA
KTFVLKLLTFDPAAVNLTTVQVNMARTATSWDDLESIKFATKHGVDLQDMQYYSGLIVTDFCCWVPISIT
VFVRFSGIEVPDIMYAVSAVVLLPLNSALNPILYSDFVDGIQTKVHRKFAESASSLYRRSRTLSLRPSIT
VETNL
>ci0100147407
MKYFLVIAALLCYVTVLNAESKENSTVIAATIELKSNSSHQNVSEHQSPECVLKPRVPGQRVGSRMTIPV
ITFTASTVGNTIAIFVIWRNQKRSDKKSVFYTLVMSLAMLDLISTLVTSPVTFLAYNNNQCVMDMGGKPL
CEYSGFAMIFFGTASMAVVFAMSFERFIAIRFAFFYHVRKILTRSMIIGINAFFLLLSAMPLVGFGQIQQ
QYPGTWCFIDWKSHILAVLGTLMMVGTVVSNLYVVVRVIIKRKKMRIRNSTMQVMSKTRLKKIAYDETQM
MVLLGVMTCLRILINQLSTIYSPEADNSTTILWDLFAVRMAAINPVLDPWVRSSNANNTVVVFLTSFVGL
YSLPALDDGKVWKFNEDVVHM
>ci0100147431
MGSAAIVGNLSVFCYSIKQLVTTKYKNRVQMSNDWFILNLSAADFLMGIYLITISIKGVHFSGSYCYNDL
EWRTSKLCNTLGVFVILSTQTSLFVMCIMTTFRLMGVICPIKLRNVKFRFSALSTAMAWITATVIAIVPV
LDLSGAFEVYKIQGETGYFSDTSVCMPRLFAKVYEYTWEYSLFLISLNFTMFMYILVSYIFIYKKGTKMK
SKSKDNARSMQSRISRLILTDFICWVPICVMAYISLAGIDLNKIVYAVSAGILLPVNSALNPIIYSKFVE
DNIKKALKFVTRPCTRKKENE
>ci0100147526
LIAYIVAEVLIAVFALFSNLFVLIAIIKFKNLQTSTYWLLASLSVADISVAVLGIPSAIVLRIAVDRATC
LVVVSFVIFLTQVSIWSLLLIAVDRFVAIHYHLRYRAIVTSFRVKAGIVGAWLGGAFIGFVPQIGWTTPY
NNEDVHGSSEVERCAFEQVTSMEYMVYFNFFGCVLLPLAAMFVLYFMIFRVSLKIKKLIKVIIKLRRRSK
LIQKEFRAARSLFTVIGVFALCWLPLHILNCITLFCPECRRPNWLIDLAILLSHANSLVNPIIYAFRLRE
MKVAFQKLLRSFSNRAKSAFCCERTR

>ci0100147570
MAQVFLQTSSSILITEAKTVTSSTTAVNNIENYAVAMTTTLLGNDVINNNERHAEVVTNDVTITSCYEDS
SNSGCDLINDDVTISTTSSNQRRFVSYVTYGSKLGFANDVSFQGATSSLTSNIVSMTSHDGEKSFDDVIV
NLTFQGANACGFLTTSGNWSDSGCSVTSLNVTHVTCSCDHNTSFALLFLHSDIQFTAEELEYLDIITYIG
CGTSITALMLTIFTFVYLKLTRVKRIILHMNLAVTLCLGQIILLIGTKVEPFCKVVAVTLHLLFMTSFSW
MLMEGILLYFQSVRAVKGDVNFTLMLGFGWLLPIVIVGVSLGVGFDGYGVDKGCWLSVKNGLTWAFIGPA
LGFITLNIIMLAMIVRVFLKLKMNAKSGEWQRTRRAVKAIAFLTPLLGLTWVFGVLAVSQSTKWFIYIFS
VLNSLQGLTIFVFHCLRNEDVRKAFTKKYKRSFRNTSGVFSTVTPSSGPDSTFVRTTAGFTPRIPHKVLL
HTMF
>ci0100148112
MKSYDRQLSIIPKNEDETFTGYPVDVTTHCPCVVQTTSAYKDDASIIQFDFIQNDFCDCKRTRLPDINDH
ILGLLLMILVALIVLTNSIVMVSVCKILHRSSAHTNSRRLKISCIFNFNLSVADIMIGVAAMISLDVQGG
RSFSIGTSYNKCLILITNCTMPCVWSVASMTTICINRYLMIMHPLKYDIYLNWKRALVIVMTIWITACIV
GYSPLMGWNISEQEYLKNGSVCSFLYIVDPTYVAFLCIGTFVPQVSLVLYMYFKLYQVTRKHGCAIRRCI
YWFYPTINSMADIRALQTIALMISSYVVLWGPFLIVSCMQAFCGSSCQLQLLAGSYMFFLGICNSAVNPF
VYTYTDRSLRKEVSMQYKKLKTKKNSLRRCLHT
>ci0100148288
LVLRHSFVGKLTNQKLAEVPNLFLLKLVNMTHSEIQSGTFDNSQLRILYMNSANLDDDSIVFKLQYLLVL
NLDFNKLTKVPPLDGAPFIRLLSISGNRIRRLGARAFFSVQSTLRILWLSNNMISVVHPDSFQGLISLQH
LDLSNNCITSLENGFLNELHDLSSLFLNGNNILKVQLCLTAVPNFIKKCSVLVSLDLTNNKILIIFKQDF
MQLSNLTMLKLQANDLMEIESGSFNNLKQLETLDISGNTQVVYDNNLLSEQKKLKRVYGNNFRICCLARK
TSPSLLQCSPEDPISSCDRLIAKDYIRALIWIQAFIAIIGNAMVAVLRAREYPKQESRGGKVNTSFVGVL
SVADLLMGIYLLMIAVVDSVHQVEFFAFSHIWRHSGFCRFCGFLSLFSAEASVFTLLVITFTRMVSIVSP
IYYLRANLRIFRIAMLLAWTVALIISVIPVFRLPNFFSQTSLCLPAFYTSKRDSDWIFSLFVIIFNLVAF
FAMVTSYLVIIIATQRSQTRSENRRSSNPRPKHNLGRRVFLIVATDFCCWIPVCILGFVSIGSGGLNFYN
DVYPWTAVILLPINSSMNPLIYTFLTRQSLCQAINQIRTDTGPRTSSTGTGK

>ci0100148347
CVFWDFGTSDWSSEGCGLDVTKQPPTCECSHLTNFALLVSIHPLPPDVVLNVLSKIGSAISMFCFLATAL
IIVANKEARKQSIMIVHLHICINLLVVYSFFLFGVSATNNNNLCTTITIILHLAFLTSFMWMAVATNNNN
LCTTITIILHLAFLTSFMWMAVYSNKMYFSLIKIMAGVGEDYLLKASIVAYGVPVCIVGLNAGIALGHQQ
GNDVMSSQTYKSPQICWLHGKSLIYGFLLPVGLVLTNNLIVFGLVIRQVVFKTTKIRSTAKKRTKQQNIM
MSCAMSLFLGLTWLLCYFMLLSSNEVYQRILNWLFVVSTSVQ

>ci0100148350
MVICSILAVAIVVANVSVILVFMLNKKLRNSQGVYKMSLACSDFLVGVVVVPSIPVSLNMRLNIPEVMGM
SYNASNLSVQHDIRFDDVPHLPGINTVYYRSYLNAIGFFTTTSFIISIYSLMMASFDRFSIVRKPLAYSK
DGAKLLAIRATVILWILSIILGVLPIFVGRSLRYGLLASTLISTSGTASLSLYVVAFAFPLIVMWVLSIS
TFVMTVRHNNRTRKLVASKNKTLSSRTEIRMARTLGIMVGVFTLSLLPVICVILASLFLVQIYPQRILTF
KSYDFSIHTSFEFFSAVILVCNSLWNFFIYNGRNDDFRMANKNMYGSLTKRFHFRTKISITSSDSAPKTV
GRRTFTSETSKQNSQRISQQTIPSTPPNGKKDFSTVQSDNSPMSVLVTSKV
>ci0100148371
LNYLVFRALRTRKTTPVLLHISVCMCLSYITFIGTINLPHLEYVCYGGTIFLHYLLLATWGWTCVNALYM
YRALVMVLAADIERPILKGGIFAYGMPAVVVATNALVTLLYFDNQLDKTSKQNMCWLNTYSLYYGFLAPV
GIMLLFNITMFYCVLRKLTWGRQQLQSTAGVQTAKDQLQITLLLVIMMGLTWTIGYLMLISTDVVYLKIM
SYLFTIFNVLQGLMMFLLNCVTKKAIRDMWMPKCDLHCLDEANFTYCCRHSGSYDVTEFQSRGTILTLST
AAESSSAAGTSTAGRYSVQSF
>ci0100148400
IYTDTQRCNGHNDCGDWSDERNCSHVCKSHNETACECHLNGTCDISNYVYYWPCFSEARRCNGKNDCGDW
SDERNCTRICASHETECACNANGGTCPKFWGYHKHCYKNKYKCDDDRDCEDFSDETNCTCGTESVKCGCI
LNPNNCTSESLLCDGVPDCNDGSDEWESICSASRFYCKNKQPLSVSRDRVENGIKDCSDGSDECPPVSSI
NHVFSSPFEMIGITFFRVIFWVMGFIALLGNIVVFTASVLELKTGVNSDPVKTSFLTFLINLSVSDSLMG
IYLIAISGVGVQFTGSYCHHDAHWRSSSLCSFLGTLVIISTEVSALIMASMATFRLASVYFPIKMINAKQ
ISYVIPSACAWVIGVLLACIPSMSQSGYFVNTLCATSVCMPKLFVKTEGDEAWEYSTFLIVFNFILFIYI
ALAYACLFRRSNAVIKGSNKKSKKLLQTVSIMILCNFCCWIPICLMAFVSLSGVQLDNIVYVISAGILLP
INSVLNPIIYSKVALTTIRRLFEKQRKTFTSQ
>ci0100148454
VLSILIWMMSVLAIGGNLIVITLTLCTLWKSRSNHLLVLNLAVADFMMGIYLILLAIQNAKTNGRYCYVD
REWRSSTACSSLGTLAMTSVQSSVMFLVILTTYRMVAVLRPFRADYMHVKYTIFIVSMAWGISLILAILP
HMNFEVKGYFGFYNVHSVCLPQLFVTVNDSAWAYSAIIMTFDLIAFLYILISYLVIYHDARRSAAMNRND
DENRIMQARITRLVVTDFACWGPVCVMAFLNLLGVDMPDVAYAFAAIILLPINSALNPILYS

>ci0100148475
STKDVVFSSPFEMIGSTFFRVIFWVMGFVALVGNVIVFISSVLELKTGVRMEPIKISFHAFLLNLAVSDS
LMGIYLLAISGIGVQFSGSYCHHDAQWRSSSRCSFLGTLVVISTQVSALIMAALATFRLISVYFPIKMKY
VKKSSYIGSAISAWVIGVLLGCIPSMSQSGYFFGYFGATSVCMPKLFVNVGDDAWEYSAFLITFNFILFI
YIALAYICLFRKSRKSLNKRNKMSKKLLETVFTMILSNFCCWIPICLLGFISLSGVHLDKIVYVVSAGLL

LPINSVLNPLIYS

>ci0100148961
MEIDFGFARTVYGVALLLMVFITLLGYAVYFGAIWRSKTLQTRHIWLTSLACGDIIMMVHLILESLSSLG
MGHRPRQNFECQVGALVGLFSGYVTIASITWIAIDRYYRQCKPEKVGVNYCFYVIIVWAMSFLAASGPAL
GFGAYESAEENTVKCLIDLNKKDTNSRLYIILVSAVWFVYPFVKMILYNKKLVQEAKEPQPMAFAVPLTF
FLCYLPFAIYASLKITVGLPPLNSMVVASIYMLPKVISVVNPYLYMRSDPELLAACRHVVGLTDGKKAV
>ci0100149095
MTARIPTLSTLSTIMMNGSFTEDSHVLNGTGCKQSEGNAIHVVGTILSLIIIVENALVLMALTMERRRFK
LILFVFINSLAASDLLSGIVFLYTFLFNDLLSLEFTASAPSWVFRKAFLIASLLVSLGSLQLIALDRFVS
VAWPLEHEKFISRRRAVILVVLNWVLGAALIIAPVAGWNCVQHCVCETATLDGSPNCPHPSCSRVFPPIT
NDYIITLKSHFARMAVTRKSAKSKREEKNLAVFMVLVFVVFILCWMPSVILLCIDSSMPSVRISDDLFDI
CSFPALLNSIINPWLYAIKLKSCRTVLVQWLCRCMKQEKRDKIFQINNSVPSVVATNNRAYSVNKPCIVV
QSDPSPKPTNVAMKADVSAANHRMELKPLQPNVGEFQNVESVRSSYCSDSG
>ci0100149471
MLLSLSLTFTLLFCYWTGTMYYIEGSVTYKYYKETLDLVGLRPNPAIVPQQRDARIYRVTRDVGQDTGNL

SESYGGASSNFSGKSQQLNLLPIAFNVTMNITSTHDPSDRMVTDVTATTEDHHEDEENPFAQSPYAIFGW
SVVYGLLVVVALVGNLGICWIVIRNKRMQTVTNFFLASTAFADSNVIGFNTVFNFTYALNNDWYFGKAFC
HFINFVPIGAVLASILSITVISLDRFVVIMYPLRRRTSRKTAKMTIAGIWLFSLGVAFPQCFFATITTEE
SGTRTTCSIQWPDGVSGRMRYQLSFMVISYFLPLIILAVSYVAMALRLCGSNNQVGHQNETQLRRIANNK
KAVRMMMLVVVVFAICWCPYHLFFLADYIVSDSYHWEKIQQVYLAVFWVAMSSSMYNPFIYCWNNSRFKE
SFRELFHCGGARGHRSFAFRLRQRSRGA
>ci0100149551
MLSIANSQKCDAVYPPDCLDSSDEINCSDSTHFYCESGTPFFVRRDQVVDGKRDCADASDECPPDMFQTN
ALSSREELIKVPFLRAMVWVMAFVAIIGNCVVFILSSKSLIKNSKKAKNARAPGVVVINKILILNLSIAD
LLMGIALLIIGIKSAQFSGQYCWRDLLWRSSTTCDVIGVLSVLSCEASVLSLVCLTSYRVYTIYFPLKSR
TVNMKVAIIWIMCIWVVCFILAFLPLVDQLSLGYFGYYSASAVCLPRYFKLPTDQFEVNAISPVIMSFNF
IALLYICAAYAAIYKRTYSGNMAVDTAQRQQTEQRQKNAKKMQRKISILILTDLCCWLPVCLMTFISLRK
AGLHDSVYSFSAIILLPINSSLNPIIYTDAVPKLYTKLKQIGLQVRTLVNVDKTQIPSTTETPNVTTTSV

>ci0100149830
SDWMKCDGFNDCGDWSDENDCISECDTVNHKPCDCFFNGTCDRKDKVYFWPCYQSLYACDGWNYCGDWSD
ERGCNFTCGRDEFQCDCDKNGQGCPIGSDYISHCYKQSDICDGYPLCTDWSDEKNCTCEPGQLKCGCMLN
STNCTSNLGCVDVNYLLDGYGRCEDKSDEACQIKCVKYAKTLSQGFKCRAKSEYFQLKQSCVLPQINLYN
NNSYCEDGSDNCFVDGKLKCFRCLDGKLMISGKQVCDGVIDCYDLSDEWASRVCGFGFDEVNCTERFYCT
NSTKDAVSIKQDLVCDGVHDCKDGSDELESLCQYSRFYCLNKQPLSVERSRVENGFKDCSDGSDECPTSV

FSSPFEMIGNPVFRAFFWVMGIVCLVGNGGVFLHTVLSLLRPDLTQIKASLHWFVLNLSISDFLMGVYLV
AISSKGVEYSGRYCYHDAEWRTSNHCSGFGSLTIISSEVSALTMAVMATFRLISVYEPFKMTHVKWVTFV
MPTVAAWIVGILLGILPLLNLKSGYFFGYFGETSVCMPKLFSHVGSEAWEYSTALICINFFLFIYMVACY
LFMYKRSTKMKFDAQRSNKLQKTISLLILTNFCCWIPICIMAFVSLSGVQLDRIVYVISAGVLLPINSVI
NPIIYSDVLTIVVRKITK
>ci0100150116
GIYTDNQKCNGYNDCGDWSDEKNCSHVCELANQTPCSCHLNGSCDRSNRIYYWPCFSERRRCDGKNHCGD
WSDEQNCEYTCASNERECDCRKNGGTCSKFGRYHRQCYKDRYTCDEDRDCEDFSDETNCTCGSRSVKCGC
ILNPNNCTSSLGCVEIKDLLDGTQHCKDFSDESCEIIKTKWICSYKYYIRNGFVQCEDKGWVIGPNLCNG
FSDCADGTDELYDVPGFKCRAVNDYQQHKPSCVLAQKNLRNNNSYCEDKSDICYVDGKLKCFQCLDKKLL
LSPMQVCDGVVDCYDVSDECLCENQTLCDEGRFYCHNKTKLLFSIGKSLLCDGVPDCTDGSDELESVCSD
SRFYCKSKQPLSIARDRVENGIKDCSDGSDECPPVSSKSHVFSSPFEMIGSTWFRVIFWLLGSLALLGNI
VVFTTSVLELIISGNTDLLKTSFLTFLINLSVSDSLMGIYLLAISGVGVQFSGSYCHHDAQWRSSSLCSF
LGTLVVISTEVSALIMATMATFRLFSVYFPIKMRNVKNLGYIIPSICAWLIGILLACIPSMGQSGYFFGY
FSATSVCMPKLFVKSGDDEAWEYSTFLIVFNFILFIYIALVYACLFRKSNKITKGSNKRSKKLLQTIFKM
ILSNFCCWIPICLMAFISLSGVHLDNIVYVVSAGILLPINSVLNPIIYSKVALTTIQKLRGKQTYTVTT
>ci0100150391
MIYLDRRSELRTVAGRNLINLVVALFASQLLFLIGSEWTNPPSVCTMFTILLHYLFLATFFWNNVMAYDV
WLTFGQGKFFCSICSAEIKYMFYGWGMPGLFVGVCAILEFCVPTIYFGYSYDPSVPICWITNPTANLIAF
GVPLGAILVCNSVFFVLSIVSIRKVTSKKYRKRYSESQSMGLARVNILLYVKMSSVMGFTWALGFAAVVA
ETPVLWYIFEISVPLQGVFILVSFVCKRRILKLYSAKFGCAPKPGITGKHMHKNGNLLRHTTYIEIYQRF

>ci0100150579
MAVPDNKRALRAWLCRGITEARHNSWGPKFILFFFSGFLPSTVAVKARSHDEIACQDYEMTLRCPGSDVI
NVQMASYGRRDDRTCASSPENMMDTHCDLPGVLAIVANRCNGKTECTLTVGEGIFEDPCPGTRKYLSATW
ECVPLXHFLPWGVGKCDVTKAIRHNANGKCSVGERPIPGNRKXIYVMGWAKYDTPQLYEFSTTDKMSNGQ
ATTFHHLPYRKDGTGFVVYDGSIFYNKERSRTVVRYDFGTASTAAQAELPGANYHGTSPYAIEMSTDIDM
AVDESGLWAIYATEDNDGNIVLSRLEPYSLKILNSWRTGYDKLSALNAFIVCGVLYAVNYETLMIDYMYN
TTSNLDKQINIPIPGITRTTSSIEYNSKEHKLYMWDQGVAVEYSIDFVPITAAPPTTTTTIVLPTISSTV
SPTTTTTPQSPVSRITTTTTTPTVAKLCKGEKASGLMWPDTAYSQIAILPCPGVSAVQAIWICGGNKSHP
KWTTNEPDLSQCTSQWIKDLMIELGVKSASELSVKFVSLLRQKQQELASWDISMSIKLLRDFLDHAEHEP
LSSAATITDSTLKAASLLLDPVLTPTWKGLGEVVQTQLAVQLATILEDASYLLAKDMATEIPPPPPTSSM
ATSRTGQVNNVNWSQPFVTLGIKVAPIDLPSSAKSRKFQSKARTRATGGSDPKEHFTHWRTGHSDTVKVN
NLALVEASNPGSSDKLAVVFISCKTLGIYMEDKNSIGSPRKPVDSQVISISMRSINISSNNSPVLSSSSG
HEVELKKPIQMTLQHRNVGFRHHVCAYWDNSGKWWRAGCRRTSGNSTHSTCECDHMTNFVVLSSNEPFPA
MGADVSNPPIVQPSTQPFIYLNVVRAGLVTAAIMLVFVEVTLIVYSKSLDVNTIHKNLVASLLLSQVVFL

CGINKVNTPTLCSVVAGILHFGILSVFTWSTLEAYHLLAALNDVLTRNNRWKWYYITGYGLPAAVVMVSA
AVNHDGYGSNDACWLNSNSDFIWSFIGPCSILISFSVVFLFLVLYKLHSYNTNFPEIVEAKVTRTGAAKA
CVLTCLLCLMWSFGVMWVSKVEPQLSACLFCVFCAFHALGCCLLYCLIPVDVRQVYGKCFPGLKTSPHRP
NGRHRTTRGIIDGTSGVRLVSTDPTMRHDGTSYSAYHFTAGSSASGALLPVHNSQTLGLSKTAAMKEEGR
TAASTSMSLPHDLVASSNVSREQRCMTHGPIRSNNVFDDTEPKLPTSVACRSAGPPCNQYLHSNSRTCFD
GQHPNTHYEKPQYCECSLAHSQCSGRYDHLHECEQLRSPLRQHPHHPSCETHRSYTNTNPHLYETLGPFT
DSLRMASADGSSTPNGHLCEQCPSAVPYRHRYEDVEPRVVHVSTPQQSLAGSSSDVQHTKPQPRLRCLND
VESGEDTLGKSRENSSIGRKYIKMGTSGLRDPNNCGCYYNTARTGRSSDPLSTDQGYDTDSQQPNHLCAN
PNDSGELPPTPPSNLLVRSVPLISEEQTRLRNIHSNPSVANSVRKSVGSVHEDPASDASDSSSENDREVE
LLTSAQSSRSVVDHESEITQLYDELAASTSQPQSYGQYPSHETEPCLPSDAVDRLSPTGTVSSWAAVPGS
NPRSVNPSYGKRNSRKTTTDPKVILANARRRQESEESRHSDHLEHRNTSYTLPGTAAAGTKARIDLRTGG
ISMVTNL
>ci0100150689
SPDPLKTVVLPSVFGIVCIVGLLGNALVMYIIQVKIRKNSLTDIYVINLAVADFIFLAMLPFWSTELAMN
GRWVFGRVLCKLTSGSTHFHMYASVFFLTAMAVDRLMAVVFYRSCMGNRTRRAAKVGSAVIWVISIIISI
VPFYFQIISKTRSGNIRHCALTVSILYPTTVLYTMVLGLLSVIAFTLPFLIISICYTWIVVFLKTRKTSG
KLTSKRQVWLISSTTMVLVVIAVFLLCWLPNQVSNAIYAAQSFNLILHYYIHMFSTCLAWAHSCVNPMLY
CFMREDLK

>ci0100150802
GVIGPSSSAVSIPTAKLLGLFDFSQISYAATSPNLSDKQRYPYFFRTTSSDKFQVRAIIQVLQMNGWEYV
SFIHDNDDYGNAASEEFQKQITETNICLSANIRFPRNAGKKVINDVAQSLIRETRSRGNLFCFRNNLYLV
NVCIIISXLGYGNRFQWIGSDIWNGFQVQDLQHDTNDEALHQVMDGSLSFSGHSRPDPEFENYLKPITLN
ESLDRNLFYAHYLASLHGCSLPGSSSVQQNVCLGNLTLPNSTELSHMLQTSANAVLAFAHAFNDAQQDLC
NGTGVPVCDAFTKLNGIQIRKYLQNVSFIDGNQERFQFDQFQDGPASYDIWRYNGTEQKWKLVGYYAKEK
ITFTDNRSEKGRGNITSVCSDPCALNQYMLVSEERSCCWHCITCKENSIVIYNVSNSGRNVSKCQSCGNQ
QMTDSTFQTCLPVPVDYLSFSHGWAWGVICVSCVGLVLTICTGIVYICHWDTPIVRASGRELCCMILVGL
LLTFTLSFFFCFEPTKPLCVMRRLSTGLCLTLVYSAILVKTVRISLIFNTLGKKLVRDYKKLLKPMPQLI
IAIVLSAVEVFLLIIWFVMQPPNIVKSRSHKEVVLVSRISLFGFLINVVYQNFQVILIGLFYPILLVLVC
TYYAIKVRKVPAGFNEAKHIGFAVYTALVICIAQIPTYVVNTSSNIVLRDAIYCLGLSLNGLVILFAMFG
PKTYIIIFRPQKN

>ci0100150840
PNVPFAVVSVALVVLGLFGNMVVAFIILVLQEYKKSVANWYILQLALADTLFLLMLPFTASSELVGQWTY
GVELCKVKEAILFINYYASIYFLVVMSFDRYVAVTKAFASSSLVTRLRSPEASYIFTTAGWIISILFSVP
LFMYSSVSGCHCAYQFPSYGHEYGYMESYCQHVNFVFAFCLPLILISFFYGMIIKTIMQSKKVGNNEAQR
SYRRRVTTIVLALVFLFVASWLPWHSFQLAKIVGFPMPAESCTNFQYGVRITAYLSSALNPFLY

>ci0100150930
MAAGNSTTAAPSLLDLFNQQPQCNKPSQTGCVDLGNTTCFGSDLPYSQTSFELVTDADTLAGVKEKLLLW
SGIRAVPLCWDAIQPLLCSVYFPECGENKIQLIDRKVCQATRQPCKIVEVEYGWPDFLRCDNNDVYADQC
ESPSTKLLKFNASGCEPPLMATTNPASYYDGVDGCGLQCMNPLFNEQQHVDMHVFIAVLAGLTCICTLFA
MLSFFVDWKNSNKYPARIIFYINLCFFMACIGWLAQFFSDARNEIVCRRDGTMRLGEPVGTGESPSCIVI
FVLVYFFLMAGIIWFDILSYSWFIMYKALNTRKDPLVGKTRKFHLAAWCIPFVLVLACLGVSQVDGDSMS
GICFIGYKNHMYRVGFLLVPVGLVLFIAGFFLLRGLAALFSLKNQQSGLLSDSAVSKITWTMARIGIFTL
IAFVFVFITFAVHVYELTHQAX

>ci0100151225
MEKQFCSRSGERVYGVYWGRTERCNGYNDCGDWSDERNCSDVCEFYNETTCACHLNGSCDRSNLIYFWPC
YSERDRCDGKNHCGDWSDEQNCESTCASNEYECDCHKNGGTCSTFGRYNKQCYKDRHICDEDRDCEDFSD
ETNCTCGRDSVKCGCILNPNNCTSNLGCVAIKDLFDGEQHCKDFSDESCEIIEVNTRRYVPYYNDEVQKE
VATTSFFLCNKSNYDYILELNKTRPNKRSCDQIDFYSSDSLTLTETKWICSYTYLLQNGFVQCEDKGWVI
GPNLCNGFSDCADGTDELYDVPGFKCRAVNDYQQHKQSCVLPQKNLRNNNSYCEDKSDICYVDGKLKCFQ
CLDGKLLLSPMQVCDGVVDCYDVSDECLCEDQTLCKEVLGDSKKLCSTGQILCNGECLPSEQVTCNKSLN
CDGGSNTKYCNKPNANNTVARTGTGHIFCPTDRLFTIYKNATMCDGIPECYDRADECDAGCPNQTHFCDV
PLRCHQRQASAVWEKRGQLFLHKREYCDGIPLAFVPLCNTGFDETYCQGRYYCHNKTDLLFSIGNLFLCD
GVPDCTDGSDEWESVCSASRFYCKSKQPLSIARDRVENGIKDCSDGSDECPPVSSRNLVFSSPFEMIGST

WFRVIFWIMGFVALLGNIVVFTASVLELKSSAHTEPVKSSFLTFLINLSISDSLMGIYLLAISGVGVQFS
GSYCHHDAQWRSSRLCSFLGTLVVISTEVSALIMATMATFRLVSVYFPIKMKNVKKHSYIIPSICAWMIG
ILLACIPSMGKTGYFFGYFSATSVCMPKLFVKTKDDDAWEYSTFLIVFNFILFIYIALTYGCLILRSKKV
LKGSNKKSKKLLQTVYKMILSNFCCWIPICIMAFISLSGVKLNNIVYVVSAGILLPINSVLNPIIYSKVA
LATIRKLWEKQLSSVNM
>ci0100151327
CKGEWDKITCWPNSAPGRTVRLPCPEYIIDFDHTGICHALRHCSRDGRWAMVRDTNRTFSDYSSCNIPRE
EDVVEMIRRGGDLYTVGYSFSLVALVFAMIILAYFKRLHCTRNYIHMHLFASFILRAVVIFVKDRVLYYG
AGILDINTPDGEMTLEALKNRVDEIDADRSSYLIGCKLVMTLFHYFVATNYYWILVEALYLHSLIFVAFF
SDKKYLWRFSVTGWGVPILFVVPWAIVRAKFEDTGCWDIAVTEYKWIYNGPIIVANVINFLLFLNIIRVL
WYKMRERGPIGKTDNRRQYKKLAKSTLVLIPMFGVHAIVFIGMPDDISSGTWWDIRMSFDLFFNSFQGFF
VAIIYCFCNGEVQAEFRKAWERFNLSVEIKRGRRERSRSSVTMLTSFNSSASQQVRIMTS

>ci0100151346
MTEVGLNFSFCGEEAKLLTEANFTKCGRQVFCGTLVFCEYEAMMNTSLNFNASLKCSTAGLIINLVIMSL
LSFIIIVGNGLVIYIMIKKRQFHNFQMCKMSLAFADILMGLIVIPGLIESMVLMFFRANPEGLSFLCLYE
RQSQAHRGKIVLYGGAATGSVAASLYSLLVLSGDRFFAVVRPFIYQRVIAPKRHIVKIILIVVWMVSIFL
TSIPLFWDDITYRLPIWMFASAPLGSPCNMIASVFPIVVLAIPYGATVGLTLATLKITTRNLAEAEAKRG
MGRGSLVRKESLVSTRFSAFQNNSETRQVTYKSFMGKFYYAFMVYWLSPLHTNWWL
>ci0100151424
MSVLNLLVLCWVVGSSPWVSSRTSGTAGVAMADSQPPQTRKSPPCPQACRCSRNTGFGYSVDCSSLDLNQ
VPSSVDELTTFLDLSVNNISVVASKAFSNLHLLQELHLEHNQLTEYPRPVTGLTKLRELKLNDNKIQRLP
DRAFSKQTNLEILMFHNNPLKSVGRWTFTNLRNLELLEIVGVEEQEHFPDLSGCNKLRELFVVGGRFTQI
PDTLCGKKSVLQRLIVANNRLQNLGGISNCKMLDGLDISGNALKSLNENEMSNLTFLRTLDLSENKISSL
AENVFGHSKLVKLNLAYNSFAYLPIRGLSQLRKLNVSGVKNMLYLPPIQFMNHLSQLEAAYPYHCCQVSN
EVNNLIKDTDLIFQFTYELWVRAHEYEWVGPDDEIIKQSSPAISCHPEPSALTPCDNLLPSVVVRVVLWL
VLVFSSALNMTVFVQSYDSTGSVSDTFELALCHLAFADFVAGVYIAILVAYDVTTRGNFAEHGAWWQQSL
MCRTAGFFLVMGLQLSFFTVLIATVERYLATMFPLKPEKHISKAVLGLTLSAAWVISIATAIVTLVSGEE
GFPLQERSRRVLPLYNGATCLPWSTHFKYVAVLVGGHVIACLGIVILCVLMYCARRKQSWLSAQKHTRRQ
ISLTVACNVTLILPLALIGLLASAEPAKLNVDLLLTMVMLLISLRFAINPLLYISFSESFRRDMRAAAGW
LCCTIRRNRY

>ci0100151600
MYSNSSQQQSSQSEATASIVAICIVSALASVLTVGGNVMVIVSFVINRSLRTVNNYLLLSLAAADLLIGL
VSMNLFTVFIVSGSWNLGAVLCDLWLALDYVASNASVMNLLIICLDRYYSVTKPVEYRNRRTPKRFITAI
IGAWSVSFILWAPWVIFWQFIVNERTVPNDNCYVQFIKDSKPMAVITALAAFYLPATVMCVLYWKVYLGI
KNRRKKIASSHVKTLASEIYKNENAALDLPPVEPQPVEEGNVFNDSVFASPADPPTPSDESPAHVLKNYD
AGGEVPEPSVRMTSPSDVTGARQMLVAVIRQRHGYIEEKVEAKNAIDNMEDDSSDFETFDCTNGNLIIEN
NAKSPKRHSFKSYKGKTKRPETSSRSRTNSGKVMFIRRQIKEKKSSDTESAADVGGVIKVTERLKLQVVK
KKLQSLNKETKAARLLASILAAFILLWLPYNIMALIEAFCPLSEKCIPDLAWSFGYWSCYLNSTLNPVCY
AMCNKNFQETFKFLLSLKWVHPDHRRFRPQMV
>ci0100152008
MLQANYYRQRCVFWDFATSDWSSEGCGLDVTKQPPTCECSHLTNFALLVVSLIYSIINHKVTYVVTWSHA
VIYNVCIAAYVAIIIILKIRNKTSTKIQLHVCFNLMVAYLLFMIGVPSVGNKTTCTIVAVLLHYTFLTSW
FWMGIYSNRLYVCLVKVFPRPTTNYTWKSSIFAYGVPLIIVSINAGLTLADNVCWLHSWSLYFGFLLFVG
IILLNNLVVFVLVLNQLCWRESEIRSSANKQRKSHKVMLACSMIFMLGLTWMVGFLMVFSTNVVYNKVTS
WLFTICITLQVTKGGVYLRMAGQRQLL
>ci0100152581
FFCSIGYIIYFYVITKRKHLRRKYVWMTSLALADFFFALNLLPILISLVKDKWVFGIYGGLVNSILSLSS
GFVGIAGAQGIAIHRHEVEQGKGCQLKYIYFRIGMAWVTGIAMGVVPSLGIGHYEEATETSFGCLLDMSK
SDLSSFIYMVACFGFFAVFPLWRMVTSYYKLIKMNKANWVSTSVLVIPVVFVLCYAPFSVHALAAVKIPH
HVPSELEIVLVHCGPKILIAFNPFMYVLTNTELLSAYKSVI

>ci0100152622
QTWFQFDTLHLVRVLVTWLLFCLSMAGNMFVLWSLRGSKSRHFIMFHLALSNLVYTIFVMPSDAVWNTTM
EWLAGDVMCRLCQMMKQFGMYASSFMVVVIGADRVTGILSPLPCHSQRKRGYYMVATAWISSLICCLPAG
FIFSVASIPTCEGIPIYQCIDFNVLQDVSLLRPYYFFTMCMSFLLPLICTLVSYSLIVCEISNMKERDRV

LMGRRHSVNTASIQRAKNRTILMRTLITLTFLVCWGPYYGKGIYDWFIRYEDHTPPDAWDTVMYVVMYLN
PVLHPIVFGVFLKEIRGKFKQRLNCARKRFFKQRDFKTVPNAQSSMNYSIASVLNRPRRMSSTSRGSFSS
YATGATHLNGSSHVTINGQCSNNGSNGSIKTQPHFFGANRMAAPQQQLLLSSESAL
>ci0100152670
RNITKGGIFPMSGGWSGGQACYPAAQMAVEDVNNRSDILQGYKINLVKKDSKCDAGLGTRKFYDLLQDSR
IFTLLTGCSSVSTPIAESAYLWNINVMAYGASSPALSNRSRFPSFFRTHPSANLHNPTRIKLFQMFGWKR
IGIISQGSEVFESTAKDLRTRLIQENMEIVTQQSFHADPKVAVQNLKKVDVRIIVGLFYEGAARKVMCEA
FKEKLFGNRYVWFLIGWYKHGWHKVPEKGVDCTEDEMADTVEGHFTTEILMLNPTNDTTVSGMNTQEFLQ
RLSNTTTDIHLKGGYEEAPLAYDAIWAIAIALNNSIQRLQTSDLTRNNGLILNDMYRSLNASSFTGISGT
VVFDSDGSRMAWTKVERMENGVYKEILIYHAEKKIMKRINKINWKQPPKDRTMTVKRTLKLSTSAFVAGG
AMAAMCGFFCVACLCFTWKERNNKLIIPPGSSLNSISLFGFLLLISAIFLSGLNDITGPICEVRAWTLSI
GFTLAFSPLFAKLWTVNDLHKKMIFDGRSTKKKKKDVKFTLNTETVVVMVVPLVLDLIVLITWSCIDRLV
QSKSKLAPYEEGEVIINPELKKCVSNYTTIWIGVMFAYKAIQLLLGSFLAYESRSKIESNSDHKTVAGAV
YNITVLSLLCAPILMILGDEPNLSFLFLATPINMCTITSTAIIFIPKVNMLKXFRRTGSNRSAVQRVRPP
ASNHIESEEQKLERENRILQEKIAEV

>ci0100152713
MELEFGITRTAYGIAMLMMAAVATFGYSVYILAIWSSKKLQTKHIWLTSLACADLLMMVHLFMDGLSSFH
QGRRPKGIFECQVYAHMGLFSGFVSIASMTWICIDRYYRKFKPEKVGVNYCFYVIIVWAMSFLAASGPAL
GFGAYESAEENTVKCLIDLENTDMNTIKYFVVVGFLFFFYPIFKMIKYNTKFAYKSEEEKAVVIAAPVSF
VLGYLPYLVYACLKLTIGLPPLNQASIAFLYLLPKFISVMNPYMYMRSDPELLRAAKRVV

>ci0100152766
CVYWDEVTLTWSDVGSVMTNSSNTTLTCTFNHLTNFATLFVNDTSISNSHVLDILTVVGCSISGASCLLL
ILIFIFVPTKGRKRAAVLLLNLAIAILLLDIFLIVSEQEIVTSSMTSCLAVSVLTYFSMMSVFTWMMVEA
YLFQPFKTRGKLFGNWFMIASCLWGWLLPALVIMFTTIFNIDMYKRTDGDVTNSFTRCYIQPGLVLYAVL
IPAGITLGVNLMLYVFLTYKVTCAKRPVSMAGKSKGLQKNLLFSLTLFVTLGLTWIFGFVIIPGNGDASF
AFSVLFTVFNSLQGFFLFLLYVVRQKFTRSAISEQVRKISAFVIPTTWSTS

>ci0100153146
MTTAAFTQTYADRIYPSTATNTFLPLCQSHDTILQTWFQFDTLHLVRVLVTWLLFCLSMAGNMFVLWSLR
GSKSRHFIMFYLALSDLIYTIFVMPSDAVWNTTMEWLAGDVMCRLCQMMKQFGMYASSFMVVVIGMDRVT
AILSPLTHEGQRKRGYCMVLAAWTTSLLCCIPAGALFSLLTVETCEGINVYQCVDFNIVKDRSLLRPYYF
FTMCMSFLLPLICTLVSYSLIVCEISNMKERDRVLMGRRKSVNTASIQRAKNRTILMRTLITLTFLVCWG
PYYGKGIYDWFQKPAIGPPAPLDTAMYIVMYLNPVLHPIVFGVFMKEIRSKFKKTSCIAGLKQKREQVRS
GSVSRLSLTSYATGLTQVQDLPNGAAETTVLTGATQQVPSIDESVHTKAPE
>ci0100153351
MSDECGGNGTCTETFTCDDGSCIALSRFCDGTQDCANGEDEIESDSTLGNMAVSCSNRYQSINNKLYNTH
TNRVCSLPLRHVCDGVDHCENRTDECQEGCVHSLFCDGGDTCHHRSSLCNGESECSVRKSQVCDFVADCE
DQSDEADCHASHFYCEGGKPFFVDKRYMFDGKRDCEDGSDEFSSAENMIESRFLQVMVWVMGILAIGGNA
AVIVHTISLLCYHARRKLTKVAVVYSCLVLNLSIADLLMGVFLIALGIRSASTSGNYCLIDHSWRSGTPC
SVLGTLAVLSSEVSLLTLAILSSYRMFCVIWPIQSRTLQVHFSIGLAVSTWIFAGTIAFIPLSKIYLGYY
STHATCLPKLFVDKRDLGWQFSLTVTITNFILCLYIVGAYAVIFRPRQVNKSSVTSQGTRNMQKRIAILV
ATDCACWLPICLIAFLIFSGVEISNTVYSITAIIILPINSALNPLFYSNAVQILFTRILNYLSKFETFNA
VRKSIAGITIFKCWKTATEKREGQDGKNLLRGHEDTDGGHIVKMKSHVTEHTVLDTVMTESVV
>ci0100153483
IGQLYPTIMTHVISADIRNTVMQDLKHPVFIYHNIPNHQNFTPKIIEPDVKSKTAELCVFWDFNANNGDG
DWSQNGCSMINSSSIIGSSNKTHLVTCSCNHMTHFAVILSTTLTYEPQYLTLLTIIGCSISIICLLATLG
TNLYFRQLRTKLPQQVLMHLSGSIILLNLLFLIGIDLNKNDTSCFVVALFLHYAVLLVWCWMLVEAVFLF
RTLVITRVESSSGKCFIWSAALISYSAPAIVVAASASSLPKESYQSMKYCWLASDPLLYSIVYPIAVMLS
LNLLIFFIVMYNITYRSQMFRKNVNLRKISQAVKRALCMLVVLGLAWVLGYVMLLSQDKATKDIFTILFT
ILNSLQGFFVFILYCFRQENVRNLWLRPL

>ci0100153785
MDNLLNATVGPNLSATTATPNPWYGIYGFQRTIEIVYLVCIILLGTLGNMLVICSIKSASRTHVSGNIFI
VNLAVADLLVTAVLVPCVLANVIATENTLPNFACRVVAFLMSTACACSIHSLAAIAINRYWAIVRPCSYP
RVFSKKNTRIMVCGIWVWTCIPFVPAIMLAEQPYDYMIMECLWDDKFSNSYTISLVALLMVVPFIVICLC

YERLYHIVKKRSKWLMAQGSLENNTTMTEAALDRDIRLLKTVAVIITAYMLFWMPYGVMIIAGKLYIPPV
AKKIIGWMAFSNSAVNFIIYGVMNKSSRENYKHFLIRLLRGEFCGKAKPPTPNISVYTMNSPAGQRKFNT
QATMVESRREKQLVHKLNVV
>ci0100153810
FSSDQFLINSTILRVSVGVVGIIAVAGNILVLCQNGNRLRNARRNESDIVSSSRVLAINLALADLMIGVY
LIILGVKDVMYQGVYCYHHLKWKSSIFCTILGCVALVSIETSMFVLTVMTTCRLIVVKSPLPGSISVRKV
VWSVVLAWVVSIMFAVIPLLPSLQDTFTMAVCFGYYSSHGVCLPRIFPNSSLDKSSAFSMFLMVINFAAF
AYIVLAYLMIYRIAVVAAKKAGRTDTSRAIGLQKNITRITVTNFLCWVPVCIISFISLTGGSVDPTSYAV
AAIVLLPINSATNPFLYSEVIGSVWKTMPRLPSFRSSRDTQKADENTSPANGNFTTTSIRLSNKIYPFCS
ETT
>ci0100153844
SAVVVPMTTFALGVMSNSLAIVTLLSTVKRKSRKVKFGPFHTLVLTLAVLDLAGTLFTSPITFILYFNES
HILGGTPLCNYSAFAMMFFGFTTMAMVMTMSIERLMSIRHAYYYHRKATATKALLVVLAIFIFSALLCAM
PTLGFGKVRPMFPYSWCFADWNAQAPKDKAFNFLYVSLGVTMLFVTLVCNARVIKGLVEMKKSQVCRRKL
NYFTATVTAVEPKRNVVKLVALLGLAFLCVGRIRPVVWNKRNVISKWNRKSRRRTVSRETQMIIQLLVIT
LIFVVCWLPLMFRIFFNQLGINLSSKRDLVAIWLVAVNPILDPWVYILFRKSVARCVLRLAKTIVC

>ci0100154093
IISLLVSILIFVIVLGNFLIVAAVYNFKHLRTTQYFYVCSLATSDLLLGVLVIPLNLVREMYGYWPLGET
ICKIHLSLDILLCTASIWNLCMISISRWWILSAKSCSFLRSIPNRLCIFMVVCVWMWSALVCAWSFDAYQ
QVRSLRRFVLYRQKKLHLTTGLIMGAFVFCWFPFFQAHLTQILCPTCCVPASLLNFLAWLGYCNSALTPI
IYTTKRSELRKAFCILL

>ci0100154135
YIILEVLLGVLSIFGNALVCYVVLHSRTLRKKVMYMFVTSLAVADICVGAIAIPFAILTKLGLPRSSPFL
CVSMLSFIMIPTQISIFNLMAISVERYLSIRHPRIHYNKLTVDKSLTVVVLTWIVSVVIGMLPLFGWNKI
SNPPGKQPIPVLKLLSRTPVMDFDYMFFNFAACVLPPLITSAILYFIIFVSVLRVQEKSQHHNERHVRRV
REMRAAVVLALIVLSFAICWMPIHILNTIHSCFSFRFDPSRVIPAYVYNVAILLSHCNSTVNFFLYSWRL
KAFRIHLHKRVLNKLKRND

>ci0100154142
MENNYSQYIEPSLYWERNDSVVYELGYAIIPNRVLLPIDYYPIPDINDLENGTAAAARNQTNATAYVSVL
CGFPSFCSYSAYNVCSNTTLVECDECSMIGAGFFIFACVVLGAMILIGNSLVFWYNFRNSIQDKFSIMKA
SLSVADSLAGIQIFGVVLYNVSWTINSTSQELDMKQLLYQDSPQAIIGGMFYIMSFTSSLFHLLYFSGQR
LFAVTYPINYKIQSNAHVAFGLVLTWILGVISATVPAWFPNTYTYSYYHTIYLFLPSIRNYSSTNNSSSG
AALALVFVFSILPYCLMVILTITSALQIRIRLIRSASLSKSMDKDLLKKKEVKLIKTVAIMQIGFTVTLV
PVVVILVLFYAHVFSCKNVSTPYVIGFYMSMSNSLVNVLVYSGRDSKFRAWLKSIVTCKSVPAKEDSHTS
FSATRNHNAKATTETDNAIQTSKIVSR
>ci0100154445
MYLIPGTIGNLIAIVVYISNKKMQTAFNLLIVNLAMIDLITSSTIMPIILYGYITQSWPLSLTACEVFAF
FYYSAHFTSTINLLMITLNRYMHVTRPHATYKIIYSVKRRRILTLIAWSISPLCLLPLLFTNGFSWWEAG
YMCAYSRLEGFSNVYSLFLGVLFQLLPFLLMVVLYIFIFQTVKRSHANIDNPKIQGEYWTYDAADLSLET
NVSLHHAKTQRQLIYMSITICVAFAVCALPSVIMNLAATYEDIPPVFHMLGSNLGWLNAMINPIVYAAMN
SQFQKAY

>ci0100154507
MALVSWFLILCLLLSVICCCVKGDVCRNKSTAFGFRCTCKTLKVPCKCQCRLPHENTTWKRQKRNGVCDA
VYDCYDNFDKCRCTFNQPRTRTQVYIVSCCYNISFPKCTKPSKKKNIFASADDLIGNVYLRILFWLVGAF
AFLGNSFVFLRTVAKYTRTGNQSVKQGFRWLILNLSLSDCLMSIYLIAISIQGVIYRSRYSSAHDLIWRS
GKTCQLFGVLALTSSEASAFIMVTMTSFRLAAVCFPFKMENARQIWYRISTTIAWLFAILVGTSPAWNTK
ITGEFGYFGSTSVCMPNIFSTLEDEAWEFSMFLIILNFLLFIYMAICYLVIYKINSNPHRRQEGRKLKKR
ISILVATDFCCWVPVCVMACVSFNGTRLDPTAYIVSAGFLLPINSALNPIIYSKVLGRSINSAARWFSNK
CSRLPKSIPEKISSKLNLTSDQEMARGSLRETESSL
>ci0100154530
MALVSWFLILCLLLSVICCCVEGDVCRNKSTAFGFRCTCKTLKVPCKCQCRLPHENTPYKKQQRNVVCDA
VYDCYDHFDRCGCTFNQPRTRTQNYTVSCCFNRSYPNCTKPSQKEHTFASANDLIGNVYLRILFWLVGAF
AFLGNSFVFLRTLAKYTRTGNQSVKQGFRWLILNLSLSDCLMGVYLIAISIQGVVYQGGYLSAYDLIWRS

GKTCQLFGVLALTSSEASAFIMVTMTSFRLAAVCFPFKMENARQIWYRISTTIAWLFAILVGTSPAWNTK
ITGEFGYFGSTSVCMPNIFSTLEDEAWEFSMFLIILNFLLFIYMAICYLVIYKINSNPHRRQEGRKLKKR
ISILVATDFCCWVPVCVMACVSFNGTRLDPTAYIVSAGFLLPINSALNPIIYSKVLGRSINSAARWFSNK
CSRLPKSIPEKISSKLNLTSDQEMARGSLRETESSL
>ci0100154564
MEVNDKRVYGVLMGLLGLLTITGYSLLFVIFAKRPDLKKKNKFLLSLATSDLLITVHVFASTIAAFAPQW
PFGDLGCQVDAFIGMAPTFISIAGAALIAKDKYYRFCKPKMVGRNYSFHVYLTWTMGIIGGALPFIGFGR
YGFETDDVTWRTGCLLDFKSISAKYSFYIILISTVWFVWPVYKLVSSYMKISTKINKFYPLLFVVPVQMA
IGLLPYAIYAMVSITIGVSAVPYFCVVINNLAAKVFVGSNPFIYIYFDPELRESCKQIFCSPPAPTNDKI
SEDSKDE
