# Supplementary material for: The repertoire of G protein-coupled receptors in the sea squirt Ciona intestinalis
Source: BMC Evol Biol. 2008 May 1;8:129. doi: 10.1186/1471-2148-8-129 (PMC2396169; doi:10.1186/1471-2148-8-129)
Supplement: Additional file 3 — Domain architecture of LDLRR-GPCR/LGR-like/GLHR receptor cluster in Ciona [file 1471-2148-8-129-S3.pdf]

Domain Architecture of LDLRR-GPCR (Low density lipo-protein receptor repeat containing GPCR)/GLHR (Glycoprotein hormone receptor)/ LGR-like (Leucine rich repeat containing GPCR) receptor cluster in *Ciona*

LHCGR-like: (1 member)

>ci0100133821 (244)

A luteinizing hormone/choriogonadotropin receptor like sequence

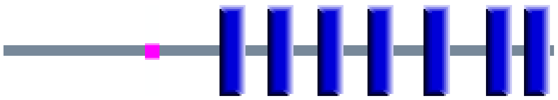

LGR-like: (2 members)

>ci0100148288 (260)

A candidate INSL3/Relaxin binding GPCR like sequence with LRR domains in its N-termini.

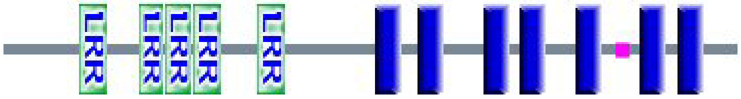

>ci0100151424 (268)

An orphan leucine rich repeat containing GPCR with LRR domains at the N-termini

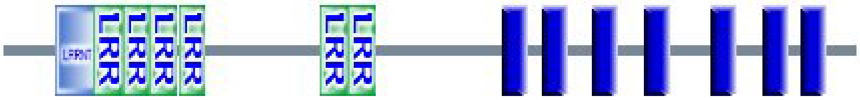

Low-density lipo-protein receptor repeat containing GPCR (LDLRR-GPCR) cluster: (14 members + 1 incorrect model)

An unifying theme among 14 LDLRR-GPCRs (excluding an incorrectly aligned model:

ci0100131758) identified from *Ciona* is that they possesses INSL3/Relaxin binding

GPCR (Swiss-Prot: LGR7_Human/LGR8_Human) like TM regions with LDL Class A domain representation at the N-termini. The LDLa domain machinery is represented either singly or in multiple repeats at the N-termini as can be seen below. All 14 LDLRR-GPCRs lack Leucine Rich Repeats in the N-termini.

>ci0100130612 (241)

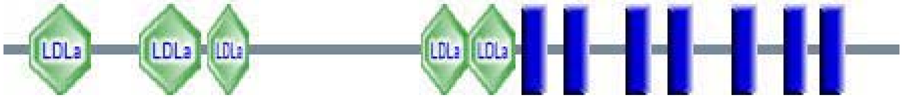

>ci0100137355 (246)

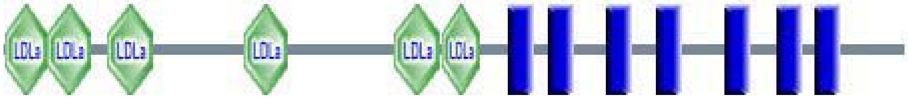

>ci0100141745 (248)

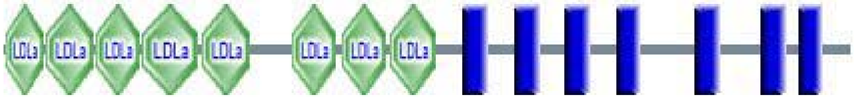

>ci0100143219 (252)

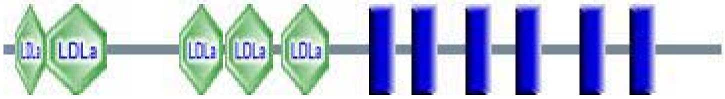

>ci0100144713 (255)

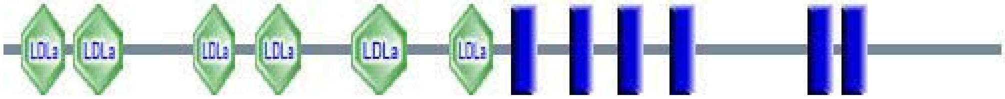

>ci0100148400 (261)

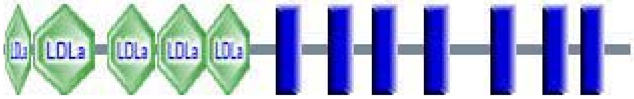

>ci0100153351 (269)

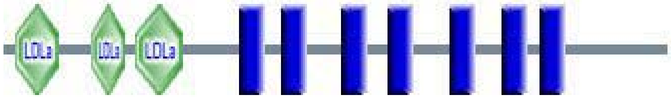

>ci0100150116 (266)

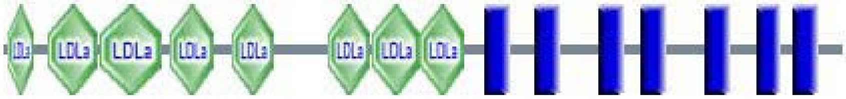

>ci0100149830 (265)

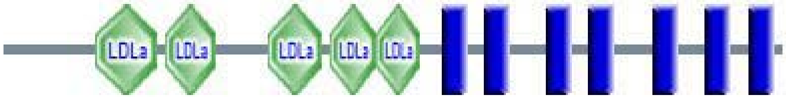

>ci0100151225 (267)

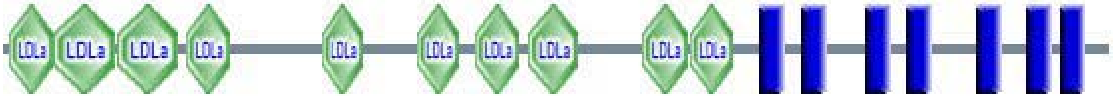

>ci0100141547 (247)

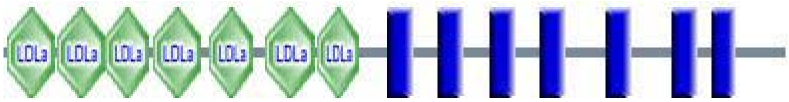

>ci0100142353 (251)

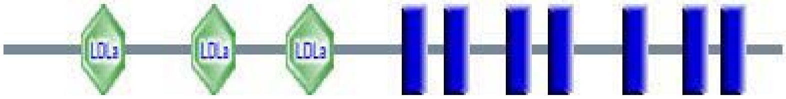

>ci0100149551 (264)

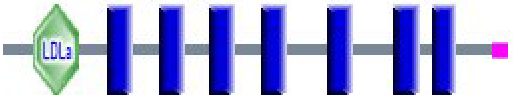

>ci0100145358 (257)

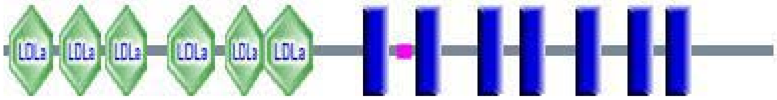

>ci0100131758 (242) (Incorrectly aligned model)

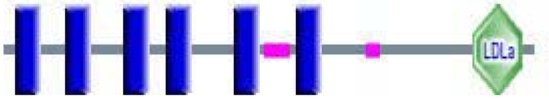

>ci0100131758 (242) (Proposed model with corrected domain alignment)

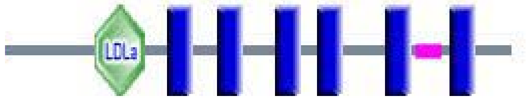

“Unclassified LGR-like” sequences :

14 more *Ciona* LGR-like sequences consisted of type C INSL3/Relaxin binding GPCR like TM regions with no recognizable or explicit domains in its N-termini. Therefore these sequences are not authentic “N-terminal Leucine rich repeat containing GPCRs” and have been designated as “Unclassified LGR-like”. It is possible that these 14 “unclassified LGR-like” sequences are fragmentary in nature or incompletely modelled at the N-termini.

>ci0100134424 (245)

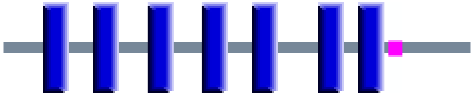

>ci0100144368 (254)

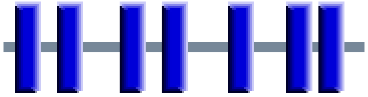

>ci0100148454 (262)

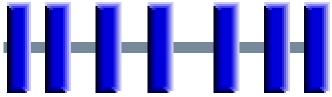

>ci0100132380 (243)

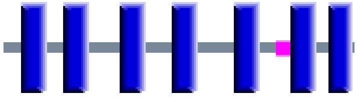

>ci0100148475 (263)

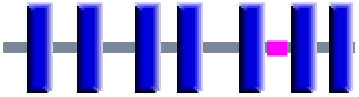

>ci0100153810 (270)

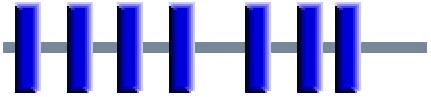

>ci0100142029 (249)

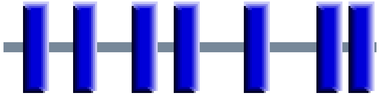

>ci0100143701 (253)

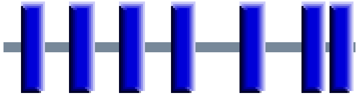

>ci0100147271 (258)

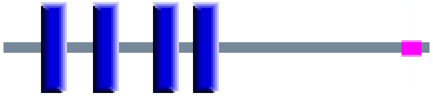

>ci0100144925 (256)

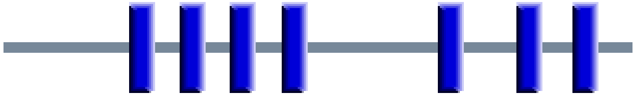

>ci0100142069 (250)

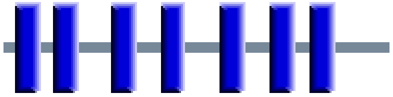

>ci0100154507 (271)

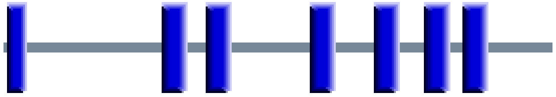

>ci0100154530 (272)

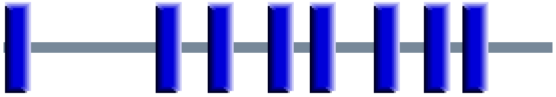

>ci0100147431 (259)

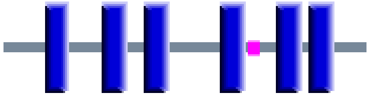

Domain architecture of INSL3/Relaxin binding GPCR like sequence from other genomes

>RXFP1 (LGR7) (Human)

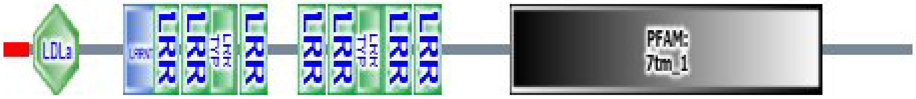

>RXFP2 (LGR8) (Human)

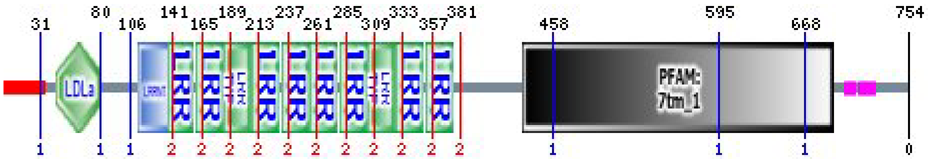

>GRL101 (Snail)

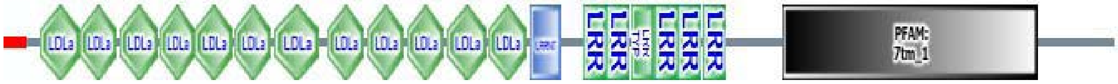

Domain architecture module representations were obtained from the SMART database [96].
