# Supplementary material for: The repertoire of G protein-coupled receptors in the sea squirt Ciona intestinalis
Source: BMC Evol Biol. 2008 May 1;8:129. doi: 10.1186/1471-2148-8-129 (PMC2396169; doi:10.1186/1471-2148-8-129)
Supplement: Additional file 4 — Multiple sequence alignment of human and rat PTHRs with homologs from Ciona. Alignment was generated in MAFFT using gap penalty of 1.53 and offset value 0.123. Scoring matrix is based on JTT200. Colored amino acids are used to refer functionally important residues known in human and rat PTHR1 and their putative homologs in Ciona and human PTHR2. Furthermore transmembrane regions are colored and sequence conservation and similarity represented symbolically. [file 1471-2148-8-129-S4.pdf]

```
28              ------------------------------------------------------------
105             ------------------------------------------------------------
PTHR2_HUMAN     MAGLGASLHVWGWLMLGSCLLAR---AQLDSDGTITIEEQIVLVLKAKVQCEL-------
PTHR2_RAT       MPWLEALPYICGWLILRSCLLVG---AQLDSDGTITIEEQIVLVMKAKMQCEL-------
PTHR1_human     M---GTARIAPGLALLLCCPVLSSAYALVDADDVMTKEEQIFLLHRAQAQCEKRLKEVLQ
PTHR1_RAT       M---GAARIAPSLALLLCCPVLSSAYALVDADDVHTKEEQIFLLHRAQAQCDKLLKEVLH


28              WPNSAPGRTVRLPCPEYIIDFDHTG--HALRHCSRDGRWAMVRDTNRTFSDYGSC-----
105             WPNSAPGRTVRLPCPEYIIDFDHTGICHALRHCSRDGRWAMVRDTNRTFSDYSSC-----
PTHR2_HUMAN     WPRGTVGKISAVPCPPYIYDFNHKG--VAFRHCNPNGTWDFMHSLNKTWANYSDCLRFLQ
PTHR2_RAT       WPRGTAGKTSAMPCPSYVYDFNHKG--VAFRHCTPNGTWDFIHGSNKTWANYSDC--FLQ
PTHR1_human     WPLGAPGEVVAVPCPDYIYDFNHKG--HAYRRCDRNGSWELVPGHNRTWANYSECVKFLT
PTHR1_RAT       WPLGAPGEVVAVPCPDYIYDFNHKG--HAYRRCDRNGSWEVVPGHNRTWANYSECLKFMT
                ** .: *.   :*** *: **:*.*   * *:*  :* * .: . *:*:::*..*

28              NI--PREEDVVEMIRRGGDLYTVGYSFSLVALVFAMIILAYFKRLHCTRNYIHMHLFASF
105             NI--PREEDVVEMIRRGGDLYTVGYSFSLVALVFAMIILAYFKRLHCTRNYIHMHLFASF
PTHR2_HUMAN     PDISIGKQEFHE---RLYVMYTVGYSISFGSLAVAILIIGYFRRLHCTRNYIHMHLFVSF
PTHR2_RAT       PDINIGKQEFHE---NLYILYTVGYSISFGSLAVAILIIGYFRRLHCTRNYIHLHLFVSF
PTHR1_human     NE--TREREVHD---RLGMIYTVGYSVSLASLTVAVLILAYFRRLHCTRNYIHMHLFLSF
PTHR1_RAT       NE--TREREVHD---RLGMIYTVGYSMSLASLTVAVLILAYFRRLHCTRNYIHMHMFLSF
                      :.:..:   .   :******.*: :*..*::*:.**:**********:*:* **

28              ILRAVVIFVKDRVLYYGAGILDINTPDGEMTLEALKNRVDE---IDADRSSYLVGCKLVM
105             ILRAVVIFVKDRVLYYGAGILDINTPDGEMTLEALKNRVDE---IDADRSSYLIGCKLVM
PTHR2_HUMAN     MLRAISIFVKDRVVHAHIGVKEL---ESLIMQDDPQNSIEA---TSVDKSQY-IGCKIAV
PTHR2_RAT       MLRAXSIFVKDRVAQAHLGVEAL---QSLVMQGDLQNFIGG---PSVDKSQY-VGCKIAV
PTHR1_human     MLRAVSIFVKDAVLYSGATLDEA---ERL-TEEELRAIAQAPPPPATAAAGY-AGCRVAV
PTHR1_RAT       MLRAASIFVKDAVLYSGFTLDEA---ERL-TEEELHIIAQVPPPPAAAAVGY-AGCRVAV
                :***  ***** *      :      :        :          .    *  **::.:

28              TLFHYFVATNYYWILVEALYLHSLIFVAFFSDKKYLWRFSVTGWGVPILFVVPWAIVRAK
105             TLFHYFVATNYYWILVEALYLHSLIFVAFFSDKKYLWRFSVTGWGVPILFVVPWAIVRAK
PTHR2_HUMAN     VMFIYFLATNYYWILVEGLYLHNLIFVAFFSDTKYLWGFILIGWGFPAAFVAAWAVARAT
PTHR2_RAT       VMFIYFLATNYYWILVEGLYLHNLIFVSFFSDTKYLWGFILIGWGFPAVFVVAWAVARAT
PTHR1_human     TFFLYFLATNYYWILVEGLYLHSLIFMAFFSEKKYLWGFTVFGWGLPAVFVAVWVSVRAT
PTHR1_RAT       TFFLYFLATNYYWILVEGLYLHSLIFMAFFSEKKYLWGFTIFGWGLPAVFVAVWVGVRAT
                .:* **:**********.****.***::***:.**** * : ***.*  **. *. .**.

28              LDDTACWDIAVTEYKWIYNGPIVVANVINFLLFLNIIRVLWYKMRERGPIGKTDNRRQYK
105             FEDTGCWDIAVTEYKWIYNGPIIVANVINFLLFLNIIRVLWYKMRERGPIGKTDNRRQYK
PTHR2_HUMAN     LADARCWELSAGDIKWIYQAPILAAIGLNFILFLNTVRVLATKIWETNAVGH-DTRKQYR
PTHR2_RAT       LADTRCWELSAGD-RWIYXXPILAAIGLNFILFLNTVRVLATKIWETNAVGH-DMRKQYR
PTHR1_human     LANTGCWDLSSGNKKWIIQVPILASIVLNFILFINIVRVLATKLRETNA-GRCDTRQQYR
PTHR1_RAT       LANTGCWDLSSGHKKWIIQVPILASVVLNFILFINIIRVLATKLRETNA-GRCDTRQQYR
                : :: **:::  . :**   **:.:  :**:**:* :***  *: * ..  *: * *:**:
```

N – N glycosylation sites (predicted from NetNglyc server [112] , ***C*** – Conserved cysteine residues

(*)- Identical , (:)- very Similar, (.) - Similar

BLUE: Transmembrane regions (predicted from GTPred [97])

28 - (ci0100139945) fragmentary model, 105 - (ci0100151327) fragmentary model

```
28             KLAKSTLVLIPMFGVHAIVFIGMPDDISSGTWWDIRMSHDLFFNSFQGFFVAIIYCFCNG
105            KLAKSTLVLIPMFGVHAIVFIGMPDDISSGTWWDIRMSHDLFFNSFQGFFVAIIYCFCNG
PTHR2_HUMAN    KLAKSTLVLVLVFGVHYIVFVCLPHS-FTGLGWEIRMHCELFFNSFQGFFVSIIYCYCNG
PTHR2_RAT      KLAKSTLVLVLVFGVHYIVFICQPHS-FSGLWWEIRMHCELFFNSFQGFFVSIVYCYCNG
PTHR1_human    KLLKSTLVLMPLFGVHYIVFMATPYTEVSGTLWQVQMHYEMLFNSFQGFFVAIIYCFCNG
PTHR1_RAT      KLLRSTLVLVPLFGVHYTVFMALPYTEVSGTLWQIQMHYEMLFNSFQGFFVAIIYCFCNG
               ** :*****: :****  **:  *    :*  *:::*  :::*********:*:**:***

28             EVQAEFRKAWERFNLSVEIKRGRRERSRSSVTMLTSFNSSASQQVRIMTS----------
105            EVQAEFRKAWERFNLSVEIKRGRRERSRSSVTMLTSFNSSASQQVRIMTS----------
PTHR2_HUMAN    EVQAEVKKMWSRWNLSVDWKRTPPCGSRRCGSVLTTVTHSTSSQSQVAASTRMVLISGKA
PTHR2_RAT      EVQAEVKKTWTRWNLSIDWKKAPPCGGHRYGSVLTTVTHSTSSQSQMGPSTRLVLISSKP
PTHR1_human    EVQAEIKKSWSRWTLALDFKRKARSGSSSYSYG-PMVSHTSVTNVGPRVGLGLPLS---P
PTHR1_RAT      EVQAEIRKSWSRWTLALDFKRKARSGSSSYSYG-PMVSHTSVTNVGPRAGLSLPLS---P
               *****.:* * *:.*::: *:     .       . .. ::  :     .

28             ------------------------------------------------------------
105            ------------------------------------------------------------
PTHR2_HUMAN    AKIASRQPDSHITLPGYVWSNSEQ----DCLPHSFHEETKED-------SGRQGDDILME
PTHR2_RAT      AKTACRQIDSHVTLPGYVWSSSEQ----DCQPQSTPEETKKG-------HGRQEDDSPVG
PTHR1_human    RLLPTATTNGHPQLPGHAKPGTPALETLETTPPAM-AAPKDDGFLNGSCSGLDEEASGPE
PTHR1_RAT      RLPP-ATTNGHSQLPGHAKPGAPATET-ETLPVTM-AVPKDDGFLNGSCSGLDEEASGSA

28             -----------------------
105            -----------------------
PTHR2_HUMAN    KPSRPMESNPDTEGCQGETEDVL
PTHR2_RAT      ESSRPVAFTIDTEGCKGESHPI-
PTHR1_human    RPPALLQ---------EEWETVM
PTHR1_RAT      RPPPLLQ---------EEWETVM
```

Residues involved in PTHR-1 coupling to Gs and their homologs in *Ciona* and PTHR-2 [102]

Residues involved in PTHR-1 coupling to Gq and their homologs in *Ciona* [103]

Residues in PTHR -1 involved in both Gs/Gq coupling ,subsequent secondary messenger generation and their homologs in *Ciona* [104]

Candidate ligand interaction/contact sites by identified by mutagenesis in PTHR -1 and their homologs in *Ciona* and PTHR -2. [105-107]

Candidate ligand interaction/contact sites in PTHR -1 identified by photo-cross linking and their homologs in *Ciona* and PTHR-2. [106, 108-111]

Residues in PTHR 1 crucial for functional interaction/agonist binding affinity/transmembrane signalling and their homologs in *Ciona and* PTHR-2 [98-99]

Residues in vertebrate PTHR1 where mutations have been associated with Blomstrand's syndrome [70-72]

Residues in Vertebrate PTHR1 where mutations have been associated with Jansen's syndrome [73-75]

Residues in PTHR-1/2 involved in conferring ligand specificity and binding and their homologs in *Ciona* [100-101]
